# A microRNA signature that correlates with cognition and is a target against cognitive decline

**DOI:** 10.15252/emmm.202013659

**Published:** 2021-10-11

**Authors:** Md Rezaul Islam, Lalit Kaurani, Tea Berulava, Urs Heilbronner, Monika Budde, Tonatiuh Pena Centeno, Vakthang Elerdashvili, Maria‐Patapia Zafieriou, Eva Benito, Sinem M Sertel, Maria Goldberg, Fanny Senner, Janos L Kalman, Susanne Burkhardt, Anne Sophie Oepen, Mohammad Sadman Sakib, Cemil Kerimoglu, Oliver Wirths, Heike Bickeböller, Claudia Bartels, Frederic Brosseron, Katharina Buerger, Nicoleta‐Carmen Cosma, Klaus Fliessbach, Michael T. Heneka, Daniel Janowitz, Ingo Kilimann, Luca Kleinedam, Christoph Laske, Coraline D Metzger, Matthias H Munk, Robert Perneczky, Oliver Peters, Josef Priller, Boris S. Rauchmann, Nina Roy, Anja Schneider, Annika Spottke, Eike J Spruth, Stefan Teipel, Maike Tscheuschler, Michael Wagner, Jens Wiltfang, Emrah Düzel, Frank Jessen, Delcode Study Group, Silvio O Rizzoli, Wolfram‐Hubertus Zimmermann, Thomas G Schulze, Peter Falkai, Farahnaz Sananbenesi, Andre Fischer

**Affiliations:** ^1^ Department for Epigenetics and Systems Medicine in Neurodegenerative Diseases German Center for Neurodegenerative Diseases Göttingen Germany; ^2^ Department for Psychiatry and Psychotherapy University Medical Center Göttingen Göttingen Germany; ^3^ Institute of Psychiatric Phenomics and Genomics (IPPG), University Hospital Ludwig‐Maximilians‐University Munich Munich Germany; ^4^ Institute of Pharmacology and Toxicology University Medical Center Göttingen Göttingen Germany; ^5^ Department of Neuro‐ and Sensory Physiology University Medical Center Göttingen Göttingen Germany; ^6^ Department of Psychiatry and Psychotherapy Ludwig‐Maximilians‐University Munich München Germany; ^7^ Department of Genetic Epidemiology University Medical Center Göttingen Göttingen Germany; ^8^ German Center for Neurodegenerative Diseases Bonn Germany; ^9^ Department of Neurodegeneration and Geriatric Psychiatry University Hospital Bonn Bonn Germany; ^10^ German Center for Neurodegenerative Diseases (DZNE, Munich) Munich Germany; ^11^ Institute for Stroke and Dementia Research (ISD) University Hospital LMU Munich Munich Germany; ^12^ Department of Psychiatry and Psychotherapy Charité – Universitätsmedizin Berlin Berlin Germany; ^13^ German Center for Neurodegenerative Diseases (DZNE) Rostock Germany; ^14^ Department of Psychosomatic Medicine Rostock University Medical Center Rostock Germany; ^15^ Section for Dementia Research Hertie Institute for Clinical Brain Research and Department of Psychiatry and Psychotherapy University of Tübingen Tübingen Germany; ^16^ German Center for Neurodegenerative Diseases (DZNE) Magdeburg Germany; ^17^ Institute of Cognitive Neurology and Dementia Research (IKND) Otto‐von‐Guericke University Magdeburg Germany; ^18^ Department of Psychiatry and Psychotherapy Otto‐von‐Guericke University Magdeburg Germany; ^19^ German Center for Neurodegenerative Diseases (DZNE) Tübingen Germany; ^20^ Munich Cluster for Systems Neurology (SyNergy) Munich Munich Germany; ^21^ Ageing Epidemiology Research Unit (AGE) School of Public Health Imperial College London London UK; ^22^ German Center for Neurodegenerative Diseases (DZNE) Berlin Germany; ^23^ Department of Psychiatry and Psychotherapy Charité Berlin Germany; ^24^ Department of Neurology University of Bonn Bonn Germany; ^25^ Department of Psychiatry Medical Faculty University of Cologne Cologne Germany; ^26^ German Center for Neurodegenerative Diseases (DZNE) Göttingen Germany; ^27^ Department of Medical Sciences Neurosciences and Signaling Group Institute of Biomedicine (iBiMED) University of Aveiro Aveiro Portugal; ^28^ Excellence Cluster on Cellular Stress Responses in Aging‐Associated Diseases (CECAD) University of Cologne Köln Germany; ^29^ Cluster of Excellence “Multiscale Bioimaging: from Molecular Machines to Networks of Excitable Cells” (MBExC) University of Göttingen Göttingen Germany; ^30^ DZKH (German Center for Cardiovascular Diseases) Göttingen Germany; ^31^ German Center for Neurodegenerative Diseases, Research Group for Genome Dynamics in Brain Diseases Göttingen Germany

**Keywords:** Alzheimer, biomarker, cognitive impairment, microRNA, RNA therapeutics, Biomarkers, Neuroscience, RNA Biology

## Abstract

While some individuals age without pathological memory impairments, others develop age‐associated cognitive diseases. Since changes in cognitive function develop slowly over time in these patients, they are often diagnosed at an advanced stage of molecular pathology, a time point when causative treatments fail. Thus, there is great need for the identification of inexpensive and minimal invasive approaches that could be used for screening with the aim to identify individuals at risk for cognitive decline that can then undergo further diagnostics and eventually stratified therapies. In this study, we use an integrative approach combining the analysis of human data and mechanistic studies in model systems to identify a circulating 3‐microRNA signature that reflects key processes linked to neural homeostasis and inform about cognitive status. We furthermore provide evidence that expression changes in this signature represent multiple mechanisms deregulated in the aging and diseased brain and are a suitable target for RNA therapeutics.

## Graphical abstract

Alzheimer’s disease (AD) is usually diagnosed at an advanced stage of molecular pathology, a time point when causative treatments fail. This study aimed to identify a minimally invasive biomarker that can help to identify individuals at risk for cognitive decline before clinical manifestation.

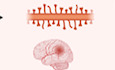

**The paper explained**

**Problem**

The establishment of effective therapies for age‐associated neurodegenerative diseases such as Alzheimer’s disease (AD) is still challenging because pathology accumulates long before there are any clinical signs of disease. Thus, patients are often only diagnosed at an already advanced state of molecular pathology, when causative therapies fail. Therefore, there is an urgent need for molecular biomarkers that are (i) minimally invasive, (ii) can inform about individual disease risk, and (iii) ideally indicate the presence of multiple pathologies. Such biomarkers should eventually be applicable in the context of routine screening approaches with the aim to detect individuals at risk for developing AD that could then be subjected to further diagnostics via more invasive and time‐consuming examinations.

**Results**

We use a novel experimental approach combining the analysis of young and healthy humans with already diagnosed patients as well as animal and cellular disease models to eventually identify a 3‐microRNA signature that can inform about the risk of cognitive decline when measured in blood. The 3‐microRNA signature also informs about relevant patho‐mechanisms in the brain, and targeting this signature via RNA therapeutics can ameliorate AD disease phenotypes in animal models.

**Impact**

We suggest that the analysis of this microRNA signature could be used as point‐of‐care screening approach to detect individuals at risk for developing AD that can then undergo further diagnostics to allow for early and effective intervention. In addition, our data highlight the potential of stratified RNA therapies to treat Alzheimer’s disease.

## Introduction

Impaired cognitive function is a key hallmark of age‐associated neurodegenerative diseases and is often one of the first clinical symptoms. However, changes in cognitive function develop slowly over time and while some individuals develop pathological memory impairment, others exhibit preserved cognitive function until old age, a phenomenon that has been referred to as cognitive reserve (Stern, 2012). As a result, pathological memory decline is often only diagnosed at an already advanced stage of molecular pathology. *Bona fide* examples are age‐associated neurodegenerative diseases such as Alzheimer’s disease (AD), the most common form of dementia in the elderly. The failure to detect risk individuals at an early stage of molecular pathology is considered to be a major reason why, for example, causative treatments for AD have so far failed in clinical trials (Schneider *et al*, 2014; Abbott & Dolgin, 2016). Thus, there is an urgent need for molecular markers that could inform about subtle changes in cognitive status, with the aim to detect individuals that are at risk for developing dementia to allow for earlier interventions. In order to be applicable in the context of routine check‐up screenings in a primary care setting, such markers need to be comparatively inexpensive, easy to screen, and predictive as to the identification of the individuals at risk. Such individuals could then be subjected to further diagnostics via more invasive and time‐consuming examinations such as the analysis of cerebrospinal fluid (CSF) as well as functional and structural brain imaging (Molinuevo *et al*, 2018). Recent data suggest that biomarkers reflecting, for example, specific pathologies linked to neurodegenerative conditions might also be measured in blood (Olsson *et al*, 2016; Blennow, 2017; Jack *et al*, 2018; Li & Mielke, 2019), and indeed, blood would be a suitable body fluid for screening approaches. However, age‐associated cognitive diseases are multifactorial. Thus, in addition to marker for specific pathologies, there is an additional need for molecular biomarker that could inform about the variable combinations of environmental and genetic factors that affect cognitive reserve and the progression to age‐associated cognitive decline. A recent line of research indicates that circulating microRNAs might serve as diagnostic biomarker for various disorders (Witwer, 2015), including brain diseases (Rao *et al*, 2013; Galimberti *et al*, 2014; Hill & Lukiw, 2016; Kumar *et al*, 2017). MicroRNAs are 19–22 nucleotide long RNA molecules regulating protein homeostasis via binding to a target mRNA, thereby causing its degradation or inhibition of translation (Gurtan & Sharp, 2013). MicroRNAs are particularly interesting as potential biomarker since changes in a few microRNAs can reflect complex alterations in cellular homeostasis and could therefore indicate the presence of multiple pathologies (Zampetaki *et al*, 2012; Fischer, 2014a; Condrat *et al*, 2020). Moreover, microRNAs are extremely stable in cell‐free environments; are resistant to thaw–freeze cycles (Mitchell *et al*, 2008; Zampetaki *et al*, 2012; Rao *et al*, 2013); and have been implicated with learning and memory function, dementia, and AD (Hébert *et al*, 2008; Zovoilis *et al*, 2011; Aksoy‐Aksel & Schratt, 2014; Jaber *et al*, 2019). In addition, RNA therapeutics is emerging as a promising approach to treat CNS diseases (Roovers *et al*, 2018) and there is evidence that microRNAs may be useful targets for stratified RNA‐based therapies (Zovoilis *et al*, 2011; Banzhaf‐Strathmann *et al*, 2014; Salta & De Strooper, 2017; Hanna *et al*, 2019). Despite these promising data, the identification of a microRNA panel that could inform about cognitive status, help to detect patients at risk for developing cognitive impairment, and would serve as a drug target has been challenging. In this study, we combine the analysis of model systems and human cohorts to identify a circulating 3‐microRNA signature that informs about differences in cognitive function and could help to identify patients at risk for developing dementia. We furthermore provide evidence that the signature informs about multiple patho‐mechanisms linked to cognitive decline and is a suitable target for RNA therapeutics.

## Results

### Identification of circulating microRNAs linked to cognitive function in healthy humans

**Figure EV1:**
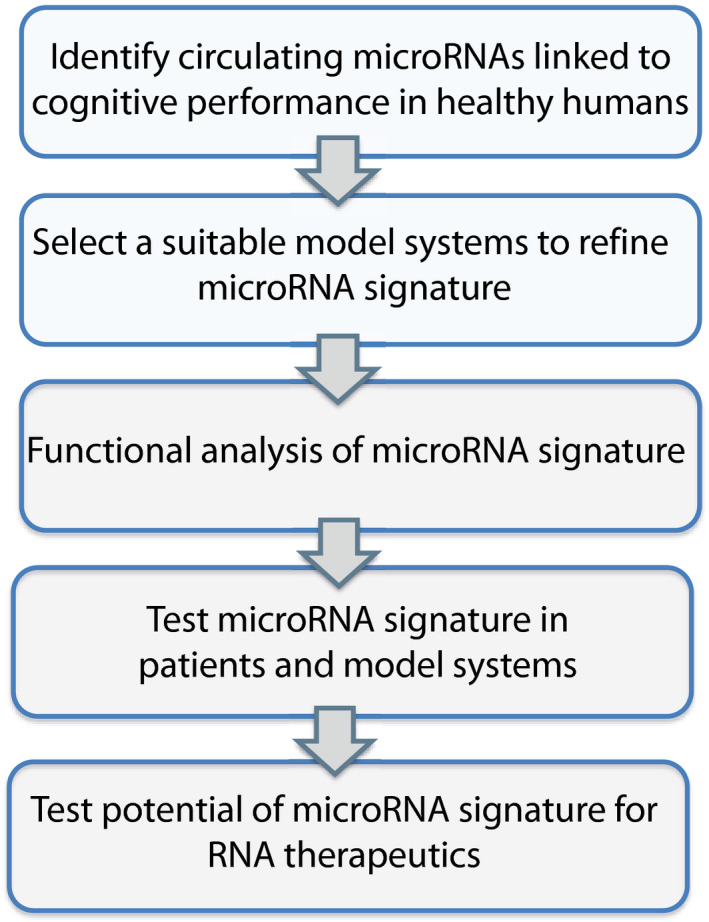
Experimental approach The scheme displays our overall experimental approach and the various filtering steps to identify circulating microRNAs that informs about the cognitive reserve and would allow the early detection of patients at risk for developing cognitive decline. We hypothesized that a promising approach would be to first identify circulating microRNAs that correlate with memory performance in young and healthy humans. Based on the function of such microRNAs, we then thought to employ model systems to further refine these data and develop a microRNA signature that could then be analyzed at the functional level and be eventually tested in patients and disease models. We also planned to test the potential of such a microRNA signature for RNA therapeutics with the aim to ameliorate cognitive decline.

**Figure 1:**
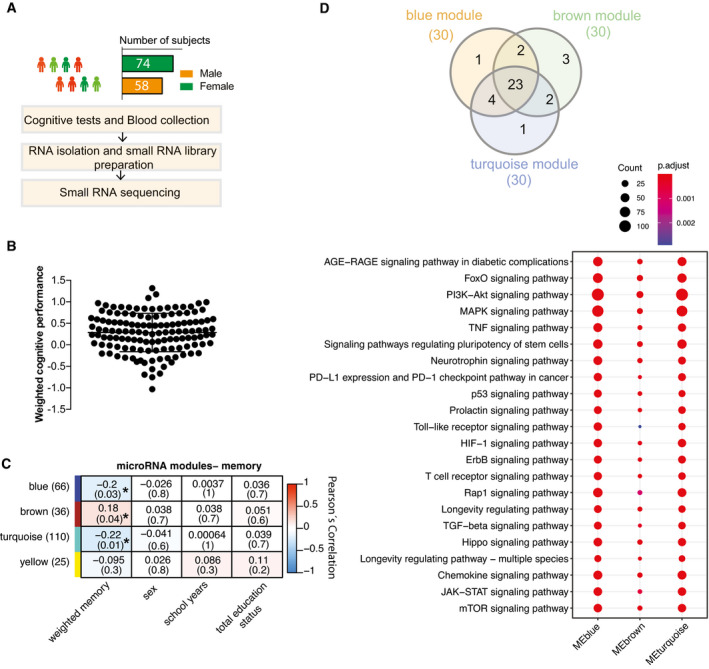
Circulating microRNA expression levels correlate with cognitive function in healthy humans Experimental approach for the detection of blood microRNAs that correlate with memory function in healthy humans. This cohort includes 132 healthy and young individuals (74 males and 58 females, age: 25.95 ± 5.1 years). Participants took part in cognitive tests and donated blood samples (PAXgene tubes).Weighted cognitive score of the 132 individuals shows the expected variability. Bar and error bars indicate mean ± SD.MicroRNAs having at least 5 reads in 50% of the samples were considered for downstream co‐expression analysis. Co‐expression analysis revealed 3 microRNA clusters that were significantly linked to weighted cognitive performance. Number of microRNAs in each module is given in parentheses next to module name. Co‐expression modules are represented in rows, while each column refers to a phenotypic trait. Each cell contains the corresponding correlation coefficient and *P*‐value (denoted inside parentheses). Color code represents Pearson’s correlation. Expressions of the modules are not correlated with sex, number of years at school, or status of total education.The analysis of the experimentally validated mRNA targets of the microRNAs belonging to 3 clusters identified in (C). Downstream analyses on those genes reveal that they control pathways related to aging and age‐related functions known to play a role in cognition. Venn diagram displays 23 of the top 30 significant pathways are common among three modules. Dot plot represents the top 23 common significant pathways across three modules. Size of the dot represents number of genes belonging to each pathway term while the color represents the statistical significance.

A valuable approach to identify molecular biomarker is the comparison of healthy individuals and patients. The fact that this comparison depends on prior measures used to make a diagnosis could be a particular problem to identify people at risk of age‐associated cognitive diseases, since molecular changes often occur years before clinical symptoms manifest and the diagnosis is made (Stern, 2009; Beason‐Held *et al*, 2013; Sperling *et al*, 2014). Therefore, we decided to use an alternative approach, aiming to identify molecular marker that correlates with subtle differences in the cognitive status in healthy individuals. As a starting point, we took advantage of the fact that cognitive abilities vary in young healthy individuals (Deary *et al*, 2009). We hypothesized that microRNAs linked to inter‐individual differences in cognition among healthy individuals might be a useful starting point to identify molecular marker for cognitive function, with the aim to subsequently refine candidate microRNAs via different filtering approaches including the analysis of suitable model systems and patients (Fig EV1). In a pilot experiment, we compared various methods of blood collection and RNA isolation followed by small RNA sequencing. We concluded that the collection of blood via PAXgene tubes (Qiagen) is a suitable approach that also allows the comparable analysis of human and mouse blood and thus would benefit the cross‐correlation of human data and mechanistic studies performed in disease models (Appendix Fig S1). Since previous studies suggest that specific cognitive abilities start to decline in humans already in the late 30s (Schaie, 1993; Park *et al*, 2002), we recruited young healthy individuals (age: 25.95 ± 5.1 years, mean ± SD, *n* = 132; Dataset EV1) that were subjected to a battery of six different cognitive tests (Budde *et al*, 2018). Total blood was collected via Pax gene tubes from all participants at the time of memory testing, and small RNAome sequencing was conducted (Fig 1A). To identify microRNAs that correlate with cognition, we first calculated for each of the 132 individuals a composite cognitive score (weighted cognitive performance) based on factor analysis which confirmed the expected variability in cognitive function (Fig 1B). Next, after adjusting sequencing data for sex effect, we performed a weighted microRNA co‐expression analysis to identify expression modules. We then asked whether any of these microRNA modules correlate with the weighted cognitive performance and detected 4 modules, of which 3 modules were significantly linked to cognition (Fig 1C, Appendix Fig S2). The turquoise and blue modules were negatively correlated with cognitive performance while the brown module showed a positive correlation with cognition (Fig 1C, Dataset EV2). Of note, the expression of these modules did not correlate to number of years in school or status for total education (e.g., high school education + professional degree) (Fig 1C). Next, we performed a KEGG‐pathway analysis for the confirmed mRNA targets of the 3‐microRNA modules (Dataset EV3). While care has to be taken when interpreting such data, we reasoned that such analysis would be a suitable first approach to help us design further experiments, with the aim to eventually define more specific candidate microRNAs. We selected the top 30 significant pathways (adjusted *P*‐value < 0.05) from each individual cluster and then asked whether these pathways would also be detected within the other clusters. When considering the common pathways, we identified 23 highly significant pathways that were detected across all 3 clusters (Fig 1D, Dataset EV3). We noticed that many of these pathways reflect biological functions linked to age‐associated memory impairment such as the mTOR signaling (Heras‐Sandoval *et al*, 2011), stem cell function (Oh *et al*, 2014), the AGE‐RAGE pathway (Frimat *et al*, 2017), MAPK signaling (Huentelman *et al*, 2018), or immune‐related functions such as TNF‐alpha signaling (Lindbergh *et al*, 2020). This observation is in line with the view that aging is the major risk factor for cognitive decline and dementia. Moreover, since accelerated or aberrant brain aging has been discussed as a process linked to dementia (Franceschi *et al*, 2018), our observation also hints to the possibility that even in young and healthy individuals, differences in cognitive function might be mechanistically linked to subtle changes in molecular processes associated with the aging process. On the basis of this hypothesis, we reasoned that cognitive aging could be a *bona fide* experimental approach to further filter the detected microRNAs for molecular biomarker candidates that reflect cognitive status.

### Identification of circulating microRNAs in longitudinal mouse model for age‐associated memory decline

**Figure 2:**
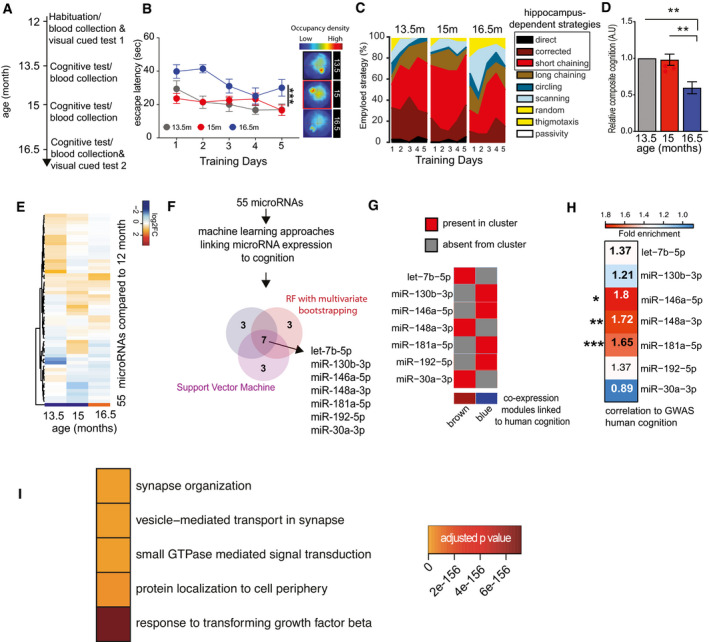
Identification of circulating microRNAs informative about memory decline in mice Experimental design of the water maze experiment. At 12 months of age, male mice were subjected to the water maze training protocol in order to habituate the animals to the procedure. Subsequently, mice were subjected to water maze training followed by a probe test at 13.5, 15, and 16.5 months of age. The platform position was altered during each training procedure. Blood was collected upon completion of each water maze procedure. A visual cued test was performed after the first and after the last blood collection when mice were 12 and 16.5 months of age, respectively.Escape latency during water maze training when mice were 13.5, 15, or 16.5 months of age (*n* = 10 each group). Two‐way ANOVA followed by Tukey´s multiple comparisons test revealed significant effects of training trials (*P*‐value < 0.0001) and age (***P‐value 0.0004) on the performance. On days 1 and 2, there was a significant (*P*‐value < 0.05) difference between mice at 13.5 versus 16.5 months and 15 versus 16.5 months of age (*n* = 10 each group). On the 5^th^ day of training, there was a significant difference observed between 15 and 16.5 months of age. Density plot (Right) shows the occupancy pattern of mice at different time points of aging. Occupancy signal on the platform was the least at 16.5 months, suggesting mice at the given age failed to locate the platform.Analysis of the different search strategies during the water training sessions. Note that especially at 16.5 months of age mice adopt hippocampal independent search strategies indicative of impaired cognitive function.The cumulative cognitive score calculated for each day on the basis of hippocampal‐dependent strategies was significantly impaired when comparing mice at 16.5 months of age to their performance at 15 or 13.5 months of age. Data are normalized to 13.5‐month group (ordinary one‐way ANOVA, Tukey`s multiple comparison test). *N* = 10 mice/group, **P < 0.01.Heat map showing the expression pattern of 55 aging responsive microRNAs significantly deregulated during the course of aging. All data are shown in comparison with the expression level at 12 months of age. All microRNAs having at least 100 reads in 50% of the samples were filtered prior to differential expression analysis.Expressions of these 55 microRNAs and cognitive performances along aging were used to identify microRNA features linked to cognition. Three independent approaches [e.g., random forests (RF) with leave‐one‐out cross‐validation, RF with multivariate bootstrapping, and support vector machine (SVM)] find seven common microRNAs that explain cognitive variability assayed in the water maze test.Color map showing that the 7 microRNAs identified in (F) are present in the co‐expression modules significantly linked to cognition in healthy humans as described in Fig 1.Heat map showing the enrichment of mRNA targets of the 7 microRNAs shown in (G) with a gene set identified by GWAS studies linking genes to cognition in healthy humans (Davies & Harris, 2018). Note that target genes of miR‐181a‐5p, miR‐148a‐3p, and miR‐146a‐5p are significantly overlapped in a hypergeometric test (fold enrichment > 1.5 and FDR <0.05). *P = 0.02, **P = 0.0008, ***P = 2.267e‐05.Gene ontology analyses of miR‐181a‐5p, miR‐148a‐3p, and miR‐146a‐5p predicted targets reveal top significant processes linked to neuronal function and inflammation. Heatmap represents the top 5 significant biological processes. Color code represents adjusted *P*‐value. Data information: Bars and error bars indicate mean ± SEM.

To further filter the microRNAs within the clusters, we wanted to employ relevant model system that would allow us to study the blood and brain. Although no animal model can fully recapitulate cognitive diseases in humans, age‐associated memory decline is a well‐established and highly reproducible phenotype observed in laboratory rodents and in humans and affects similar brain regions such as the hippocampus (Wolf *et al*, 2001; Peleg *et al*, 2010; Fjell *et al*, 2014; Duzel *et al*, 2016; Dicks *et al*, 2019). Moreover, our pilot data showed that the highly expressed circulating microRNAs common in mice and humans are comparable in expression and that we can reliably measure circulating microRNAs in living mice (see Appendix Fig S1). Thus, we decided to employ mice as a model for age‐associated memory decline for our further analysis, since this would also allow us to perform longitudinal and eventually mechanistic experiments. We reasoned that the subsequent comparison of human and experimental data from aging mice could help to further filter the list of microRNAs for potential molecular biomarkers of cognitive status. To study cognition in mice, we employed the Morris water maze paradigm, which is a well‐established test for spatial reference memory and furthermore enables the sensitive and repeatable measure of several comparable cognitive domains in mice and in humans (Havas *et al*, 2011; Illouz *et al*, 2016; Laczó *et al*, 2017). In cross‐sectional studies, impairment of spatial reference memory in mice can be detected at 16 months of age when compared to 3‐month‐old mice, while the comparison of 3‐ versus 12‐month‐old mice did not reveal differences among age‐groups (Peleg *et al*, 2010; Stilling *et al*, 2014). Thus, we hypothesized that analyzing mice from 12 until 16.5 months of age should allow us to detect cognitive decline in a longitudinal setting. To avoid any effect associated with the first exposure to the water maze paradigm, 12‐month‐old mice were habituated to the training procedure. Subsequently, all mice were subjected to water maze training followed by a memory test and blood collection every 1.5 months (Fig 2A). Importantly, the longitudinal collection of blood from the orbital sinus of mice did not affect vision in the water maze paradigm, which is a pre‐requisite to perform the test (Appendix Fig S3). We also collected blood in a group of aging mice that were not subjected to memory training in order to control for effects that the training may have on the circulating microRNAome (Appendix Fig S4A). The escape latency during the training procedure—a common measure of spatial learning ability—was significantly impaired when comparing mice at 16.5 months of age to their performance at 13.5 or 15 months of age (Fig 2B). For a more sensitive analysis, we employed a modified version of the MUST‐C algorithm to measure the different spatial strategies that represent hippocampus‐dependent and cognitively demanding as well as hippocampus‐independent strategies (Illouz *et al*, 2016). Our results indicate that specifically between 15 and 16.5 months of age, mice adopt search strategies indicative of impaired cognitive ability (Fig 2C). Thus, there was a notable reduction in “direct”, “corrected”, and “short chaining” search strategies that reflect hippocampus‐dependent cognitive functioning. On the other hand, strategies independent of hippocampus‐dependent cognitive ability such as “long chaining”, “circling”, and “random” increased at 16.5 months of age (Fig 2C). In line with this observation, the cognitive score, which was calculated on the basis of the different search strategies during the training procedure, was significantly reduced when comparing mice at 16.5 months to their performance at either 13.5 or 15 months of age (Fig 2D). Similar data were obtained in the probe test to assay memory retrieval (Appendix Fig S5). Since all mice were able to find the platform rapidly in a visually cued test performed at 12 and 16.5 months of age (see Appendix Fig S3), and also the analysis of swimming speed did not reveal any significant differences, these data show that age‐associated defects in spatial reference learning can reliably be detected in mice between 13.5 and 16.5 months of age. Next, we subjected blood‐derived RNA collected from at all time points to small RNA sequencing. The corresponding data were filtered (microRNAs > 100 reads in 50% samples) and fit to a likelihood ratio test model and adjusted for hidden confounding factors to detect microRNAs that were differentially expressed during the aging process using the expression at 12 months of age as a reference point.

We also controlled for microRNAs that were potentially affected by the training procedure applying a similar analytical workflow (Appendix Fig S4A–C). Thereby, we detected 55 differentially expressed circulating microRNAs during aging (Fig 2E, Appendix Fig S4, Dataset EV4). To identify among these 55 microRNAs the ones that would inform best about cognitive status and cognitive decline, we employed the sequencing and cognitive data to perform an unbiased multivariate microRNA feature selection. First, we constructed a composite score from the different water maze features using principal component analysis (PCA). To identify microRNAs that inform about memory performance, we subjected these scores along with the expression data of the 55 microRNAs to 3 independent methods for feature selection, namely random forest approaches using multivariate bootstrapping or multivariate leave‐one‐out cross‐validation (Looc) and a support vector machine approach. Importantly, all 3 methods identified a common 7‐microRNA signature linked to age‐associated memory performance consisting of let‐7b‐5p, miR‐181a‐5p, miR‐146a‐5p, miR‐192‐5p, miR‐30a‐3p, miR‐148a‐3p, and miR‐130b‐3p (Fig 2F, Dataset EV5). These seven microRNAs were also among the age‐related differentially expressed microRNAs with high expression (average read counts: > 100), when an alternative differential expression analytical approach with low filtration (> 1 read in 50% samples) was performed (Appendix Fig S4D, Dataset EV4). All 7 microRNAs were part of either the brown or blue co‐expression module that was significantly correlated to cognitive performance in healthy humans (Fig 2G). These data suggest that the 7 microRNAs are *bona fide* candidates for circulating biomarkers of cognitive status and reserve. Encouraged by these findings, we decided to further validate the significance of the 7 microRNAs using another human dataset. Recent GWAS studies identified 709 genes that are associated with general cognitive function in healthy individuals (Marioni *et al*, 2018). When we asked whether target genes of any of the 7 microRNAs would be enriched among the 709 genes linked to cognition in humans (Dataset EV6), we observed that targets of miR‐181a‐5p, miR‐146a‐5p, and miR‐148a‐3p were significantly overrepresented (Fig 2H). Further analyses suggested that the top 5 significant biological processes affected by the predicted targets of these 3 microRNAs (Dataset EV7) are linked to neuronal plasticity (e.g., synapse organization, vesicle‐mediated transport in synapse), GTPase‐mediated signal transduction, protein localization to cell periphery, and the response to transforming growth factor beta (TFG‐beta) (Fig 2I, Dataset EV8). This finding suggests that the 3 microRNAs can control key processes that are linked to cognitive function that are deregulated during age‐associated cognitive decline, including synaptic function and inflammatory processes. Furthermore, comparing the list of predicted target genes to the SynGO database (Koopmans *et al*, 2019) revealed a significant enrichment for pre‐ and postsynaptic processes (Dataset EV9). Nevertheless, care has to be taken when interpreting an analysis based on predicted target genes. These results should therefore be viewed as an exploratory approach to guide further experiments.

### Elevated levels of the 3‐microRNA signature are linked to impaired neuronal integrity

**Figure 3:**
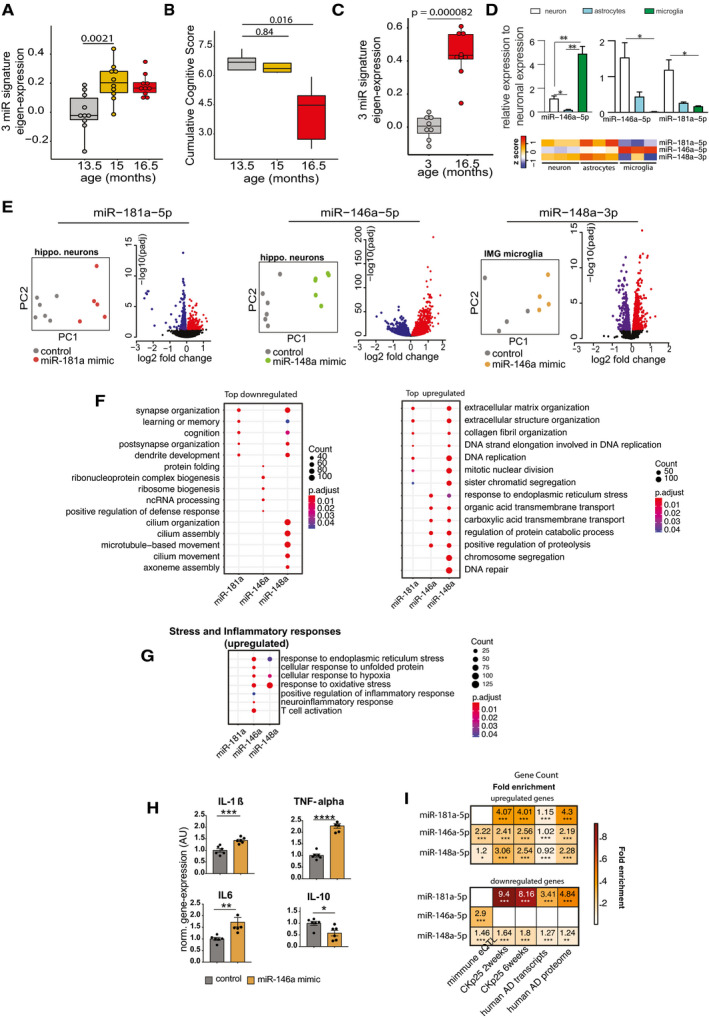
Expression changes in 3‐miR signature reflect aberrant neuronal and immune processes Eigenvalue of the 3‐microRNA signature measured in the mice that performed longitudinal water maze training. Note the significantly increased expression of the signature already at 15 months of age, suggesting that increased expression levels precede detectable cognitive impairment. Number of biological replicates = 10/group, unpaired two‐sided Wilcoxon rank test.Cognitive score measured in the same mice reveals cognitive decline between 15 and 16,5 months of age (*P*‐value 0.0079). Number of biological replicates = 10/group, unpaired two‐sided Wilcoxon rank test.Eigenvalue showing the expression of the 3‐microRNA signature in the hippocampus of 3 and cognitively impaired 16.5‐month‐old mice. Number of biological replicates (3 months = 8, 16.5 months = 9), unpaired two‐sided Wilcoxon rank test.(Top) Relative enrichment of the three microRNAs across different cell types. Quantitative expression of microRNAs in primary hippocampal neurons, primary astrocytes, and primary microglia. miR‐146a‐5p is significantly enriched in microglia, while miR‐148a‐3p and miR‐181a‐5p are significantly enriched in neurons. *N* = 5/group, Two‐way ANOVA, Tukey's multiple comparisons test, *P < 0.05, **P < 0.01. Bars and error bar indicate mean ± SEM. (Bottom) miRNA expression in different cell types of mouse brain stem. The data were retrieved from Hoye *et al* (2017).Overexpression of microRNAs in relevant cell types for 48 h followed by genome wide RNA‐seq analysis. miR‐181a‐5p was overexpressed in primary hippocampal neurons, while miR‐146a‐5p was overexpressed in microglia culture. Immortalized microglial cell line was used for this purpose. Given that miR‐148a‐3p was highly enriched in neurons (D), primary hippocampal neurons were treated with the corresponding miR‐148a‐3p mimic. PCA plot shows that the mimic‐ and control‐treated samples cluster distinguishingly separate from one another. Volcano plot displays the genes significantly deregulated in mimic‐treated samples compared with control samples (FDR < 0.05). Red color indicates the upregulated genes while the blue color represents the genes those were downregulated.Gene ontology analyses for up‐ and downregulated genes. Panel F summarizes top significant up‐ and down‐regulated biological processes corresponding to each microRNA and comparison among them. Overexpression of miR‐181a‐5p and miR‐148a‐3p led to downregulation of genes related to cognition and synaptic functions, while downregulated genes due to increased expression of miR‐146a‐5p represent ncRNA processing, defense response, and protein folding mechanisms. The upregulated genes due to overexpression of these microRNAs represent several processes including extracellular matrix, endoplasmic reticulum stress.Comparison of increased stress and inflammatory responses related significant biological processes among microRNAs. Interestingly, miR‐146a‐5p overexpression in microglia led to increased expression of inflammatory‐related genes. Overexpression of both miR‐146a‐5p and miR‐148a‐3p can increase stress‐related responses. Size of the dot represents the number of genes belonging to the given process, and the color represents the *P*‐value after multiple corrections.qPCR analysis confirms the overexpression of pro‐inflammation‐related genes (IL‐1β, IL‐6, TNF‐alpha) due to overexpression of miR‐146a‐5p. Expression of anti‐inflammatory gene, IL‐10 was downregulated in mimic‐treated cells compared with the controls. Unpaired *t*‐tests, two‐tailed, ****P < 0.0001, ***P < 0.001, **P < 0.01, *P < 0.05. Bars and error bar indicate mean ± SEM. Number of biological replicates: 5–6/group.Hypergeometric overlap of the up‐ and downregulated genes (E) with gene sets from different datasets. We calculated enrichment of the deregulated genes relative to those gene sets and used a Fisher's exact test *P*‐value after multiple adjustments to estimate the significance of the overlap. Immune‐related genes based on expression quantitative loci (eQTL) were retrieved from a previous study. RNA‐seq data from CK‐p25 mice at 2 and 6 weeks after induction were retrieved from GSE65159, and up‐ and downregulated genes compared with littermate controls were determined after differential expression (significant genes; adjusted *P*‐value<0.05). Up‐ and downregulated transcripts in human AD patients compared with control subjects were determined by analysis of the available data (GSE44770). Proteins those are over‐ and reduced‐expressed in AD patients compared with controls were retrieved from a previous study. Overlap analysis for the up‐ and downregulated genes due to overexpression of microRNAs was performed to those separately from the disease conditions. Human orthologs of the mouse deregulated genes were used to perform the overlap analysis in human datasets. Color code represents fold enrichment. *FDR < 0.05, **FDR < 0.01, ***FDR < 0.001. Data information: In the boxplots in (A, B, C), the centerline indicates the median, while the upper and lower lines represent the 75^th^ and 25^th^ percentiles, respectively. The whiskers represent the smallest and largest values in the 1.5× interquartile range.

**Figure EV2:**
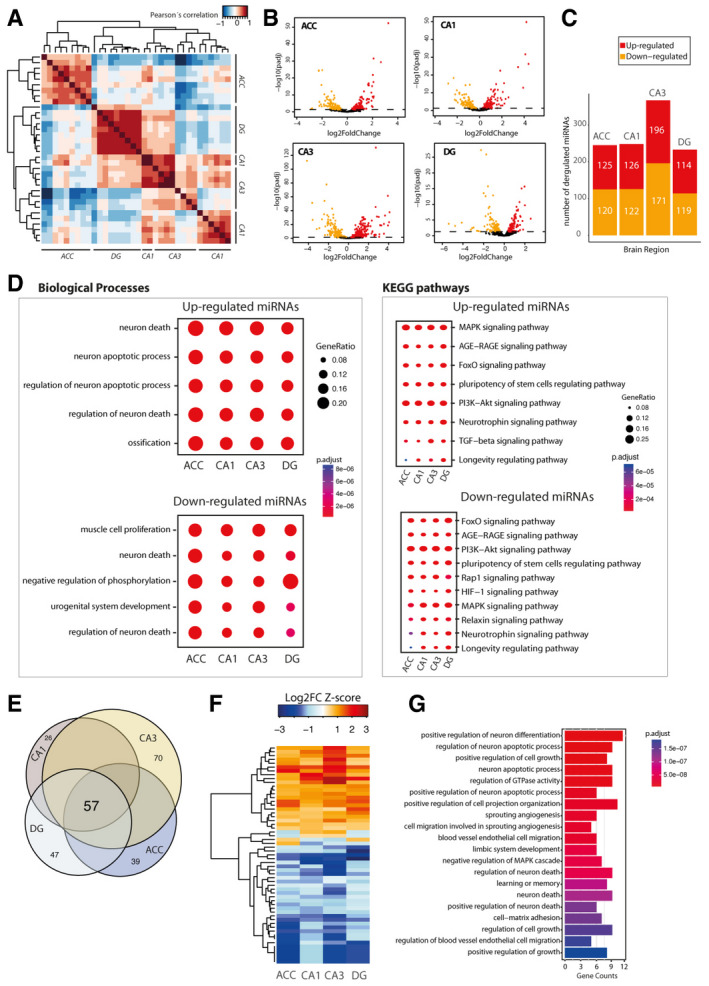
The microRNAome of learning‐related brain regions in the aging mouse brain We performed small RNA sequencing of the hippocampal sub‐regions CA1, CA3, and dentate gyrus (DG) and the anterior cingulate cortex (ACC) isolated from young (3 months of age) and cognitively impaired old (16.5 months of age) mice. APearson’s coefficient‐based correlation followed by unsupervised clustering of all small RNA‐seq data from different brain regions (ACC; CA1; CA3; DG) in young mice reveals brain region‐specific expression of the microRNAs that were particularly obvious for the ACC versus the hippocampal sub‐regions and the DG versus CA1 and CA3. These region‐specific differences were, however, mainly attributed to different expression values since the majority of the microRNAs, namely 176 microRNAs, could be detected at reliable levels in all investigated brain regions.BVolcano plots showing differential expression of microRNAs in the different brain regions when comparing young versus old mice.CBar plots showing the number of up and downregulated microRNAs in the investigated brain regions.DComparison of gene ontology and functional pathways of experimentally confirmed target genes of the deregulated microRNAs in the investigated brain regions. Pathway is generally linked to neuronal death and longevity pathways.EVenn diagram showing that 57 microRNAs are commonly deregulated in the aging brain.FHeat map showing hierarchical clustering of the 57 commonly deregulated microRNAs based on Log2 fold change Z‐score.GTop 20 biological processes affected by the confirmed target genes of the 57 commonly deregulated microRNAs in the aging brain.

We decided to further explore for the role of the 3 microRNAs in cognitive function and their relevance in the predicted biological processes. First, we tested whether the 3‐microRNA signature would indeed help to detect differences in cognitive function in our longitudinal mouse model for age‐associated memory decline. Therefore, we devised a statistical framework to test the co‐expression of the 3 microRNAs using its eigen expression, which represents a solid method to decompose gene expression data into a singular value based on linear transformation (Alter *et al*, 2000). The eigen expression of the 3‐microRNA signature significantly increased in aging mice between 13.5 and 15 months of age and plateaued at 16.5 months of age (Fig 3A). Since significant learning impairment was observed only upon 16.5 months of age (Fig 3B; See also Fig 2), these data indicate that increased expression of the 3‐microRNA signature precedes detectable memory impairment in aging mice. The 3 microRNAs of the identified signature are also highly expressed in the brain (Ludwig *et al*, 2016) and the fact that they were also linked to brain‐related processes prompted us to test their role in the brain directly. To this end, we performed small RNA sequencing of the hippocampal sub‐regions CA1, CA3, and dentate gyrus (DG) and the anterior cingulate cortex (ACC) isolated from 3‐ and 16.5‐month‐old mice (Fig EV2). Similar to the data obtained in blood samples, the expression of the 3‐microRNA signature was significantly increased in the brains of cognitively impaired 16.5‐month‐old mice (Fig 3C, Appendix Fig S6). These data support our hypothesis that altered blood levels of the 3 microRNAs may inform about relevant patho‐mechanisms in the brain. To investigate this further, we analyzed cell type‐specific expression of the three microRNAs using primary cell cultures and found that that miR‐181a‐5p is highly expressed in neurons, which is in line with its reported role in synaptic plasticity (Saba *et al*, 2012; Stepniak *et al*, 2015) (Fig 3D). miR‐148a‐3p is also enriched in neurons, and its increased expression has been associated with neurodegenerative conditions (Wang *et al*, 2016; Chen *et al*, 2019), while miR‐146a‐5p is relatively more enriched in microglia, but still expressed in neurons (Fig 3D), which agrees with previous data reporting a role of this microRNA in inflammatory processes (Maschmeyer *et al*, 2018; Mitjans *et al*, 2018). This expression pattern of the 3 microRNAs was confirmed when we analyzed previously published small RNA‐seq datasets for corresponding cell types. Our finding that the 3 microRNAs are correlated to cognitive function in healthy humans and increase prior to age‐associated memory decline in aging mice suggests that their elevated level might be detrimental. Thus, we decided to test the impact of the 3 microRNAs in the relevant cell types by increasing their levels via lipid nanoparticles containing the corresponding mimic oligonucleotides. Based on the relative enrichment in the different neural cells, we administered miR‐181a‐5p and miR‐148‐3p mimics to hippocampal neuronal and miR‐146a‐5p to immortalized microglia cultures and subsequently performed RNA sequencing. We observed substantial changes in gene expression (Fig 3E). Gene ontology analysis revealed that the top significant processes related to downregulated genes were linked to neuronal plasticity and learning and memory in case of miR‐181a‐5p and miR‐148‐3p mimics (Fig 3F, Dataset EV10). Increasing miR‐146a‐5p levels in the microglia culture caused the downregulation of genes linked to ncRNA processing and protein folding (Fig 3F, Dataset EV10). When we analyzed the upregulated genes, we observed that miR‐181a‐5p and miR‐148‐3p affected gene linked to the extracellular matrix, while the top 5 increased biological processes in response to elevated levels of miR‐146a‐5p were linked to endoplasmic reticulum stress and metabolic functions (Dataset EV10). Since miR‐146a‐5p was enriched in microglia cells, we also specifically analyzed upregulated stress and immune‐related biological processes. We observed several significant processes related to cellular stress and inflammation that were increased in response to treatment with miR‐148a‐3p and especially miR‐146a‐5p mimics (Fig 3G). That increased level of miR‐146a‐5p can contribute to inflammation‐related processes was further confirmed by qPCR showing the upregulation of the pro‐inflammatory cytokines IL‐1beta, IL‐6, and TNF‐alpha, while the anti‐inflammatory cytokine IL‐10 was decreased (Fig 3H). In sum, these data support the hypothesis that increased levels of the 3 microRNAs represent multiple mechanisms linked to a low cognitive reserve and a risk to develop cognitive decline. To further substantialize this finding, we compared our gene expression data to transcriptome and proteome datasets previously linked to neurodegenerative diseases. Of course, care has to be taken when interpreting the comparison of datasets that have been generated via different experimental platforms. Especially, microarray studies are biased by probe design, while RNA‐seq is characterized by a wider dynamic range. Interestingly, the genes deregulated in response to miR‐146a‐5p overexpression significantly overlapped with immune response genes recently reported by eQTL analysis (Gjoneska *et al*, 2015) confirming a role of miR‐146a‐5p in neuroinflammation (Fig 3I). To a lesser extent, this was also true for miR‐148a‐3p‐regulated genes, while no overlap of the eQTL data was found for genes regulated by miR‐181a‐5p, which is in line with the data linking miR‐148a‐3p and specifically miR‐181a‐5p to neuronal processes (Fig 3I). We also compared our transcriptomic findings to gene expression datasets from CK‐p25 mice, a mouse model for AD‐like neurodegeneration (Fischer *et al*, 2005) as well as gene expression and proteome data from human AD patients. We observed that the genes and proteins downregulated in AD patients strongly overlapped with the downregulated genes observed in response to miR‐148a‐3p and especially miR‐181a‐5p (Fig 3I). In conclusion, these data further confirm the hypothesis that increased levels of the 3 microRNAs reflect key processes important to neuronal and synaptic integrity that are known to be deregulated in cognitive diseases.

**Figure 4:**
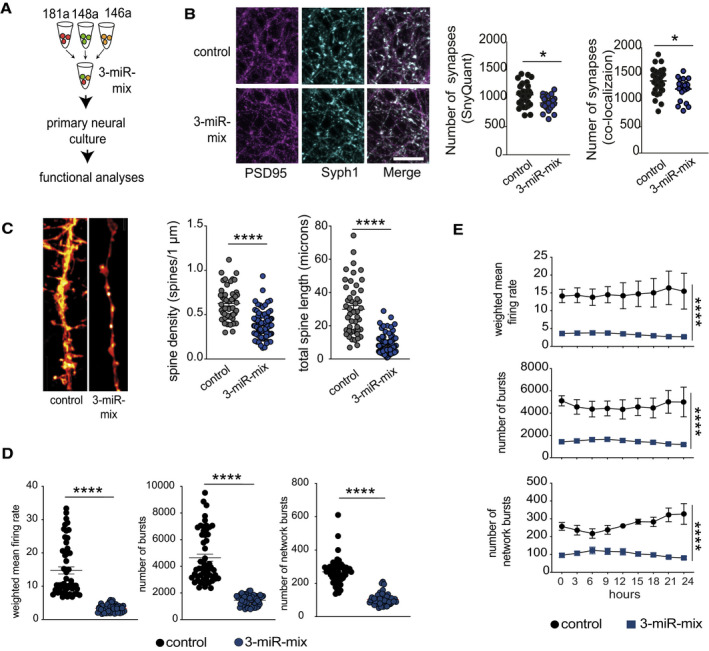
Increased expression of 3‐microRNA signature perturbs synaptic organization and neuronal activity Primary hippocampal neurons were treated with a mixture of 3‐miR mimic or control oligonucleotides, and follow‐up analyses (imaging, electrical recordings) were performed.Functional mature synapses were quantified via co‐localizations of pre‐ (synaptophysin 1) and the postsynaptic (PSD‐95) markers and compared between 3‐miR‐mix and control groups. Scale bar: 10 μm. Two independent methods (SynQuant and Colocalization) were used for quantification. 3‐miR‐mix reduced the number of functional synapses compared with controls (*n* = 24–30 images)Dendrite labeling and quantification. Dendritic spines were stained with Dil. Scale bar: 10 μm. Spine density and total spine length are substantially reduced in 3‐miR‐mix‐treated primary neurons compared to those treated with scrambled RNA (*n* = 49–97 images)Hippocampal neurons were cultured in a multielectrode array (MEA) plate equipped with sixteen electrodes. Spontaneous activity of the neurons was recorded at every 3 h (10 min/session) for 24 h. Weighted mean firing rate, number of bursts, and network bursts are significantly decreased in neurons treated with 3‐miR‐mix compared with control.The aberrant neuronal firing activity (weighted mean firing rate) and reduced number of bursts and network bursts were observed across the 24 h of time period. Data information: For panels B, C, D, E, following statistical test has been applied: Unpaired *t*‐tests, two‐tailed. Bars and error bars in these plots indicate mean ± SEM. *P < 0.05, ****P < 0.0001

**Figure EV3:**
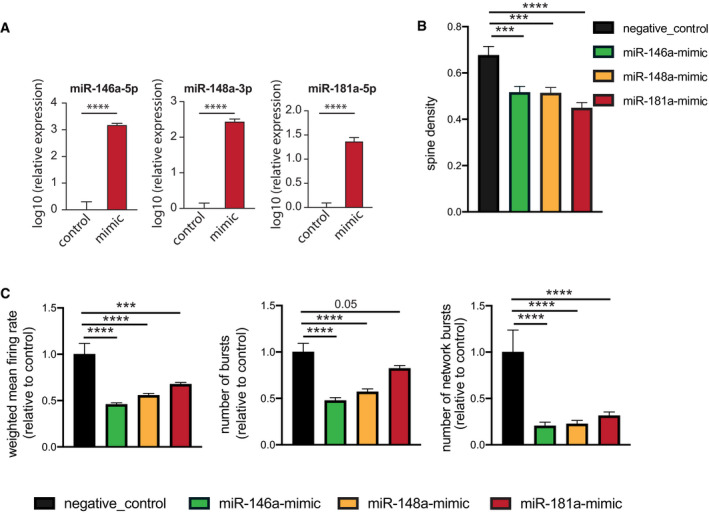
Effect of individual microRNAs on neuronal functions 3‐miR‐mix containing (miR‐146a‐5p, miR‐181a‐5p, miR‐148a‐3p mimics) was applied on primary hippocampal neurons at DIV10, and its effect was compared to neurons treated with a scrambled control RNA. After 48 h, cells were prepared for RNA Isolation. qPCR data from isolated RNA reveal increased expression of miR‐146a‐5p (left), miR‐148a‐3p (middle), and miR‐181a‐5p (right). Unpaired *t*‐test, two‐tailed, *n* = 5/group. ****P < 0.0001. Data are normalized to control and log10 scaled. Error bar indicates mean ± SEM.Primary hippocampal neurons were transfected with scrambled/individual microRNA mimics at DIV7, and dendritic spines were stained with Dil at DIV10 for dendrite labeling and quantification. Spine density is substantially reduced for mimic‐treated primary neurons compared to those treated with scrambled RNA. Barplots showing spine density among groups. *N* = 20 dendritic segments/group, one‐way ANOVA, Dunnett's multiple comparisons test, ***P < 0.001, ****P < 0.0001.Hippocampal neurons were cultured in a multielectrode array (MEA) plate equipped with sixteen electrodes. Individual microRNA mimic was applied at DIV7, and a downstream neuronal activity was measured at DIV10 and compared to scrambled control‐treated neurons. Weighted mean firing rate, number of bursts, number of network bursts. 6 replicates/group recorded 8× in every 3 h, each record lasting for 10 min. *N* = 48, one‐way ANOVA, Dunnett's multiple comparisons test, ***P < 0.001, ****P < 0.0001. Error bars indicate mean ± SEM.

To provide further evidence for this interpretation, we decided to directly analyze the role of the 3‐microRNA signature in synaptic organization and plasticity. Primary hippocampal mouse cultures that contain neuronal and glia cells were treated with a mixture of mimic oligonucleotides representing the 3‐microRNA signature (3‐miR‐mix). (Fig 4A). This treatment led to a significant increase in individual miR levels (Fig EV3). We first analyzed the number of synapses via STED microscopy to detect colocalization of the pre‐ and postsynaptic marker proteins synaptophysin 1 (Syph1) and postsynaptic density protein 95 (PSD‐95). Delivery of the 3‐miR‐mix reduced the number of synapses using 2 independent quantification methods (Fig 4B). In line with this observation, administration of the 3‐miR‐mix led to a significant reduction in the number of dendritic spines (Fig 4C). A similar reduction in dendritic spines and neuronal network activity was observed when the microRNAs were individually overexpressed (Fig EV3B and C). Next, we decided to test whether the observed structural alterations would translate into altered neuronal network plasticity. To this end, primary hippocampal cultures were grown on microelectrode array (MEA) plates to measure spontaneous extracellular potentials. Cultures were treated with the 3‐miR‐mix or scrambled RNA, and spontaneous activity was recorded for 24h (every 3 h for 10 min). Administration of 3‐miR‐mix led to aberrant neuronal activity. Namely, the mean firing rate, number of bursts, and network bursts were all severely impaired (Fig 4D), an effect that was observed across the entire 24h of recoding (Fig 4E). Taken together, these data show that increased expression of the 3‐microRNA signature impairs neural plasticity and provides further evidence that the analysis of this signature in blood might inform about mechanisms linked to cognitive function.

### A 3‐microRNA signature informs about cognitive status

**Figure EV4:**
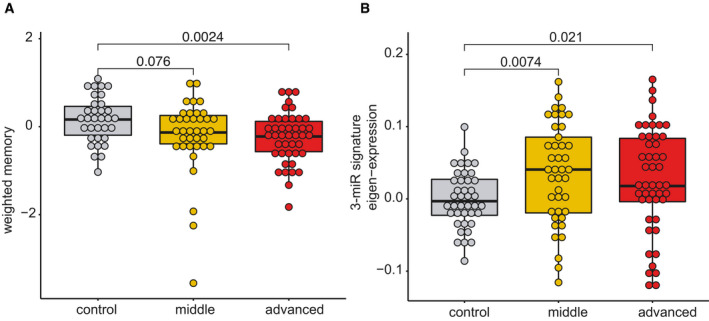
The 3 microRNA signature changes in aging humans that exhibit cognitive decline Although care has to be taken when comparing data from mice to humans, previous work suggests that 12‐month‐old mice are comparable to 40‐year‐old humans and that 1.5 months in a mouse’s lifetime approximately reflect 5 years of lifetime in humans (Dutta, 2016). Thus, our longitudinal experiment in mice (see Figs 2 and 3A and B) could be compared best to humans from 40 to 55 years of age. This is in line with previous cross‐sectional and longitudinal studies in humans, reporting that impairments of certain cognitive abilities become evident from 40 years of age while after 54 years of age, most cognitive domains significantly decline (Schaie, 1993; Park *et al*, 2002; Hedden & Gabrieli, 2004; Park & Reuter‐Lorenz, 2009; Salthouse, 2009; Singh‐Manoux *et al*, 2012). Therefore, we recruited healthy individuals aged between 30 and 77 years of age that were subjected to blood collection and the same cognitive phenotyping as in our discovery cohort (See Fig 1). Based on previous data (Schaie, 1993; Park *et al*, 2002; Hedden & Gabrieli, 2004; Park & Reuter‐Lorenz, 2009; Salthouse, 2009; Singh‐Manoux *et al*, 2012), we divided the individuals into a “control group” that is expected to exhibit full cognitive functioning (30–40 years of age), a “middle‐age group” (41–53 years of age) that is expected to show very mild signs of cognitive decline, and an “advanced age group” (54–77 years of age) that are expected to display significant cognitive decline. Despite the fact that older individuals exhibit cognitive impairments, we like to reiterate that all of these individuals were healthy and none of them suffered from mild cognitive impairment or dementia. We confirmed a non‐significant trend for reduced cognition in the “middle‐age group” that became significant in the “advanced age group”.Next, we performed small RNA sequencing from all collected samples and analyzed the microRNA expression. Similar to the data obtained in mice, co‐expression of the 3‐miR signature was significantly increased already in the “middle‐age group” and plateaued in “advanced age group” when compared to individuals 30–40 years of age. Although these data are cross‐sectional, they are in line with our observation from the longitudinal study in mice and suggest that also in humans, expression of the 3‐microRNA signature might increase in blood prior to the detection of significant cognitive impairment, at least in the employed experimental design. Kruskal–Wallis test, *n* = 40–47 human subjects.

**Figure 5:**
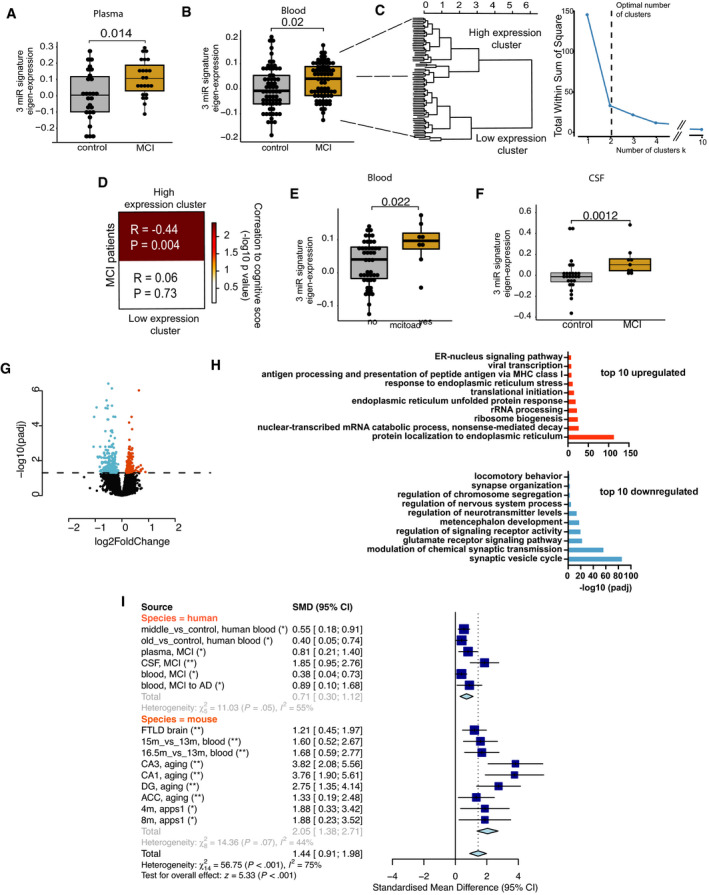
Expression of 3‐microRNA signature in human patients A–FAll data have been adjusted for age, gender, and other latent covariates for downstream eigenvalue calculation. (A) Eigenvalue showing the increased expression of the 3‐microRNA signature in blood plasma samples of age‐matched MCI (*n* = 23) patients compared with controls (*n* = 27). (B) Expression of 3‐microRNA signature is increased in PAXgene blood samples of MCI patients (*n* = 71) compared with controls (*n* = 65) from the DELCODE cohort. (C) Clustering of eigen expression identifies two expression clusters in MCI patients based on elbow method of detecting optimum number of clusters. (D) Patients representing the cluster with higher expression of 3‐miR eigenvalue show negative correlation with their weighted cognitive score (cor = 0.444, *P* = 0.004). In contrast, patients with low expression of 3‐miR signature did not show significant correlation with the cognitive score (*P* = 0.73). (E) Eigenvalue showing the expression of the 3‐microRNA signature in MCI patients for those the follow‐up diagnostic data assessed 2 years after was available. 15% of these MCI patients developed Alzheimer´s disease (AD), while the rest 85% patients remained with MCI (stable MCI). The boxplot depicts the increased expression levels of 3‐miR signature in patients who converted to AD (*n* = 8) compared with those that had stable MCI (*n* = 47). (F) Increased expression of 3‐microRNA signature in cerebrospinal fluid (CSF) of MCI patients (*n* = 9) compared with controls (*n* = 26). Wilcoxon rank test, *P*‐value is given on the corresponding panel. In the boxplots in (A, B, E, F), the centerline indicates the median, while the upper and lower lines represent the 75th and 25th percentiles, respectively. The whiskers represent the smallest and largest values in the 1.5× interquartile range.GHuman bioengineered neuronal organoids (BENOs) were treated at DIV 60 with the *3*‐miR‐mix or corresponding controls for 24 h, and RNA‐seq was performed from prepared RNA. Volcano plot displays the significant deregulated genes in BENOs after over‐expressing the 3‐miR‐mix (FDR < 0.05).HGene ontology shows top 10 significant up‐ and downregulated processes based on the differentially expressed genes. X‐axis represents the ‐log10 of adjusted *P*‐value.IA meta‐analysis for 3‐microRNA signature was performed across different datasets. The upper part shows the human datasets, while the lower part shows the investigated mouse datasets. In addition to the datasets presented in our study, we also employed cortical small RNAome data (GSE8998) from a fronto‐temporal dementia (FTLD) mouse model at a presymptomatic state (Swarup *et al*, 2018). Standardized mean difference (SMD) of zero indicates no effect. Deviation from zero would indicate either an increase or a decrease in the eigen expression for the 3‐microRNA signature. Asterisks represent the adjusted *P*‐value across studies (length = 15). *P < 0.05, **P < 0.01. Standardized mean difference (SMD) of 3‐microRNA signature is given along with the corresponding lower and upper intervals. A large pooled standardized mean difference (1.44) for 3‐microRNA signature was observed across species, and the overall effect in both species (Z = 5.33) was highly significant (*P* < 0.001).

**Figure EV5:**
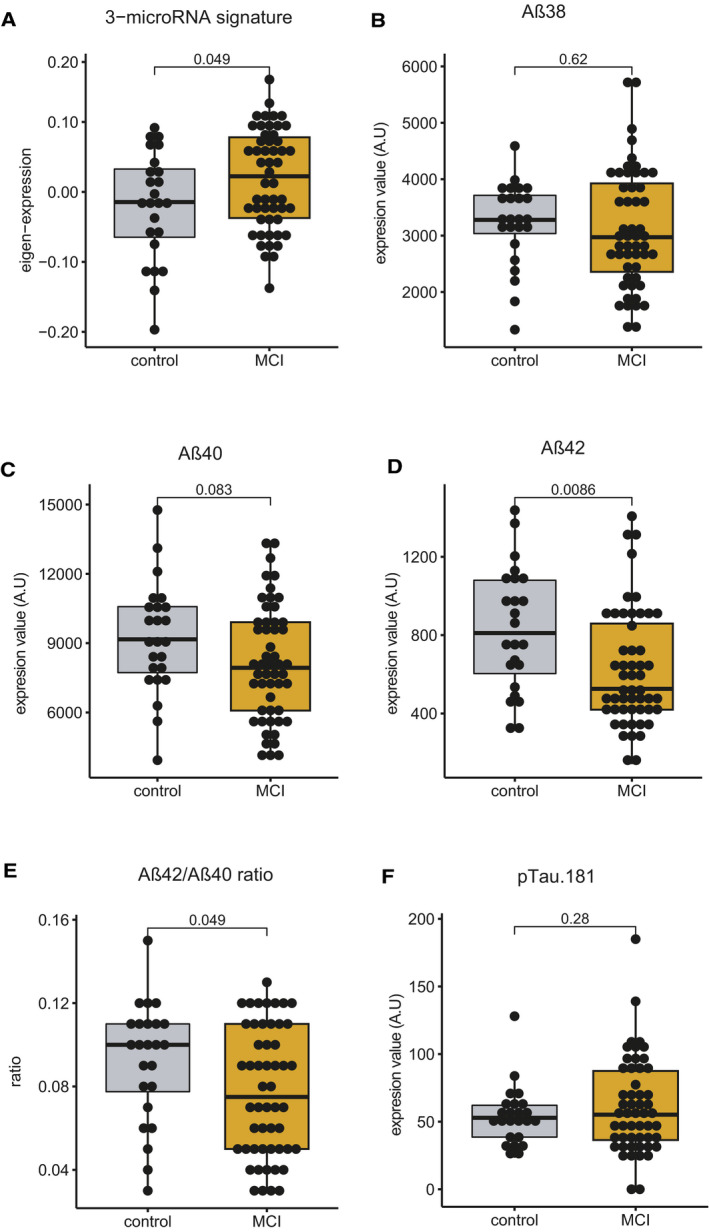
Comparison of the 3‐microRNA signature in blood with established CSF AD biomarker within the same control and MCI patients Please note that expression data of the CSF biomarker were not available for all the samples used in Fig 5B. Therefore, the comparative analysis of the blood 3‐microRNA signature and the CSF biomarker was performed on control and MCI patients for which all datasets were available; control = 24, MCI = 52. A–EEigenvalue of 3‐microRNA signature in blood is significantly increased in MCI patients compared with controls. Levels of (B) Aβ38 and (C) Aβ40 did not change significantly between patients and control subjects. Both (D) Aβ42 alone and (E) Aβ42/40 ratio were significantly different between control and MCI patients.FThere was no statistically significant difference in the levels of phosphorylated Tau 181 when comparing MCI patient versus controls. Since Tau could be considered a marker for neurodegeneration, it is not unexpected that changes in Aβ precede changes in Tau pathology. Unpaired *t*‐tests, two‐tailed. These data suggest that the 3‐microRNA signature is comparable to more invasive established AD biomarker.

The above‐described findings encouraged us to test whether the 3‐microRNA signature could help to detect alterations in cognitive function in humans. In a first approach, we analyzed the expression of the 3‐microRNA signature in individuals of different age‐groups in a cross‐sectional setting. Similar to the data obtained in mice, we find evidence that the signature might increase in blood prior to the detection of significant cognitive impairment (Fig EV4; Dataset EV11). Encouraged by these findings obtained from healthy humans, we decided to further test the performance of the 3‐microRNA signature in cognitive diseases. First, we analyzed a previously published small RNA dataset (Kayano *et al*, 2016) obtained from plasma samples that were collected from patients suffering from mild cognitive impairment (MCI). We like to mention that this dataset was generated via qPCR array technology that is biased by probe design and has a different dynamic range when compared to sequencing‐based approaches. Since the 3 microRNAs of our signature were detectable in this dataset, we decided to conduct the analysis. The 3‐microRNA signature was significantly increased in MCI patients when compared to age‐matched healthy individuals (Fig 5A). To confirm this observation, we performed small RNA sequencing from blood samples (total blood collected via PAXgene tubes) obtained from control individuals and MCI patients of the DELCODE study (Jessen *et al*, 2018; Dataset EV12). No significant variability in cognitive function between male and female was observed in DELCODE cohort (*P* = 0.86) (Dataset EV12). We found that the expression of the 3‐microRNA signature was significantly increased in MCI patients (Fig 5B). Please note that we removed three samples from the dataset after automatic detection of outliers based on low‐quality *Z*‐score. It turned out that these were 3 MCI patients with 2.5 standard deviations below the average expression of the 3‐microRNA signature, and it is worth mentioned that these individuals did not convert from MCI to AD when reanalyzed 2 years later. Interestingly, data on the CSF levels of Aβ42/40 and phospho‐Tau were available for most of the analyzed control and MCI patients of the DELCODE study. When we compared the ability to distinguish the same control individuals from MCI patients via our 3‐microRNA signature in blood to the analysis of Aβ42/40 ratio measured in CSF, the 3‐microRNA signature performed equally good, while levels of phospho‐Tau did not yet reveal significant changes (Fig EV5). Although the 3‐microRNA signature significantly differed among controls and MCI patients, the expression was rather variable at the individual level (Fig 5A and B). Therefore, we subjected the data to an unbiased hierarchical clustering analysis and observed two main clusters within the MCI patients, representing patients with either low (low expression cluster) or high expression level (high expression cluster) of the 3‐microRNA signature (Fig 5C). On the basis of our mouse experiments, it is tempting to speculate that also in humans, individuals with high blood levels of the 3‐microRNA signature might be more likely to undergo further cognitive decline. In line with this hypothesis, we observed that specifically those MCI patients that were part of the “high expression cluster” showed a significant negative correlation of the 3‐microRNA signature to cognitive function (Fig 5D). These data further support the hypothesis that high circulating levels of the 3‐microRNA signature may indicate a low cognitive reserve and higher risk for cognitive decline. We were able to directly test this hypothesis—at least in part—in MCI patients of the DELCODE study, since follow‐up phenotypic data were available for some—but not for all—of the individuals (*n* = 53). Thus, we asked whether high levels of the 3‐microRNA signature would be associated with the future conversion from MCI to AD. We were able to compare the expression level of the 3‐microRNA signature in MCI patients that converted from MCI to AD within 2 years after blood collection (*n* = 8) to MCI patients that did not progress to AD within the same time period (*n* = 45). Although we cannot exclude that some MCI patients would convert from MCI to AD at a later time point, our analysis revealed that the expression of the 3‐microRNA signature was significantly higher in MCI patients that converted to AD 2 years after blood collection, when compared to those characterized by stable MCI diagnosis (Fig 5E). Since our animal experiments suggest that increased blood levels of the 3‐microRNA signature are paralleled by corresponding changes in the brain, we asked whether the signature would be also increased in the brains of humans suffering from MCI. Since the analysis of post‐mortem human brain tissue is often confounded by post‐mortem delay, RNA quality, and other factors, we decided to analyze data from cerebrospinal fluid (CSF) of living MCI patients. Thus, we compared data from probands that did not suffer from cognitive diseases and age‐matched individuals diagnosed with mild cognitive impairment (MCI) as a proxy for the expression of microRNAs in the brain. The analysis of corresponding small RNA sequencing data revealed that the expression of the 3‐microRNA signature was significantly increased in MCI individuals (Fig 5F). Considering the mechanistic studies we performed in mice, these data suggest that altered levels of the 3‐microRNA signature control cellular processes essential for cognitive function also in the human brain. To test this hypothesis more directly, we employed human bioengineered neuronal organoids (BENOs) (Zafeiriou *et al*, 2020) that were treated with the 3‐miR‐mix followed by RNA sequencing. In line with the data obtained in mouse cultures, we observed that elevated levels of the 3‐microRNA signature induced gene expression changes and related biological processes linked to cellular stress, while genes representing synaptic function‐related processes were decreased (Fig 5G and  H).

Taken together, our findings suggest that the 3‐microRNA signature could be suitable molecular marker to inform about cognitive status and reserve and help to detect individuals at risk to develop dementia. In fact, the 3‐microRNA signature was consistently deregulated in a meta‐analysis performed on 15 different human and mouse datasets and revealed a highly significant pooled effect (*z* = 5.33, *P* < 0.001) and standardized mean difference of 1.44 [lower interval: 0.91, upper interval: 1.98] (Fig 5I). The 3‐microRNA signature outperformed an analysis conducted with all 7 microRNAs initially identified after feature selection (Appendix Fig S7, see also Fig 2), as well as single or combinations of two microRNAs of the 3‐microRNA signature, further supporting the specificity of the signature (Appendix Fig S7). We also analyzed a set of 1000 random 3‐microRNA combinations selected from the 55 aging responsive microRNAs that were initially used for feature selection (see Fig 2E). Again, the 3‐microRNA signature outperformed (*Z*‐score: 5.25, adjusted *P*‐value: 0.0.0001 [method = “BH”, *n* = 1,000], standardized mean difference 1.71 [0.64–2.78], significant in all datasets tested) all 1,000 random combinations (Dataset EV13). In line with this result, a random set of 3 microRNAs (1,000 random combinations were tested) selected from the human microRNAome of healthy individuals from PsyCourse cohort (See Fig 1) (overall effect, statistical significance after multiple adjustments, number of datasets to be deregulated) than the experimentally curated 3‐microRNA signature reported in this study (Dataset EV14). We also analyzed a previously reported blood‐based 12‐microRNA signature that was detected by comparing AD patients to healthy controls (Leidinger *et al*, 2013). This signature was deregulated in CSF from MCI patients (Appendix Fig S8A) but it was not consistently regulated across the datasets employed in the meta‐analysis (Appendix Fig S8). These data support our initial hypothesis that circulating microRNA signatures that were identified to reliably distinguish AD patients from controls might not be suitable for the early detection of individuals at risk for cognitive decline. Moreover, when we tested three microRNAs from a previously described microRNA‐piRNA signature that was observed in CSF exosomes from MCI patients (Jain *et al,* 2019; Appendix Fig S8B), we observed similar outperformance for the 3‐microRNA signature described in the current study.

### The three‐microRNA signature is a target for RNA therapeutics in dementia

**Figure 6:**
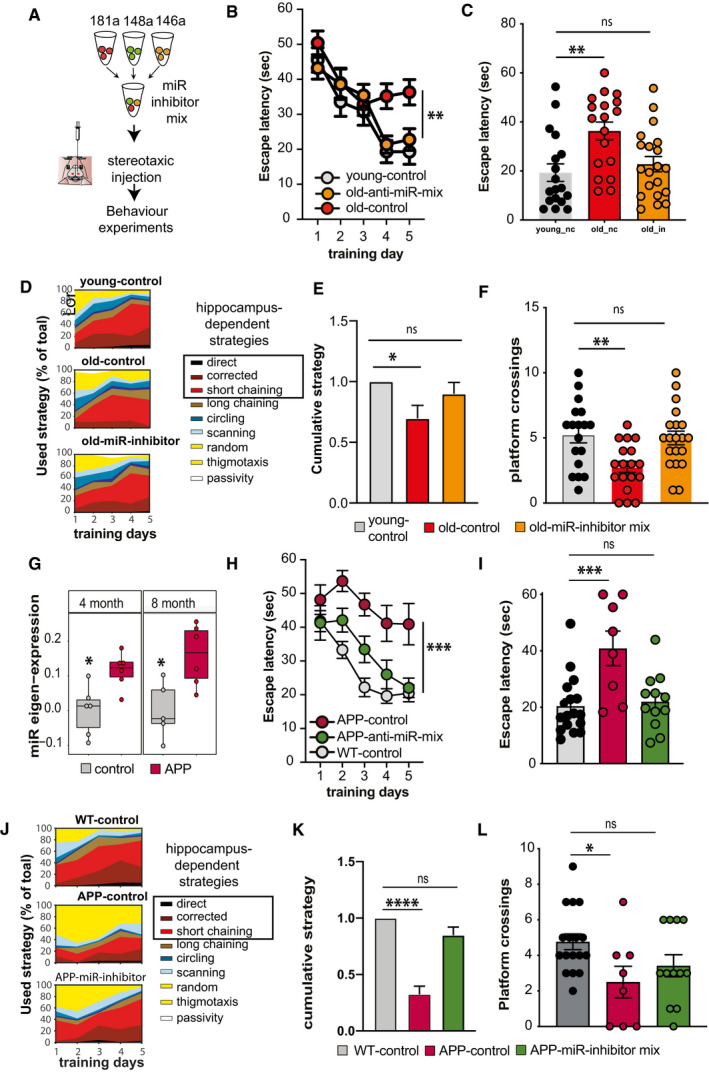
Targeting the 3‐microRNA signature reinstates cognitive function in mouse models for dementia Experimental design. Inhibitor mixture for 3 microRNAs (anti‐miR‐mix), namely miR‐181a‐5p, miR‐148‐3p, and miR‐146a‐5p was injected into the hippocampal CA of male wild‐type mice prior to behavioral testing. As control, scrambled siRNAs were injected as described above. Experiments were performed in two mouse models representing aging and Alzheimer´s disease (AD).Escape latency during the water maze training comparing 3‐month‐old (young‐control, *n* = 18) mice and 16,5‐month‐old (old‐control, *n* = 18) mice injected with scrambled control oligonucleotides and 16,5‐month‐old mice injected with microRNA inhibitors (old miR‐inhibitor mix, *n* = 20). Bars and error bars indicate mean ± SEM.Escape latency on final day of water maze training was impaired improved in old mice treated with the microRNA inhibitors mix. Young‐control (*n* = 18, 19.3 ± 15.23, mean ± SD); old‐control (*n* = 18, 36.31 ± 15.38, mean ± SD); old‐inhibitor (*n* = 20, 22.78 ± 13.85, mean ± SD).Depiction of the search strategies during the water maze training.The cumulative cognitive score calculated for each day on the basis of hippocampal‐dependent strategies was significantly impaired when comparing mice old‐control mice to young‐control mice. Data are normalized to young‐control group. Bars and error bars indicate mean ± SEM. Number of mice: young‐control, *n* = 18; old‐control, *n* = 18; old miR‐inhibitor mix, *n* = 20.Number of visits to the platform during the probe test. young‐control (5.2 ± 2.5, mean ± SD); old‐control (2.7 ± 1.83, mean ± SD); old‐inhibitor (5.0 ± 2.3, mean ± SD). All mice were male. Number of mice: young‐control, *n* = 18; old‐control, *n* = 18; old miR‐inhibitor mix, *n* = 20.Eigenvalue showing the expression of the 3‐microRNA signature in the hippocampus of 4‐ and 8‐month‐old APPPS1‐21 mice. The centerline indicates the median, while the upper and lower lines represent the 75th and 25th percentiles, respectively. The whiskers represent the smallest and largest values in the 1.5× interquartile range. Number of mice: 5–6/group.Escape latency during the water maze training comparing 7‐month‐old wild‐type mice (WT control, *n* = 17, male: 9, female: 8) and APPPS‐21 mice (APP‐control, *n* = 8, male: 6, female: 2) injected with scrambled control oligonucleotides and APPPS1‐21 mice injected with microRNA inhibitors (APP miR‐inhibitor mix, *n* = 12, male: 8, female: 4). Bars and error bars indicate mean ± SEM.Escape latency measured on the last day of water maze training was reduced in APP‐control mice. However, learning performance was rescued in APP miR‐inhibitor mix mice. WT control: 20.43 ± 10.33 (mean ± SD), APP‐control: 40.92 ± 17.33 (mean ± SD), and APP miR‐inhibitor mix: 22.04 ± 9.96 (mean ± SD). Sex did not affect the data. Number of mice: WT control, *n* = 17, male: 9, female: 8; APP‐control, *n* = 8, male: 6, female: 2; APP miR‐inhibitor mix, *n* = 12, male: 8, female: 4.Depiction of the search strategies during the water maze training in experimental groups.The cumulative cognitive score calculated for each day on the basis of hippocampal‐dependent strategies was significantly impaired when comparing WT control mice to APP‐control mice. Data are normalized to WT control group. Bars and error bars indicate mean ± SEM. Number of mice: WT control, *n* = 17, male: 9, female: 8; APP‐control, *n* = 8, male: 6, female: 2; APP miR‐inhibitor mix, *n* = 12, male: 8, female: 4.Comparison of the number of visits to the platform during probe test. WT control: 4.76 ± 1.82 (mean ± SD), APP‐control: 2.5 ± 2.5 (mean ± SD), and APP miR‐inhibitor mix: 3.41 ± 2.15 (mean ± SD). Bars and error bars indicate mean ± SEM. Number of mice: WT control, *n* = 17, male: 9, female: 8; APP‐control, *n* = 8, male: 6, female: 2; APP miR‐inhibitor mix, *n* = 12, male: 8, female: 4. Data information: (B, H) Mixed‐effects analysis followed by Tukey’s multiple comparison test. (C, E, F, I, K, L) One‐way ANOVA followed by Dunnett´s multiple comparisons test. (G) Unpaired *t*‐tests, two‐tailed. *P < 0.05, **P < 0.01, ***P < 0.001, ****P < 0.0001.

Our finding that the 3‐microRNA signature is increased not only in blood but also in the brain of cognitively impaired mice and in CSF of MCI patients suggests that targeting this signature in the CNS might be a suitable approach for RNA therapeutics. Our data showed that the 3‐microRNA signature is increased in 3 different hippocampal sub‐regions of aged mice as well as in the ACC (see Fig 3D, Appendix Fig S8). Previous data support the view that the hippocampus is a brain region affected early in humans that develop age‐associated memory impairment (Wolf *et al*, 2001; Dicks *et al*, 2019). Furthermore, hippocampus‐dependent age‐associated memory decline can be measured in mice (Peleg *et al*, 2010). Therefore, we decided to study and target the hippocampus as a first approach. An inhibitor mix (anti‐miR‐mix) containing inhibitory oligonucleotides against miR‐181a‐5p, miR‐146a‐5p, and miR‐148a‐3p led to decreased level of microRNAs both *in vitro* (Appendix Fig S9A and B) and *in vivo* when injected into the hippocampus of mice (Appendix Fig S9C and D). Thus, we used lipid nanoparticles containing the anti‐miR‐mix and administered these particles to the hippocampal CA region of 16.5‐month‐old mice (anti‐miR‐mix group). Mice of the same age (old‐control group) and 3‐month‐old young mice (young‐control group) that were injected with corresponding scrambled oligonucleotides served as control (Fig 6A). Five days post‐injection, mice were subjected to the water maze training to test spatial reference memory. Mice of the old‐control group displayed a significantly increased escape latency, when compared to the young‐control group indicating impaired spatial reference memory (Fig 6B and C). Notably, old mice injected with the anti‐miR‐mix performed comparable to young mice, suggesting improved hippocampus‐dependent memory function (Fig 6B and C). Of note, in an independent experiment, young mice injected with the anti‐miR‐mix did not show difference in performance when compared to a control group (Appendix Fig S10). We analyzed the search strategies during the training procedure in greater detail and observed that mice of the old‐control group less efficiently adapted hippocampus‐dependent search strategies when compared to young mice and old mice treated with the anti‐miR‐mix (Fig 6D). This effect was significant, when we analyzed the search strategies by calculating a cumulative cognitive score on the 5 days of training (Fig 6E). During a probe test, mice of the old‐control group displayed a reduced number of platform crossings when compared to the young‐control group, suggesting impaired memory retrieval (Fig 6F). In contrast, old mice injected with the anti‐miR‐mix performed similar to young mice (Fig 6F). qPCR analysis performed at the end of the experiment confirmed the persisted downregulation of three microRNAs in the hippocampal CA region when the anti‐miR‐mix was injected. Expression in the corresponding hippocampal dentate gyrus was not affected, indicating specificity of the injection procedure (Appendix Fig S11A). These data suggest that an RNA‐based therapeutic approach targeted toward the 3‐microRNA signature can improve memory function in cognitively impaired old mice. Considering that the 3‐microRNA signature was also increased in MCI patients, we decided to test its therapeutic potential also in a disease model for AD. We employed APPPS1 mice, a well‐established model for amyloid deposition that displays hippocampus‐dependent memory impairment at 6–8 months of age (Radde *et al*, 2006; Agís‐Balboa *et al*, 2017; Martinez‐Hernandez *et al*, 2017). Similar to the experiments outlined in aged mice, we reasoned that targeting the hippocampus would be a suitable first approach. We performed small RNA sequencing of hippocampal tissue obtained from APPPS1 mice at 4 and 8 months of age, representing time points before and after the onset of detectable memory impairment. Our data reveal that the 3‐microRNA signature is significantly increased already at 4 and also at 8 months of age when comparing APPPS1 to age‐matched control mice (Fig 6G). Encouraged by this observation, we decided to test whether administration of the anti‐miR‐mix could ameliorate memory impairment in APPPS1 mice. We decided to employ 7‐month‐old APPPS1 mice and injected the anti‐miR‐mix into the hippocampal CA (APP anti‐miR‐mix group). As control groups, we injected age‐matched APPPS1 (APP‐control group) and wild‐type mice (WT control group) with a mix of corresponding scrambled oligomers. When we subjected mice to the water maze training, the anti‐miR‐mix group was able to significantly improve the escape latency in APPPS1 mice (Fig 6H and I), suggesting that the anti‐miR‐mix ameliorates memory impairment in a mouse model for AD. This effect was also obvious when we analyzed in detail the different search strategies. Thus, APP mice failed to adapt hippocampus‐dependent learning strategies, while APP mice treated with the anti‐miR‐mix displayed an increase in such strategies similar to wild‐type control mice (Fig 6J). This effect was also highly significant when we analyzed the cumulative score of the search strategies at the 5 days of training (Fig 6K). Furthermore, improved memory retrieval during the probe test was observed in anti‐miR‐mix‐treated APP mice (Fig 6L). Similar to the data obtained in aging mice, qPCR analysis performed at the end of the experiment confirmed the downregulation of three microRNAs in the injected CA region, while the dentate gyrus was not affected (Appendix Fig S11B). In conclusion, these data support the view that targeting the 3‐microRNA signature could be a suitable strategy for a biomarker‐guided RNA therapy toward dementia. It is, however, interesting to mention that although we observed that increasing the levels of the 3‐microRNA signature appears to be detrimental to neural function and that targeting all 3 microRNAs using anti‐miRs can ameliorate cognitive decline in model systems, miR‐148a‐3p was initially observed within the brown co‐expression module that was positively correlated to cognition in young healthy humans (see Fig 1). Thus, further research on the specific function of this microRNA in the CNS across lifespan in humans and mammalian model systems is needed to fully appreciate its role in learning and memory function.

### Targeting the 3‐microRNA signature partially reinstates transcriptional homeostasis in disease models

**Figure 7:**
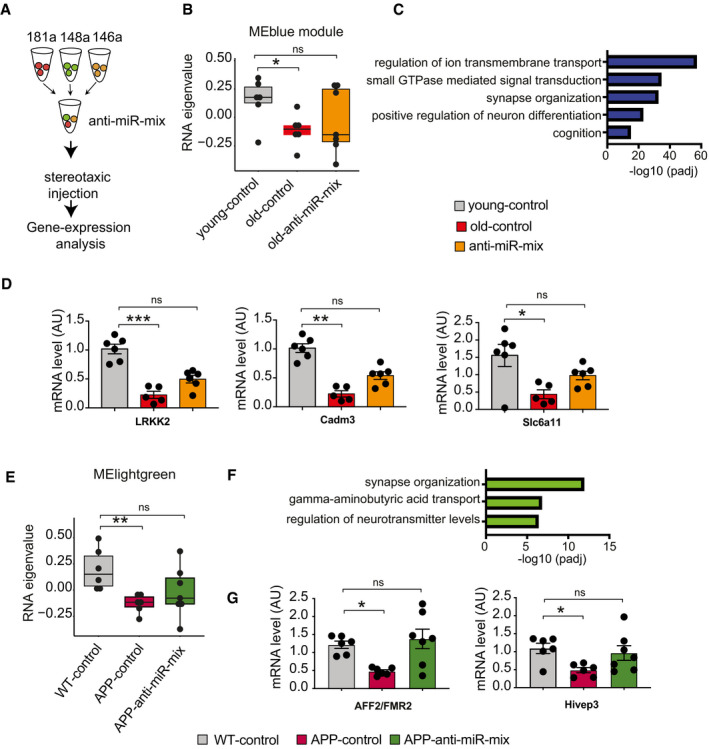
The 3‐microRNA signature is a target for RNA therapeutics to treat neuronal dysfunction Experimental outline. Anti‐miR‐mix of the 3 microRNAs was injected into the dorsal hippocampus of the mice as previously described. RNA‐seq data were generated from dorsal hippocampal tissues and compared to those treated with control scrambled oligonucleotides.Weighted gene co‐expression analysis of hippocampal RNA‐seq data identified the MEblue gene cluster that is decreased when comparing 3‐month‐old mice (young‐control) to cognitively impaired 16.5‐month‐old mice (old‐control) with a scramble control oligonucleotide injected. Treating old mice with the miR‐inhibitor mix (old miR‐inhibitor mix) reinstated gene expression of this cluster, at least in part (*n* = 6–7, Kruskal–Wallis test).Gene ontology reveals that the MEblue cluster is linked to cognition and synapse organization.qPCR assay for several synaptic genes (LRKK2, Cadm3, and Slc6a11) confirms reinstatement of gene expression with anti‐miR‐mix (*n* = 5–7, Kruskal–Wallis test).Weighted gene co‐expression analysis of hippocampal RNA‐seq data identified a MElightgreen gene cluster that is decreased when comparing wild‐type control (WT control) to APPPS1‐21 mice (APP‐control) and was reinstated in APPPS1‐21 mice‐treated miR‐inhibitor mix (*n* = 6–7, Kruskal–Wallis test).Gene ontology reveals that the MElightgreen cluster is linked to cognition and synapse organization.qPCR data show rescue of AFF2/FMR2 and Hivep3 expression in APP/PS1 mice treated with inhibitor cocktail (*n* = 6–7, Kruskal–Wallis test). Data information: *P < 0.05, **P < 0.01, ***P < 0.001. Bars and error bars indicate mean ± SEM. In boxplots (B, E), the centerline indicates the median, while the upper and lower lines represent the 75^th^ and 25^th^ percentiles, respectively. The whiskers represent the smallest and largest values in the 1.5× interquartile range.

While microRNAs control cellular homeostasis at the level of transcriptional networks, aberrant gene expression is key hallmark of cognitive diseases including AD (Fischer, 2014b). Thus, we hypothesized that reinstatement of memory function in aged and in APPPS1 mice might—at least in part—be due to the action of the anti‐miR‐mix on hippocampal gene expression. To test this, we performed RNA sequencing from hippocampal tissue of aged and APPPS1‐21 mice that received either control RNA or the anti‐miR‐mix (Fig 7A). Wild‐type littermates treated with control RNA were used as additional control. First we analyzed the data from the experiment employing aged mice and performed Weighted Gene Co‐expression Analysis (Langfelder & Horvath, 2008). We identified 29 different co‐expression modules in the entire RNA‐seq dataset of which 4 represent neuronal cluster (Appendix Fig S12A). While three of these modules were unaffected among groups (Appendix Fig S12B), the MEblue module paralleled our behavioral findings and was significantly deregulated when comparing the young‐control to the old‐control group, whereas its expression was partially reinstated to the level of the young‐control group in response to anti‐miR‐mix treatment (Fig 7B). Gene ontology analysis revealed that the genes of the Meblue module represent processes linked to synapse organization and cognition (Fig 7C, Dataset EV15). We confirmed the expression of three representative genes, namely LRKK2, Cadm3, and Slc6a11 (Fig 7D). In the RNA sequencing data obtained from APPPS1 mice, weighted Gene Co‐expression Analysis (Langfelder & Horvath, 2008) allowed us to detected 26 different co‐expression modules. Of these modules, MElightgreen and MEblue overlapped with neuronal gene set with high significance (Appendix Fig S12C). However, only the MElightgreen module was significantly deregulated when comparing the WT control group to the APP‐control group (Fig 7E, Appendix Fig S12D), while its expression was partially normalized to control levels in the APP anti‐miR‐mix group (Fig 7E). The genes within the MElightgreen module represent processes linked to synaptic function, similar to the genes for the MEblue module detected in aged mice (Fig 7F, Dataset EV15). Indeed, we confirmed the differential expression in control and APPPS1‐21 mice via qPCR for two representative genes, namely AFF2/FMR2 that encodes the fragile X mental retardation protein and Hivep3 which encodes the transcription factor kappa‐binding protein 1 (Fig 7G). Moreover, genes from MEblue (aging, see Fig 7B) and MElightgreen (APP, see Fig 7E) modules show 35% (Fisher´s exact test, ****P < 0.0001) and 38% (Fisher´s exact test, ****P < 0.0001) overlap to genes having 3’ UTR binding sites for miR‐146a‐5p, miR‐148a‐5p, or miR‐181a‐5p (Dataset EV16) and we have confirmed the regulation of selected candidate genes via a luciferase assay (Appendix Fig S13). In sum, these data suggest that targeting the 3‐microRNA signature can help to reinstate—at least in part—transcriptional homeostasis in 2 different animal models for cognitive decline.

## Discussion

We provide evidence that a circulating 3‐microRNA signature correlates with cognitive function, is linked to cognitive decline, that the 3 microRNAs regulate processes important for neuronal plasticity in the adult brain and that the signature serves as a target for RNA therapeutics. These data provide unprecedented evidence for the view that the blood microRNAome could be suitable as molecular biomarker for neuropsychiatric and neurodegenerative diseases (Rao *et al*, 2013; Galimberti *et al*, 2014; Hill & Lukiw, 2016; Kumar *et al*, 2017; Swarbrick *et al*, 2019; Roy *et al*, 2020). However, it is important to reiterate that our approach did not aim to identify microRNA biomarker that can distinguish patients suffering from a specific brain disease from controls. Rather, we employed an integrative approach starting with the analysis of cognitive variability in healthy humans and subsequently used multiple filtering steps with the aim to specifically identify microRNAs that could inform about the cognitive status and reserve and help to detect individuals at risk for pathological memory impairment. Our approach is therefore different to previous studies including data from our group (Jain *et al*, 2019) that were based on the comparison of microRNAs or other small non‐coding RNAs expressed in blood or CSF samples from patients and controls (Kumar *et al*, 2017). While the previous data are valid, we reasoned that the reported candidates may not be optimal for screening approaches with the aim to detect individuals at risk for developing cognitive decline. Although a single research paper cannot conclusively answer this issue, using our approach we eventually identified 3 microRNAs as a signature. These are miR‐181a‐5p, micro‐RNA148‐3p, and miR‐146a‐5p. The eigen expression of this signature increases in the blood of aging mice prior to cognitive decline, and we obtained evidence that it is also altered in aging humans prior to the detection of non‐pathological cognitive decline. While the data from mice represent a longitudinal study, the data from aging humans were based on a cross‐sectional analysis. Our experimental approach was designed to circumvent this issue, since corresponding data in humans are difficult to obtain. Nevertheless, longitudinal human data would eventually be necessary to further support our findings. It should also be mentioned that despite the obvious advantages provided by the combined analysis of human and mouse data in a feed‐forward feed‐backward approach, in such an experimental setting we will only detect microRNAs that are conserved between mice and humans. Moreover, care has to be taken when comparing neuropsychological assessment of cognitive function in humans to spatial reference memory measured in the water maze paradigm in mice. It is nevertheless tempting to speculate that aging humans with high circulating levels of the 3‐microRNA signature are more likely to develop future cognitive diseases. In line with this, the circulating 3‐microRNA signature also differed when comparing control to MCI patients and was also increased in MCI patients who converted to AD within the next 2 years. While it will be important to substantialize these data via further studies, our findings are supported by a number of previous observations. For example, increased circulating levels of miR‐181a‐5p were detected in age‐matched individuals with mild cognitive impairment when compared to healthy controls via qPCR analysis of plasma samples (Nagaraj *et al*, 2017). Another study reported elevated miR‐181a‐5p levels in a mouse model for AD (Rodriguez‐Ortiz *et al*, 2014), suggesting that miR‐181a‐5p levels generally increase in correlation with cognitive decline and would further increase in case of disease progression. Similar data have been reported for miR‐148a‐3p that was altered in serum samples of AD patients when compared to control individuals (Dong *et al*, 2015), while serum levels of miR‐146a‐5p were found to correlate with disease severity in AD patients (Maffioletti *et al*, 2020). Another recent study analyzed 9 microRNAs in human plasma samples via qPCR and found that miR‐146a and miR‐181a are increased in MCI patients that progress to AD within 2 year (Ansari *et al*, 2019). However, the available literature on microRNAs—including miR‐146a‐5p and miR‐181a‐5p—as biomarkers for AD is conflicting (Herrera‐Espejo *et al*, 2019). One reason might be that these studies analyzed single microRNAs and did not consider a signature based on—for example—the eigen expression. This view is supported by our analysis of the 3‐microRNA signature for its performance across 15 mouse and human datasets. While miR‐146a‐5p or the combination of miR‐146a‐5p and miR‐181a‐5p was also significantly altered in some datasets, the results were rather heterogeneous and only the combination of all 3 microRNAs of the signature yielded consistent data. This view is in line with our data, suggesting that the 3 microRNAs reflect multiple, yet inter‐dependent key processes linked to cognitive function and decline, such as neuronal plasticity and neuroinflammation. Hence, the importance of each individual microRNA may differ among individuals. Since the combined signature takes into account various inter‐linked patho‐mechanisms, it outperforms the analysis of single microRNAs or other combinations. In fact, the 3 microRNAs were deregulated in brain tissue of mouse models for cognitive diseases prior to disease onset, namely in models for AD, FTLD, and in human CSF samples from MCI patients. These data support the view that our signature does not reflect any specific neurodegenerative disease but informs about cognitive status and the risk to develop cognitive decline and eventually dementia. At the functional level, we were able to demonstrate that manipulating the expression levels of the 3 microRNAs in mouse neural cultures and in human brain organoids leads to pathological alterations related to cellular stress pathways, inflammation, synaptic plasticity, and neuronal network activity. We like to state that despite the obvious advantages in performing molecular studies, brain organoids and mice are model systems that cannot fully recapitulate cognitive decline in humans. Nevertheless, the data are in line with previous findings linking miR‐181a‐5p to neuronal function. For example, calorie restriction led to a downregulation of miR‐181a‐5p, which was linked to improved neuronal integrity (Khanna *et al*, 2011). These data are of particular importance since calorie restriction ameliorates age‐associated memory impairment (Means *et al*, 1993; Dong *et al*, 2016). In turn, elevated levels of miR‐181a‐5p were implicated in cell death after cerebral ischemia, whereas low levels were associated with neuronal survival (Ouyang *et al*, 2012; Moon *et al*, 2013; Xu *et al*, 2015). In addition, miR‐181a‐5p was found in synapses, where it controls surface levels of key mRNAs linked to memory function such as AMPA‐type glutamate receptor (Saba *et al*, 2012) or CamKII (Sambandan *et al*, 2017). While there are no data on the role of miR‐148a‐3p in brain function, miR‐146a‐5p has been linked to neuroinflammatory processes (Saba *et al*, 2014), which is in line with our data showing that overexpression of miR‐146a‐5p in immortalized microglia cells induced inflammatory pathways. It will also be interesting to study the molecular processes that control the expression of the 3 microRNAs in future experiments. In sum, these data further support the view that microRNAs are very suitable molecular biomarker since changes in their expression reflect alterations of multiple cellular pathways. In case of the 3‐microRNA signature, these are mechanisms related to neuronal plasticity and inflammation. Thus, analysis of the 3‐microRNA signature as a molecular marker for cognitive status and reserve could be a complimentary approach to the analysis of blood biomarkers reflecting specific AD pathology or neurodegeneration (Olsson *et al*, 2016; Blennow, 2017; Jack *et al*, 2018; Li & Mielke, 2019; Preische *et al*, 2019). This view is further supported by our finding that the 3‐microRNA signature measured in blood from MCI patients and controls is performing similar to the CSF analysis of the Aβ42/40 ratio—an established AD biomarker—at a time point when phospho‐tau levels are still similar among groups. Similar to these established AD and neurodegeneration marker, there is also evidence that CNS‐derived microRNAs can be released to the circulation and could thus reflect CNS‐related processes (Liang *et al*, 2012; Zhang *et al*, 2017; Lepko *et al*, 2018). Support for the view stems from our findings that targeting the 3‐microRNA signature via anti‐miRs partially ameliorates aberrant hippocampal gene expression and improved memory function in two mouse models for cognitive decline. Nevertheless, future research will need to specifically address the question if the altered expression of the 3‐microRNA signature in blood is directly linked to corresponding expression changes in the brain. Support for this view stems from data showing that microRNAs are released from brain cells via extracellular vesicles (EV) (Xu *et al*, 2017; Men *et al*, 2019) and that such brain‐derived Evs can be detected in blood (Fiandaca *et al*, 2015). In our study, we analyzed RNA from whole blood samples via Pax Gene tubes that should include all RNAs present in blood, including RNA from EVs. However, we cannot exclude the possible that the observed changes in the 3‐microRNA signature in blood originate from other organs or blood cells and that the concomitant regulation in blood and brain is only a coincidence. Another possibility that cannot be excluded at present is that changes in the composition of blood cells contribute to the observed expression changes. In addition, while we analyzed data from mice and humans, it will be interesting to study the role of the 3 microRNAs in cognitive aging in other mammals.

Our findings are of particular importance, since RNA‐based therapeutics are gaining increasing interest (Rupaimoole *et al*, 2017; Hanna *et al*, 2019) and first RNA drugs have been recently approved for clinical use or are in phase III clinical testing (Editorial, 2019). The analysis of circulating microRNAs that inform about CNS mechanisms may therefore also be suitable to develop corresponding stratified RNA‐based therapies. Indeed, several promising clinical studies support the use of RNA‐based drugs for CNS disease (Bennett *et al*, 2019; Tabrizi *et al*, 2019). While especially for studies that are based on ASOs targeting the expression of disease‐causing genes, the therapeutic mechanisms are obvious, the precise mechanisms by which targeting the reported 3‐microRNA signature ameliorates cognitive decline in the employed mouse models need to be further studied in future research. It will in this context also be interesting for future research to link the expression of the 3‐microRNA signature to pathological changes observed via structural and functional brain imaging, which should allow us to more precisely link the observed therapeutic effect to other brain regions in addition to the hippocampus (Lee *et al*, 2019). Since age‐associated memory decline and AD pathogenesis are due to complex combinations of risk factors, it is likely the observed therapeutic effect is also linked to the regulation of several molecular processes, which is in agreement with the function of microRNAs.

The fact that the analysis of circulating microRNAs is minimal invasive that microRNAs are stable in cell‐free environments and are rather resistant to procedures that would normally cause RNA and protein degradation further supports their potential as suitable biomarker. Moreover, the quantitative analysis of microRNAs is comparatively easy and highly reproducible. While in this study, we employed small RNA sequencing and qPCR approaches, recent data suggest that alternative methods such as lateral flow assays can be used for microRNA quantification (Zheng *et al*, 2018). Moreover, there is evidence that microRNA levels assayed via qPCR from venous blood and plasma are comparably to the levels obtained via the analysis of capillary blood obtained from a finger‐prick (Vliegenthart *et al*, 2017). These data support the idea that the analysis of the 3‐microRNA signature could be suitable to develop a point‐of‐care or even home‐based test that would allow minimal invasive and low‐cost screening approaches that inform about molecular processes related to the cognitive reserve and help to detect individuals at risk for cognitive decline. While such a test would not be specific for any cognitive disease, the analysis of blood‐based biomarker should be considered as a first step in a multistep process to identify individuals at risk for pathological memory decline. Risk individuals could then undergo more detailed diagnostic tests that are not suitable for general screening approaches such as the analysis of CSF biomarker or brain imaging. In conclusion, the microRNA‐based screening approach presented in this study could improve the early detection of individuals at risk for pathological cognitive decline and increase the chance for efficient therapeutic intervention using either existing therapeutic strategies or novel RNA‐based approaches targeted toward the 3‐microRNA signature.

## Materials and Methods

### Animals

Male C57B/6J wild‐type mice were purchased from Janvier Labs. All animals were single housed in standard cages on 12‐h/12‐h light/dark cycle with food and water ad libitum. All experiments were performed according to the protocols approved by local ethics committee. 12‐month‐old mice were received and divided into two groups: hom ecage and learning. Mice performing water maze trainings belonged to learning group while the home cage group did not perform any behavior test. APP/PS1‐21 mice used in this study were from Tg(Thy1‐APPSw, Thy1‐PSEN1*L166P)21Jckr colony. Mice of 3 and 16.5 months were sacrificed by cervical dislocation, and whole brain was isolated in ice‐cold Dulbecco's phosphate‐buffered salt (DPBS, PAN‐biotech GmbH) supplemented with EDTA‐free protease inhibitor cocktail (Roche). The ACC, CA1, CA3, and DG regions were micro‐dissected, snap‐frozen in liquid nitrogen, and stored at −80°C.

### Human subjects

All experiments involving human data were approved by the local ethics committee and conformed to the NIH Belmont report. Informed consent was obtained for all subjects. For finding microRNA modules linked to cognition, we analyzed 132 healthy individuals who participated in the PsyCourse study (Budde *et al*, 2018). Briefly, adult healthy individuals were recruited for this study as control participants. The following neuropsychological tests were performed: digit span (forward and backward), digit symbol test, trail making test, and the multiple choice vocabulary intelligence test (MWT‐B); for details, see Budde *et al* (2018). Samples were excluded from the study if they had ever been detected as patient for mental and behavior disorder of ICD‐10 diagnoses. The study was approved by the local ethics committee which was in accordance with the declaration of Helsinki. A composite score considering all the psychological tests was calculated following a similar approach previously described (Hassenstab *et al*, 2015). Briefly, for exploratory factor analysis on cognitive test scores, number of optimum latent factors (*n* = 3) was determined based on model fit score RMSEA 0.02 and parallel analysis (Franklin *et al*, 1995). Next, exploratory factor analysis was performed using fa function of psych package and the results were visualized using fa.diagram function of psych package. Based on results from factor analysis, cognitive tests were grouped into three cognitive domains. Based on the low loadings (< 0.4) on the factors, multiple choice vocabulary intelligence and trail making test error‐A tests were discarded from the analysis. First domain was measured by trail making tests and digit symbol test. Similar factor analysis‐based domain consisting of trail making test and digit symbol test was reported and termed as executive function domain in previous study (Hayden *et al*, 2011). Second domain comprised of digits forward, digits backward and could be described as “working memory” domain. Errors in trail making tests (part B) were part of the third cognitive domain. Having the cognitive domains defined, individual test score was standardized by subtracting the mean from the score and dividing by the standard deviation. Next, the standardized test scores were used to calculate a cumulative domain‐specific cognitive score and finally, domain‐specific cumulative scores were averaged to obtain a global‐weighted cognitive performance score. Higher‐ and lower‐weighted scores represent good and poor performance, respectively. A cohort of patients with mild cognitive impairment and healthy subjects were recruited from the longitudinal cognitive impairment and dementia study (DELCODE) (https://www.dzne.de/en/research/studies/clinical‐studies/delcode/; Jessen *et al*, 2018).

### Water maze

A circular pool (diameter 1.2 m) was filled with white opaque water. An escape platform (11 × 11 cm) was submerged below the water surface in the center of one of the four quadrants (target quadrant). The position of the platform was maintained for all swim trails throughout the test. At 12 months, first water maze training was performed to habituate mice with the training environment. Animals were tested for visual performance by putting a colored cue on the platform. In order to do the spatial memory‐learning test and to check cognitive flexibility, during the later training sessions at 13.5, 15 months, and 16.5 months, platform was put at the center of a new quadrant. However, the position of platform was kept unchanged throughout the corresponding training session. Different visual cues on four sites of the pool were not changed during training trials either. The swimming behavior of the mice was recorded by a camera set on top of the water pool and was analyzed by VideoMot2 (TSE). At each training session, mice were trained to swim to the hidden platform in four daily trials, starting from pseudorandomly varied locations. Each trial consisted of 60 s. If mouse failed to find the platform in due time, it was gently guided to it. Once reaching the platform, the mouse was allowed to have rest for 15 s. After four consecutive trials per day, each mouse was returned to its home cage where it rested until the next day of training. Sixty‐second long probe trial was performed without the platform after each training session at a given month. After last probe test performed at 16.5 months, animals were tested for visual acuity again. Similar experiment was performed for aging and APP/PS1 mice treated with scrambled control and anti‐miR‐mix. For data analysis, features from TSE VideoMot2 software were used. For occupancy plot, 50 × 50 grids defined a bin and number of mouse crossing through each grid was summed up. Densities were calculated from the ratio of total number of crossings through each grid and total number of crossings in the bin. For plotting, a smoothened technique was applied to the densities and minimum and maximum densities out of all experimental groups were taken into consideration during normalization step. For in‐depth feature analysis from water maze data, a modified version of MUST‐C algorithm was used (Illouz *et al*, 2016). In addition to the features described in original paper, we extracted additional features from raw water maze data including total coverage, distance from centroid to platform, and path efficiency. The definition of the features extracted from water maze data is as follows: (i) Average proximity to platform: mean of Euclidean distances from all positions to the platform. (ii) Distance from centroid to platform: Euclidean distance between center of all positions and platform. (iii) Sum of absolute angles: sum of angles between pairs of sequential vectors. (iv) Mean distance from perimeter: mean distance to the closest point on the pool`s perimeter. (v) Total duration: total time mice spent in searching the platform. (vi) Number of quadrant changes: total number of quadrant changes during trial. (vii) Maximal time at one quadrant: percentage of time spent in a single quadrant. (viii) Sum of relative angles: Positive and negative angles are marked from positive and negative movements along X‐axis, respectively. (ix) Cumulative angle along Y‐axis: Similar to angles along X‐axis, relative angles along Y‐axis are calculated. (x) Platform crossings: number of crossings of the platform region (probe test). K)Variance of distance from perimeter: variance of Euclidean distances from the pool´s perimeter. (xi) Mean velocity: ratio between total distance and total time spent. (xii) Distance traveled: sum of Euclidean distance between each pair of sequential locations. (xiii) Total coverage: ratio between area of convex hull of all points and the area of the pool. (xiv) Local densities: mean distance between all pairs (x,y coordinates) of locations in the trial. (xv) Variance of distance to platform: variance of Euclidean distance from all positions to the platform. (xvi) Path efficiency: ratio of the path length to the Euclidean distance between starting and endpoints. Path length is sum of Euclidean distances between all consecutive points in the trial. Based on all these features, scatter plots were generated, and one of the following search strategies was assigned to each trial of the training session. Weighted cognitive score per day was calculated according to the formula as follows.

$$\sqrt{\text{mean}\;\text{cumulative}\;\text{strategy}\;\text{score}=\frac{1}{n}*\sum_{i=1}^{n}\left[\frac{\text{Sdfi}*\text{Sdc}}{\text{mc}}+\frac{\text{Sscfi}*\text{Sscc}}{\text{mc}}+\frac{\text{Slcfi}*\text{Slcc}}{\text{mc}}\right]}$$

n = total trial number per day; Sdf_i_ = frequency of direct strategy in i^th^ trial; Sdc = given strategy score for direct search; 10; mc = total number of mice; Sscf_i_ = frequency of corrected strategy in *i* ^th^ trial; Sscc = given strategy score for corrected search; 9.5; Slcf_i_ = frequency of short chaining strategy in *i* ^th^ trial; Slcc = given strategy score for short chaining search; 9.

### Collection of blood from mice and purification of total RNA from blood and brain sub‐regions

Blood was collected from retro‐orbital sinus of all mice for first time at 12 months of age before starting any behavioral experiment. Next blood collections were done after 24 h of finishing probe tests at 13.5, 15, and 16.5 months. In summary, individual mouse was anesthetized with isoflurane (1.8%) for 2–3 min. Afterward, 150–200 µl blood was collected from the orbital sinus of animals using a heparinized glass microcapillary and stored in tubes provided in RNeasy Protect Animal Blood System (Qiagen, Hilden, Germany). Each time blood was collected from alternative eyes. The blood tubes were allowed to stand at room temperature for 24 h and then stored at −20°C. Purification of total RNA from blood was performed following the manual of RNeasy Animal Blood Protect Kit (Qiagen, Hilden, Germany). Briefly, blood tubes were centrifuged for 3 min at 5000xg at room temperature. After removing the supernatant without disturbing pellet, 1 ml of RNAse‐free water was added. The solution was vortexed and centrifuged in the same condition as mentioned above. Supernatant was discarded, and 240 µl Buffer RSB was added to pellets. After resuspending the pellet properly, appropriate amount of buffer RBT and proteinase K was added and the solution was incubated for 10 min at 55°C. Next, the solution was applied on the membrane of the QIAshredder spin column and centrifuged for 3 min at the maximum speed. After centrifugation, 1.5 volume of 100% ethanol was added to the flow‐through. Later, the solution was applied into a 2 ml RNeasy MinElute Spin column and centrifuged for 1 min at 10,000× *g*. The membrane of spin column was washed once with 350 µl of Buffer RWT, and DNase treatment was performed for 15 min at room temperature. Next, 350 µl of buffer RWT was added to the column followed by centrifugation for 15 s at 10,000× *g*. Column was washed with 500 µl Buffer RPE (centrifugation for 15 s, at 10,000× *g*, at room temperature). The final wash was performed with freshly prepared 80% ethanol followed by centrifugation for 2 min at 10,000× *g*. RNA was eluted with REB buffer after a short centrifugation (10,000× *g*, 1 min). Extracted RNA was incubated at 65°C for 5 min and immediately placed on ice. Concentration of RNA was measured on NanoDrop, and isolated RNA was stored at −80°C for future use. Tissue sections from mouse ACC, dentate gyrus, CA1, and CA3 were homogenized in Tri reagent (Tri Reagent, Sigma‐Aldrich, Germany) followed by RNA extraction according to manufacturer’s instruction of RNA clean and concentrator kit (Zymo Research).

### Blood collection in humans

Blood was collected from the median cubital vein. Before collection, the collection tubes were stored at 18–25°C. For the collection of blood via PAXgene tubes, 2.7 ml of blood was filled into the tube that was subsequently gently inverted 10 times. Tubes were stored for 2 h at room temperature and then transferred to −20°C for 24–72 h before being stored at −80°C until further processing. For the isolation of PBMCs, blood was collected in CPT tubes that were subsequently inverted eight times and then centrifuged for 30 min (1,700× *g*) at room temperature. Using a pipette, the plasma was removed (until 1cm above the white lymphocyte layer). The white lymphocyte layer was then transferred into a 15‐ml Falcon tube using a pipette. Subsequently, the tube was filled with up to 15 ml with PBS and gently inverted five times. The Falcon tube was centrifuged for 15 min (300× *g*, with brake) at room temperature, the supernatant was discarded, and the pellet was resuspended in 15 ml PBS. These steps were repeated in total 3 times. The cell pellet was eventually resuspended in 2 ml of PBS and frozen in liquid nitrogen before being stored at −80°C. For plasma samples, blood was collected into EDTA‐plasma tubes and centrifuged from 10 min (2,000× *g*, without brake) at room temperature. The supernatant was aliquoted into cryo‐tubes into and stored within 30 min at −80°C. Exosomes were isolated from plasma via a number of subsequent centrifugation steps at 4°C: 3,500× *g* for 10 min, two times 4,500× *g* for 10 min, 10,000× *g* for 30 min, and 100,000× *g* for 60 min. The 100,000× *g* pellet was washed once with phosphate‐buffered saline (PBS) at 100,000*g* for 60 min before resuspension in PBS.

### High‐throughput Small RNA sequencing and bioinformatic analysis

Next‐generation sequencing of the RNA from collected blood samples and brain regions from mouse were performed using TruSeq® Small RNA kit according to manufacturer´s protocol (Illumina, San Diego, CA, USA). For human samples, sequencing libraries were prepared using NEBNext® small RNA library preparation kit according to manufacturer´s instruction. Briefly, 100 ng RNA was used as starting material followed by adapter ligation and primer hybridization. First strand of cDNA was generated followed by PCR enrichment. Libraries were pooled, and PAGE was run for size selection. For small RNAome, ˜150 bp band was cut and used for library quantification after purification. A final library concentration of 2 nM was used for sequencing. Sequencing was performed using a 50‐bp single read setup on the Illumina HiSeq 2000 platform. Demultiplexing was done using Illumina CASAVA 1.8. Sequencing adapters were removed using cutadapt‐1.8.1. Sequencing quality including total number of reads, percentage of GC content, sequence quality per base, N content per base, sequence length distribution, duplication levels, overrepresented sequences, and Kmer content was investigated using FastQC v0.11.5 (http://www.bioinformatics.babraham. ac.uk/projects/fastqc/). For quantification of mature microRNAs, miRDeep2 was primarily used. Mouse (mm10) and human (hg38) genome sequences were retrieved from UCSC (https://genome.ucsc.edu/) Genome Browser, and corresponding genomic index files were created using Bowtie‐build tool (version 1.12) with default options. Sequencing reads were mapped to the reference genome using mapper.pl script with default settings in the miRDeep2 package. An example is outlined below: mapper.pl config.txt ‐d ‐e ‐h ‐i ‐j ‐k TGGAATTCTCGGGTGCCAAGG ‐l 18 ‐m ‐p genome_index_file ‐s fasta_file ‐t mapped_coordinates.arf ‐v.

Reads less than 18 nucleotides were discarded, and miRDeep2 module with default options was used to identify known and novel microRNAs in sequencing data. To quantify known microRNAs, sequencing reads, the known mature microRNAs, and optionally its star sequences for mouse were mapped against the known precursor microRNAs for reference genome documented in miRBase (Release 21), allowing 0 mismatch. miRDeep2.pl function from miRDeep2 with default settings was used for this purpose. For example: miRDeep2.pl fasta_file genome_index mapped_coordinates.arf mature_mirna_same_species.fa mature_mirna_other_species.fa precursor_mirna_same_species.fa ‐t species_name.

A read was assumed to represent a sequenced mature microRNA, if it fell within the same position on the precursor as mature microRNA, plus 2 nt upstream and 5 nt downstream. For the prediction of novel microRNAs, mature microRNAs in Rattus norvegicus (miRBase release 21) were provided as related species for mouse. On the other hand, mature microRNAs in Gorilla gorilla, Pongo pygmaeus, and Pan troglodytes (miRBase release 21) were provided as related species for human. However, novel microRNAs are not discussed in the current study. The expression counts for mature microRNAs originating from multiple genomic locations were summed up. A more detail of the miRDeep2 method is available at https://www.mdc‐berlin.de/content/mirdeep2‐documentation. For differential expression analysis, prior to differential expression analysis, microRNAs with low reads were filtered out for downstream analysis. Unwanted variation factors were estimated based on reads in replicate samples, and these variants were removed from sequencing data using RUVs function of RUVSeq package. Differential expression analysis was performed using DESeq2 (Love *et al*, 2014). MicroRNAs having adjusted *P*‐value < 0.05 were considered as significantly deregulated.

### Statistical framework for analyzing microRNA signature

Sequencing data were normalized for library size and log2 transformed. A quality *Z*‐score was calculated for each sample, and samples with low quality (*Z* > 2.5 or *Z* <−2.5) were defined as outlier and removed from further analysis. Surrogate variables were determined by sva R‐package. Unless otherwise stated, the effects of surrogate variables and biological covariates (e.g., age, sex) were normalized together using a linear regression model. These normalized data were used for subsequent analysis. For the given set of microRNAs, corresponding expression data were considered to calculate an eigenvalue. Eigenvalue was calculated using svd function in R as previously described (Alter *et al*, 2000) and used for comparative analysis. Samples with low‐quality *Z*‐score (*Z* > 2.5 or *Z* < −2.5) of eigenvalue were filtered out for downstream comparative analysis between conditions. For statistical analysis, Wilcoxon test (using wilcox.test in R or stat_compare_means in ggpubr package) was used. Boxplots were made using the ggboxplot function of ggpubr package in R. A meta‐analysis was performed to get insight about the pooled effect and consistency of the findings across multiple studies. For this purpose, Cohen´d effect size along with lower and upper intervals was calculated using cohen.d() function in R. The standard error and variance within study was calculated using stdErr and var functions in R. A random effect model based meta‐analysis was performed across studies using metagen function of meta package in R. Standardized mean difference (SMD) for the pooled effects was estimated, and Sidik–Jonkman estimator has been used to estimate the between‐study variances. The observed *P*‐values from single studies were further adjusted across studies with Benjamini–Hochberg (BH) method and using *P*.adjust function in R. For randomization, a seed of 1,234 was used and meta‐analyses of 1,000 combinations of three random microRNAs from a pool of 55 aging responsive microRNAs were performed. The observed *P*‐value of the pooled effect was adjusted with the multiple corrections (BH method) considering the total number of random combinations [e.g., *P*.adjust(*P*‐value, *n* = 1,000, method = "BH")]. Randomized combinations (1,000 times) of three microRNAs from human microRNAome were also tested with the similar approach as described above. For PsyCourse cohort, age effect was not regressed to assess the effect of aging in microRNA expression. However, other technical and biological covariates were adjusted as described above. To determine optimum number of clusters for k‐means clustering in MCI patients, elbow method was implemented. Dissimilarity matrix based on Euclidean distance was constructed after scaling of normalized data. Complete linkage of hierarchical clustering was determined, and the dendrogram was cut by the optimum number of clusters. Two R‐packages cluster and factoextra were used for these purposes.

### Rank‐rank hypergeometric overlap analysis

The significance overlap of expressed microRNAs between two cohorts was calculated using the rank‐rank hypergeometric test (Plaisier *et al*, 2010). Briefly, microRNAs were ranked by expression, placing the most expressed microRNA at the top and the least expressed at the bottom of the list. Number of overlapping microRNAs between two cohorts was counted at every 10^th^ combination, and Fisher’s exact test was used to calculate significance of the overlap and later was corrected after multiple statistical adjustments.

### High‐throughput mRNA sequencing and bioinformatic analysis

Library preparation for mRNA sequencing was performed according to Illumina TruSeq and as previously described (Jain *et al*, 2019). RIN values of all samples were above 8. Briefly, libraries were prepared from 500 ng of input RNA. The quality of RNA was checked on Agilent 2100 Bioanalyzer (Agilent Technologies), and prepared libraries were quantified using a Qubit 2.0 Fluorometer (Life Technologies). Similar to small RNAome, a final library concentration of 2 nM was used for 50 bp single‐end sequencing on Illumina HiSeq 2000 platform. For sequencing, samples from different experimental groups were pooled to control for sequencing biases. Base calling and generation of fastq files were accomplished by bcl2fastq (v.2.18.0). Quality of the raw sequencing data was checked using FASTQC (v0.11.5) (http://www.bioinformatics.babraham.ac.uk/projects/fastqc/) and was above 30 for all samples. Sequencing reads were mapped to corresponding reference transcriptomes (mm10 and hg38) using STAR aligner (v2.5.2b). After mapping, raw count files were generated using featureCounts of subread package (v1.5.1). Raw mapped read counts for genes (≥ 5 in at least 50% of studied samples) were filtered for downstream analysis. Differential expression analysis was performed using DESeq2(Love *et al*, 2014). Genes having *P*‐value < 0.05 after multiple adjustments were considered as significantly deregulated.

### Weighted co‐expression analysis

MicroRNAs that are highly correlated with composite cognitive score in PsyCourse cohort were identified and summarized with a modular eigengene profile using the weighted gene co‐expression network analysis (WGCNA) package (version 1.61) in R. Individuals used for this analysis were cognitively healthy and young in age (25.95 ± 5.1 years, mean ± SD), and no further experimental groups were defined. The number of males and females was not matched (74 males and 58 females). Therefore, the normalized counts were adjusted for sex effect and adjusted counts were log (base 2) transformed. A downstream quality check step using a *Z*‐score calculated for each sample confirmed high quality of the analyzed data. Next, the transformed data were used to calculate pair‐wise bi‐weighted mid‐correlations between microRNAs. Next, a soft threshold power of 9 was chosen based on approximate scale‐free topology to highlight strong correlations (*R* ^2^ = 0.90) and used to calculate pair‐wise topological overlap between microRNAs in order to construct a signed microRNA network. Modules of co‐expressed microRNAs with a minimum module size of 20 were later identified using cutreeDynamic function with following parameters: method = “hybrid”, deepSplit = 3, pamRespectsDendro = T, pamStage = T. Stability of the detected modules, particularly for blue and brown modules, was tested using different module (5,10, 20) and deep split (0‐4) sizes. Closely related modules were merged using dissimilarity correlation threshold of 0.15. Different modules were summarized as network of modular eigengenes, (MEs) which were then correlated with the composite cognitive score calculated as mentioned above. The module membership (MM) of microRNAs was defined as the correlation of microRNA expression profile with MEs, and a correlation coefficient cutoff of 0.60 was set to select the module‐specific microRNAs. Pearson correlation of MEs and cognitive score was plotted as heat map. To identify unbiased gene expression modules in aging and APP/PS1‐21 treated with inhibitor cocktail, similar co‐expression analyses were performed. As described above, a signed similarity matrix was created using bi‐weighted mid‐correlations for all pairs of genes. The signed adjacency matrix was set at power 17 and 10, respectively, for aging and APP/PS1‐21 data to reduce the emphasis of weak correlations and to highlight the strong correlations (*R* ^2^ = 0.80). Modules were defined with minimum module size 100 and deep split = 2, and modules with correlation greater than 0.85 were merged together.

### MicroRNA feature selection

The feature selection analysis was based on a regression approach given that the PCA‐based cognitive score is a real value. A composite score for representing cognitive ability was obtained by compressing all the water maze features into a single quantity by means of projecting those features over their first principal component (i.e., PCA’s first component). Given our interest on determining the best (sub)‐set of microRNAs that link to the aforementioned cognitive score, we applied three different recursive feature elimination approaches that were based on either the random forest (Breiman, 2001) or the support vector machine(Cortes & Vapnik, 1995) regression algorithms, RF and SVM in the subsequent. In all these approaches, the cognitive score was used as outcome variable whereas the counts from the 55 aging‐related microRNAs were used as predictors. Two independent RF regression models were trained, one using bootstrapping and the other leave‐one‐out cross‐validation as sampling procedures. The Caret R‐package was used to implement all the feature selection algorithms. Caret’s RFE control function was applied with the following parameters: RF‐bootstrapping (functions = rfFuncs, method = boot, number = 250, repeats = 1, *P* = 0.75); RF‐leave‐one‐out (functions = rfFuncs, method = LOOCV, number = 1, repeats = 1); SVM (number = 100) with an RBF kernel; and size parameter set to c (2, 5, 10, 20). Software versions were v.3.3.1 and v.6.0.76 for R and Caret, respectively.

### Gene ontology and pathway analysis of microRNA target genes

Lists of predicted microRNA target genes were downloaded from TargetScan (v 7.1). All experimentally validated microRNA target genes in mouse were retrieved from miRTarBase (v 7.0) (http://mirtarbase.mbc.nctu.edu.tw/). KEGG pathways were annotated using the enrichKEGG function in clusterProfiler. *P*‐value cutoff was set at 0.05, and multiple adjustment method “BH'' was chosen. KEGG pathways for each module were merged using the merge_result function in clusterProfiler. Top 5 significant pathways from each module were selected, and comparative analyses were displayed using dotplot function in clusterProfiler. The dots in the plot were colored by adjusted *P*‐value.

Gene ontology analysis for biological processes was performed using the enrichGO function of clusterProfiler package in R at default setting. Unless otherwise stated, the redundant GO terms were systematically reduced by grouping similar terms based on their semantic similarity. To this end, a score of similarity matrix between terms was calculated using calculateSimMatrix function in the rrvgo package at default setting. A similarity threshold of 0.8 was used for grouping the terms based on similarity and was applied using reduceSimMatrix function in the rrvgo package. The grouped/cumulative *P*‐value for the group term was calculated by combining *P*‐values of the member terms using Fisher's method. The cumulative *P*‐value was further adjusted with Bonferroni method, and grouped terms with adjusted *P*‐value < 0.05 were considered as significant.

### Preparation of microRNA lipid nanoparticles

Lyophilized microRNA mimic or inhibitors were encapsulated into lipid nanoparticles according to manufacturer´s protocol (Neuro9 siRNA Spark, Precision NanoSystems). Briefly, 5 nmol lyophilized microRNA mimic or inhibitor was dissolved in nucleic acid storage buffer to make final concentration of 1 mM. Required amount of nucleic acids and formulation buffer 1 (FB1) [miRNA + FB1 mix] was mixed to have the final concentration at 930 µg/ml. Spark formulation buffer 2 (FB2), miRNA + FB1 mix, and lipid nanoparticles were added to the cartridge, and mimics/inhibitors of miRNAs were encapsulated into lipid nanoparticles using the NanoAssembler Spark. List of microRNA mimic, inhibitors, and control oligonucleotides used to package into lipid nanoparticles is summarized in Dataset EV17.

### Cellular models of human and mouse brains

#### Primary neuronal culture

E17 pregnant mouse of CD1 background (Janvier Labs, France) was sacrificed by cervical dislocation. 8–14 embryos were decapitated to dissect 16–28 hippocampi those were pooled in a 15‐ml falcon tube containing ice‐cold PBS (Pan Biotech). Next, the tissue was dissociated in a solution of pre‐warmed PBS plus 2.5% trypsin‐EDTA (Gibco, USA) followed by incubation at 37°C for 13 min. Trypsinization was stopped by adding processing medium (Neurobasal® Medium 1X (Gibco, USA) supplemented with 10% FBS and 1% P/S and washed. Next, dissociated tissue was homogenized in processing media and centrifuged at 300× *g* for 5 min and cell pellets were resuspended in maintenance medium (Neurobasal Medium, 2% B27, 1% P/S, 1% GlutaMAX). Cells were counted in a Neubauer counting chamber and seeded at density of 130,000 cells/well in 24‐well plates that were previously coated with 0.05 mg/l poly‐D‐Lyine (PDL, Sigma‐Aldrich, Germany). The day of plating the cells was considered as day 0 (DIV0), and one third of the media was changed with new maintenance media in every 2–3 days. For RNA‐seq experiments, neurons were treated at DIV10, with either miRNA mimic LNPs or the scrambled RNA LNPs at a dose of 0.01 µg/ml and ApoE4 (1 µg/ml). After 48 h of incubation, neurons were harvested in TRIzol for RNA isolation. RNA was prepared using RNA clean and Concentrator kit (Zymogen) according to manufacturer’s instruction.

#### Immortalized microglial cells

Immortalized microglial (IMG) cells were purchased from Merck (Cat. No. SCC134) and maintained in DMEM high glucose (Sigma) supplemented with 10% fetal bovine serum (FBS) (Merck Millipore), 1× L‐Glutamine (Merck Millipore), and 100 U/ml penicillin–streptomycin. Cells were seeded in a 24‐well culture plate with 25,000 cells per well as seeding density. Culture plates were kept at 37°c with 5% CO_2_, 95% air in incubators. The medium was changed the next day 1 h prior to transfection. For transfection in these differentiating cells, ApoE4 (1 µg/ml) was added to the cells followed by treatment with miR‐146a‐5p mimic nanoparticle mixture at a dose of 0.05 µg/ml. Cells treated with equal amount of scrambled RNA‐nanoparticle mix were used as controls. The cells were harvested after 48 h for RNA isolation. RNA was prepared as described above.

#### Bioengineered neuronal organoids

Bioengineered neuronal organoids (BENOs) were generated from induced pluripotent stem cell line named TC1133 (originally known asLiPSC‐GR1.1) iPSC cell (Lonza, USA). Organoid generation and maintenance steps were performed as previously described (Zafeiriou *et al*, 2020). BENOs at day *in vitro* (DIV) 64 were used for lipid nanoparticle‐based transfection. BENO was treated with 3‐miR mimic LNP mix or negative control LNP at a dose of 0.03 µg/ml. After 24 h of transfection, BENOs were harvested, snap‐frozen at liquid nitrogen, and later stored at −80°C until further use. RNA was prepared as described above.

### Cell type‐specific expression analysis

Neuron‐enriched primary neuronal culture was prepared as described above except a different maintenance media supplemented with Neurobasal plus, 2% B27 plus along with 1% penicillin–streptomycin, and 1% GlutaMAX was used. Primary astrocytes from mouse P1 pups were prepared following a previously described protocol(https://www.jove.com/de/v/56092/culturing‐vivo‐like‐murine‐astrocytes‐using‐fast‐simple‐inexpensive). Primary mouse microglia cell cultures were prepared from P1 mouse pups as previously described (https://bio‐protocol.org/pdf/bio‐protocol1989.pdf). In summary, mixed glial cells were grown in DMEM (Thermo Fisher Scientific) with 10% FBS, 20% L929 conditioned medium, and 100 U/ml penicillin–streptomycin (Thermo Fisher Scientific). Microglia were collected through shaking after 10–12 days *in vitro*, counted, and plated in DMEM supplemented with 10% FBS, 20% L929 conditioned medium, and 100 U/ml penicillin–streptomycin. Microglia were shaken off up to two times. Corresponding cell types (e.g., neurons, astrocytes, microglia) were treated with TRIzol, and RNA was prepared as described above for qPCR‐based quantification of miR‐146a‐5p, miR‐148a‐3p, and miR‐181a‐5p.

### Hippocampal injections

Stereotaxic injections to hippocampal CA region were performed as described previously with modifications (Zovoilis *et al*, 2011). In summary, mice were anaesthetized and glass micropipette containing lipid nanoparticles was directly inserted into the dorsal hippocampus using the following coordinates: 1.0 mm posterior to the bregma; ± 1.0 mm lateral and 1.5 mm ventral from midline. MicroRNA mimics, control for mimic (dose: 0.1 µg/ml) and inhibitor mix, and control for inhibitor (dose: 0.2 µg/ml) were injected bilaterally (1 μl, at a rate of 0.3 μl/min per side).

### STED and confocal imaging and data analysis

Neurons were treated with 3‐miR mimic mix or the control LNPs (0.03 µg/ml) at DIV7. At DIV10, neurons were fixed and quenched with 4% PFA (Sigma‐Aldrich, Germany) and 100mM NH_4_Cl (Merck, Germany), respectively, at room temperature for 30 min each. Next, cells were washed three times on a shaker using permeabilization and blocking buffer (0.1% Triton‐X [Merck, Germany] + 3% bovine serum albumin (BSA) [AppliChem GmbH, Germany]). Primary antibody was then added, and cells were incubated for 1 h at room temperature. Next, cells were washed and incubated with secondary antibody for 1 h at room temperature. After washing with PBS, coverslips were mounted on glass slides using mowiol (Merck, Germany). Primary antibodies include the following: synaptophysin 1 (guinea pig, SySy) and PSD‐95 (rabbit, Cell Signaling). As secondary antibodies, Cy3 (donkey, anti‐ guinea pig, Jackson Imm.) and Abberrior STAR 635p (goat, anti‐rabbit) were used. Confocal and STED images were acquired using a multicolor confocal STED microscope (Abberior Instruments GmbH, Göttingen, Germany). Analysis of colocalization of pre‐ and postsynaptic markers was performed using Colocalization and SynQuant plug‐ins in Fiji (v 2.0.0).

For dendrite staining, crystals of DIL (Thermo Fisher, Germany) were used. Briefly, neuronal cells grown on coverslips were treated, fixed, and quenched as described above. For disperse labeling of dendrites, 2–3 crystals of DIL were added to the cells/well of a 24‐well plate and incubated on a shaker for 10 min. Cells were washed three times with 1× PBS and subsequently incubated overnight at 4°C. Next day, coverslips were washed and mounted using mowiol. STED images were acquired using multicolor confocal STED microscope (Abberior Instruments GmbH, Göttingen, Germany). Spine density and total spine length were measured using in house scripts based in Fiji and R.

### Multielectrode assay (MEA)

Mouse hippocampi were extracted, trypsinized, and homogenized as described above. Cells were collected by centrifugation (300 × g for 5 min at RT) and resuspended in NbActiv4 (BrainBits) culture medium. Cells were counted, mixed with laminin (1 µg/ml, Merck), and plated at density of 15,000 neurons/µl into Lumos lens lid 48‐well microelectode array plates (MEA) equipped with 16 electrodes/well. These wells were previously coated with poly‐D‐lysine (PDL). Day of seeding cells was considered as DIV0. One third of the media was changed with new media every 2–3 days. At DIV7, cells were treated with 3‐microRNA mix or scrambled RNA lipid nanoparticles. At DIV10, spontaneous neuronal activity was measured using the Maestro Apex Platform (Axion Biosystems) for 24 h. Recording was done in every 3 h for 10 min during this time.

### Quantitative RT–PCR for microRNA and target genes

cDNAs for quantitative PCR from mRNA were prepared using Transcriptor High Fidelity cDNA Synthesis Kit (Roche). SYBR green (LightCycler® 480 SYBR Green I Master, Roche) was used for quantification, and signals from genes were normalized to those from internal control hypoxanthine phosphoribosyltransferase 1 (HPRT1) gene. Relative gene expression was analyzed by 2−ddCt method (Livak *et al*, 2001). List of primer sequences is summarized in Dataset EV17.

### Luciferase assay

Cloned 3′UTR sequence of LRRK2, SLC6A11, and CADM3 (mm10) and scrambles UTR were purchased from GeneCopoeia. UTR was cloned downstream to firefly luciferase of pEZX‐MT06 Dual‐Luciferase miTarget™ vector. The pEZX‐MT06‐scrambled UTR or pEZX‐MT06‐LRRK2, SLC6A11, and CADM3 3′UTR construct and miR‐181a‐5p, mir‐148a‐3p, and miR‐146a‐5p mimic or negative control were co‐transfected into HEK293‐T cells cultured in 96‐well plates using EndoFectin™ Max Transfection Reagents (Gene Copoeia) according to the manufacturer’s protocol. 48 hours after transfection, Firefly and Renilla luciferase activities were measured using a Luc‐Pair™ Duo‐Luciferase HS Assay Kit (for high sensitivity) (GeneCopoeia). Ratio of luminescence from the Firefly luciferase to the Renilla luciferase was calculated for each sample. The mean of luciferase activity of Firefly/Renilla was considered for the analysis. The relative luciferase activity was calculated by internal normalization for each sample. Relative luciferase activity was used to determine the micoRNA‐UTR binding.

### Published datasets used in this study

Genes linked to general cognition were retrieved from a recent GWASA study (Davies & Harris, 2018). Cell type‐specific expression of microRNAs was downloaded from NCBI GEO and part of a previous study (Jovičić *et al*, 2013). Immune‐related genes based on eQTL data analysis were reported in the previous study(Gjoneska *et al*, 2015). Raw data of Alzheimer´s model mice Ck‐p25 for 2 and 6 weeks were retrieved from GSE65159 and analyzed. Transcriptomic microarray data from human AD patients and controls were available (GSE44770) and analyzed with GEO2R. Human AD proteome data were downloaded from the previous study(Ping *et al*, 2018). Plasma qPCR array from MCI patients and healthy subjects was available at GEO (GSE90828). Small RNAome data from CSF samples from MCI patients and healthy subjects were retrieved from the previous study (Jain *et al*, 2019) and analyzed in house. Small RNAome data from 6‐month‐old FTLD (fronto‐temporal dementia) mouse models were retrieved from GSE89983. As outlined above in some cases, we employed previously published data that were generated on the basis of microarray studies to compare lists of differentially expressed transcripts. All our experiments that address differential gene or microRNA expression are based on RNA sequencing which is not biased by probe design and has a wider dynamic range when compared to microarray studies. Although a significant overlap among differentially expressed transcripts detected via a microarray and RNA‐seq has been reported (Rao *et al*, 2019), care has to be taken when interpreting such data.

## Data and code availability

Published datasets used in this study are from multiple sources as described above. All high‐throughput sequencing datasets generated in this study from cell culture and mouse experiments are deposited and available in NCBI GEO (Data accession: GSE153105, GSE153106, GSE153107, GSE153109, GSE153110, GSE153111, GSE153112, GSE153180). Due to data protection and privacy policy, we submitted the human data generated in this study to the European Genome‐phenome Archive (Accession number EGAD00001008153), through which researchers can apply for access of the raw data. All analyses are performed using published packages as cited and are available through Bioconductor or CRAN. Custom source code along with processed data is available via the following link (https://github.com/mdrezaulislam/paper_three_mir_signature).

## Author contributions

MRI designed and performed cell culture and all mice‐related experiments, generated and analyzed sequencing and imaging data, interpreted results, made figures, drafted, and revised the manuscript; LK generated small RNAome data from human samples, performed IMG cell culture‐related experiments, validated microRNA expression changes by qPCR, performed luciferase assay, and revised the manuscript. TB contributed to longitudinal analysis of aging mice; TPC performed feature selection based on sequencing and water maze data; VE developed tool for in‐depth water maze data analysis. M‐PZ developed and provided the BENOs. EB contributed to establishment of the lipid nanoparticle‐based transfection protocols, taught stereotactic surgeries, and reviewed the manuscript, MG performed qPCR and imaging experiments related to Fig EV3; SB and CK helped with RNA sequencing experiments; OW helped with water maze experiments, ASO performed qPCR experiments related to Fig 7; MSS contributed to IMG cell culture and qPCR experiments; CB, FB, KB, NCC, KF, MTH, DJ, IK, LK, CL, CDM, MHM, RP, OP, JP, BSR, NR, ASc, ASp, EJS, ST, MT, MW, JW, ED, FJ, and Delcode Study Group generated and analyzed data from the Delcode study; W‐HZ developed and provided the BENOs; SMS and SOR contributed to imaging experiments. UH, MB, FSe, JLK, HB, TGS, and PF collected and analyzed phenotypic data within the PsyCourse cohort; FSa designed the experiment and interpreted results; AF designed the experiment, supervised, interpreted results, made figures, and wrote the manuscript.

## Conflict of interest

The authors M.R.I, F. Sa, and A.F are co‐inventors on a pending patent application (EP20217509.7) entitled “Methods and kits for detecting a risk for developing neurological or neurophysiological disorders”. The other authors declare no conflict of interest.

## Supporting information



Appendix

Expanded View Figures PDF

Dataset EV1

Dataset EV2

Dataset EV3

Dataset EV4d

Dataset EV5

Dataset EV6

Dataset EV7

Dataset EV8

Dataset EV9

Dataset EV10

Dataset EV11

Dataset EV12

Dataset EV13

Dataset EV14

Dataset EV15

Dataset EV16

Dataset EV17
