# Supplementary material for: A microRNA signature that correlates with cognition and is a target against cognitive decline
Source: EMBO Mol Med. 2021 Oct 11;13(11):e13659. doi: 10.15252/emmm.202013659 (PMC8573587; doi:10.15252/emmm.202013659)
Supplement: Supplementary file 1 — Appendix [file EMMM-13-e13659-s010.pdf]

Appendix

Table of Content

**Appendix Item**	**Page**
Appendix Fig. S1 & legend	2-3
Appendix Fig. S2 & legend	4
Appendix Fig. S3 & legend	5
Appendix Fig. S4 & legend	6-7
Appendix Fig. S5 & legend	8
Appendix Fig. S6 & legend	9
Appendix Fig. S7 & legend	10
Appendix Fig. S8 & legend	11
Appendix Fig. S9 & legend	12-13
Appendix Fig. S10 & legend	14
Appendix Fig. S11 & legend	15
Appendix Fig. S12 & legend	16-17
Appendix Fig. S13 & legend	18
References for Appendix SFig. legends	19

Appendix Fig. S1

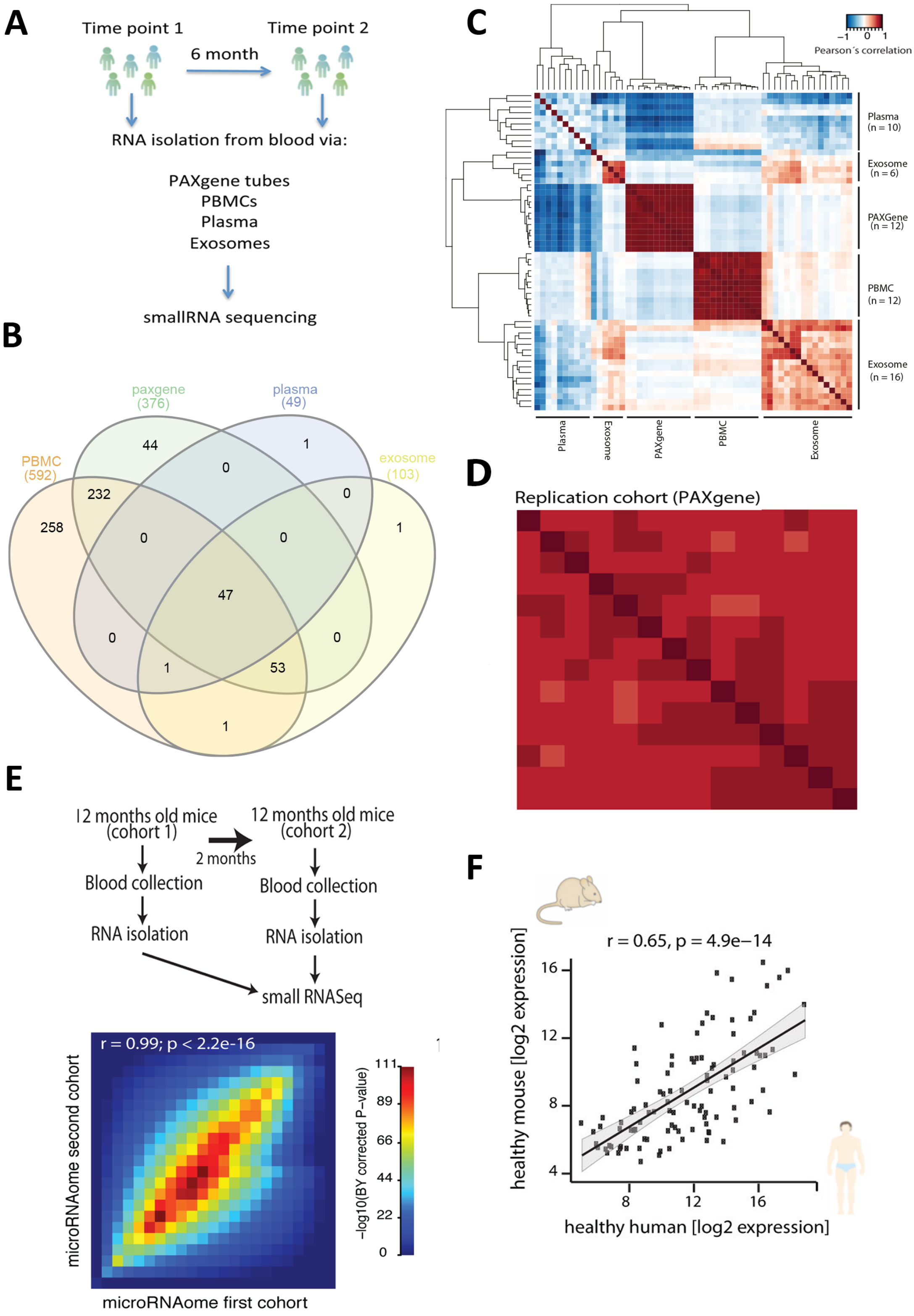

Appendix Fig. S1: Analyzing circulating microRNAs in mice and humans. A. Experimental design of a pilot experiment. We analyzed the circulating microRNA expression profiles in blood samples obtained from healthy individuals at two different time points. We reasoned that the analysis of at least two

timepoints would also enable us to evaluate which method would be most suitable for longitudinal studies, hence the methods that would show lowest variability in healthy individuals at the two time points. RNA was isolated from blood of each individual using four different approaches, namely via PAXgene tubes (Qiagen), from peripheral blood monocytes (PBMC), plasma and blood exosomes. All samples were subjected to smallRNA sequencing (N = 6-22, age = 47 ± 5 years; mean ± s.d). **B.** The Venn diagram shows microRNAs detected via the 4 different approaches at time point 1. In each approach, microRNAs having at least 5 reads in 50% of the samples were considered as expressed microRNAs. In total 47 microRNAs were commonly detected in blood via all four methods at comparable levels. **C.** Heat map showing the correlation of smallRNA-seq data obtained from blood at time point 1 and 2. When comparing the expression of blood microRNAs at time point 1 vs. time point 2, most consistent results were obtained when smallRNA sequencing was performed using RNA isolated via PAXgene tubes or from PBMCs. These data suggest that the analysis of blood microRNAs collected either from PBMCs or via PAXgene tubes yields reliable data that is comparable in healthy individuals, even when analyzed at different time points. **D.** Considering that the isolation of RNA from blood via PAXgene tubes is comparatively easy and can also be performed in a clinical setting without access to a wet lab, we decided to focus on this approach and replicate our observation in an independent experiment. To this end PAXgene blood samples were collected from an additional group of individuals at two different time points. The microRNA expression amongst individuals and amongst time point 1 and 2 was highly correlated as shown by the corresponding heat map. **E.** According to manufacturer, the RNAeasy system (Qiagen) for the isolation of RNA from blood of small animals is based on the same chemistry as the PAXgene (Qiagen) system for human blood collection suggesting that data obtained from humans via PAXgene tubes and mice via the RNAeasy kit would allow optimal comparison. To test this more specifically, we established a protocol for the longitudinal collection of blood from mice followed by smallRNA sequencing (see methods for details). Next, we performed a pilot experiment in mice to test our protocol of blood collection followed by smallRNA sequencing. The upper panel depicts the schematic outline of the pilot experiment (n = 10/cohort). We collected blood from a cohort of 12 months old mice and stored the samples at -80°c (cohort 1). Two months later, the exact procedure was repeated in another cohort of 12 months old mice (cohort 2). We used this approach for initial testing rather than a longitudinal analysis to exclude potential age-related changes considering the comparatively short life span of mice. RNA samples from cohort 1 and cohort 2 were then subjected to small RNA sequencing. The lower panel shows a rank-rank hypergeometric overlap (RRHO) analysis which revealed that the expression of microRNAs detected in cohort 1 and cohort 2 was highly overlapped (rho = 0.9). Each pixel represents overlap between two cohorts, and color coded according to adjusted –log10 p value of a hyper-geometric test. **F.** We tested to what extend the circulating microRNAome defined via the above described methods in mice is comparable to that of humans. To this end we compared samples collected via PAXgene tubes from humans around 40 years of age (38+/- 11 year, n = 19) to samples obtained via the RNAeasy kit from mice at 12 month of age, since 12-month of age in mice is believed to relate to 40 years of age in humans (Dutta S, 2016). MicroRNAs having at least 5 reads in 50% of the samples were considered for the comparative analysis. Our data reveals that the majority of the microRNAs detected in humans PAXgene blood samples is also observed in mice. Even more important is the fact that the corresponding expression levels of the circulating human and mouse microRNAome were significantly correlated (r = 0.65, p = 4.9e−14).

Appendix Fig. S2

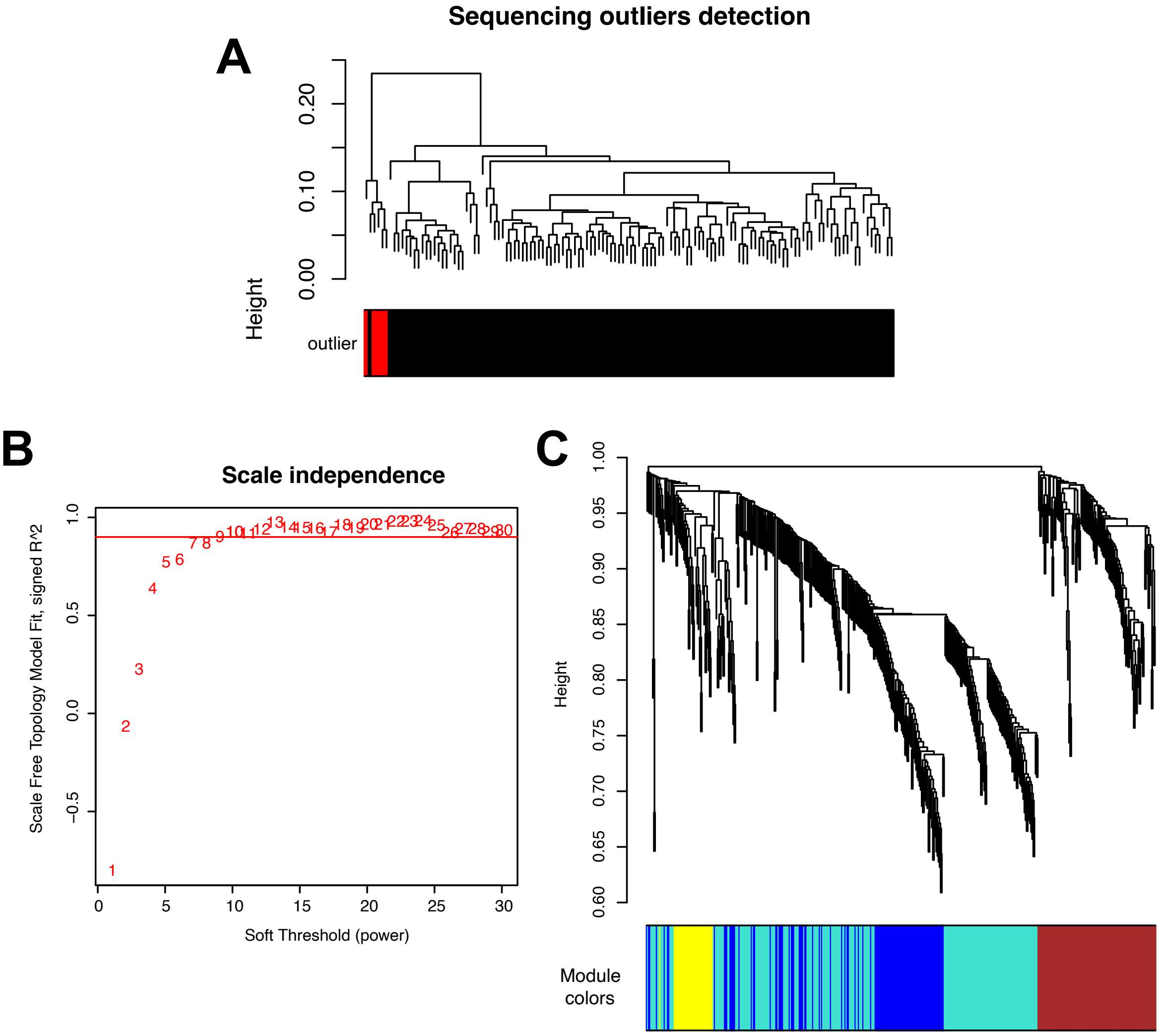

Appendix Fig. S2: WGCNA analysis on circulating microRNAs in young healthy humans. **A.** Outliers were determined using sample clustering method and removed from downstream analyses. Red color denotes the outlying samples. **B.** Soft-threshold power β selection for WGCNA. The scale-free fit index of network topology was determined for various soft-thresholding powers (β). A soft threshold power (β) of 9 was chosen based on approximate scale-free topology to highlight strong correlations (R^2 =0.90). **C.** Cluster dendrogram of co-expressed microRNAs based on topological overlap along with the assigned co-expressed module color. Four co-expression modules were constructed with four different colors. The leaves and height of the tree represent the microRNAs and closeness of individual microRNAs respectively.

Appendix Fig. S3

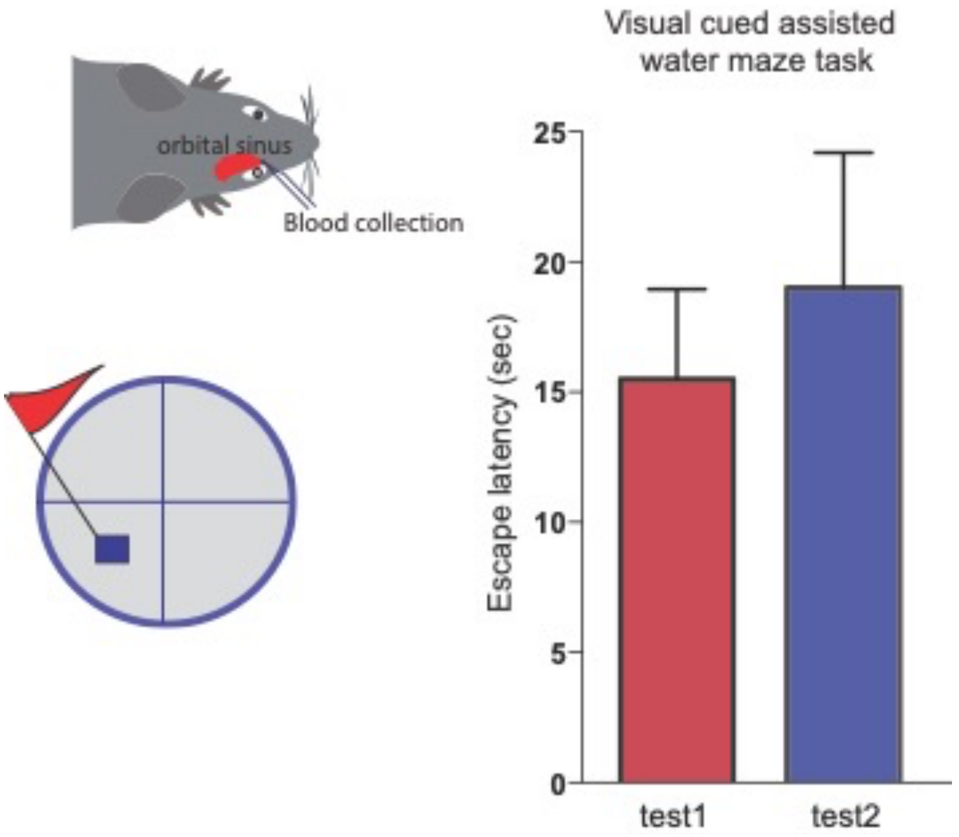

Appendix Fig. S3:: Longitudinal blood collection does not affect visual learning in mice.
Blood was collected from anesthetized mouse via the retro-orbital sinus at the age of 12, 13.5, 15 and 16.5 month of age (See Fig 2A). Alternate eyes were used during blood collection at different time points. A visual cue assisted probe test (schematically indicated in the lower left panel) was performed to check visual performance of mice after habituation training and blood collection at 12 months (test 1, n=10) and after finishing probe test at 16.5 months (test 2, n=10). In both test mice are able to see the platform and thus do not need to rely on spatial reference memory to find the platform. Compromised vision would impair the performance in this test. There is no significant difference in performance (paired t-test, two-tailed, p value 0.59) between two tests. Error bar indicates mean ± standard error mean (SEM).

Appendix Fig. S4

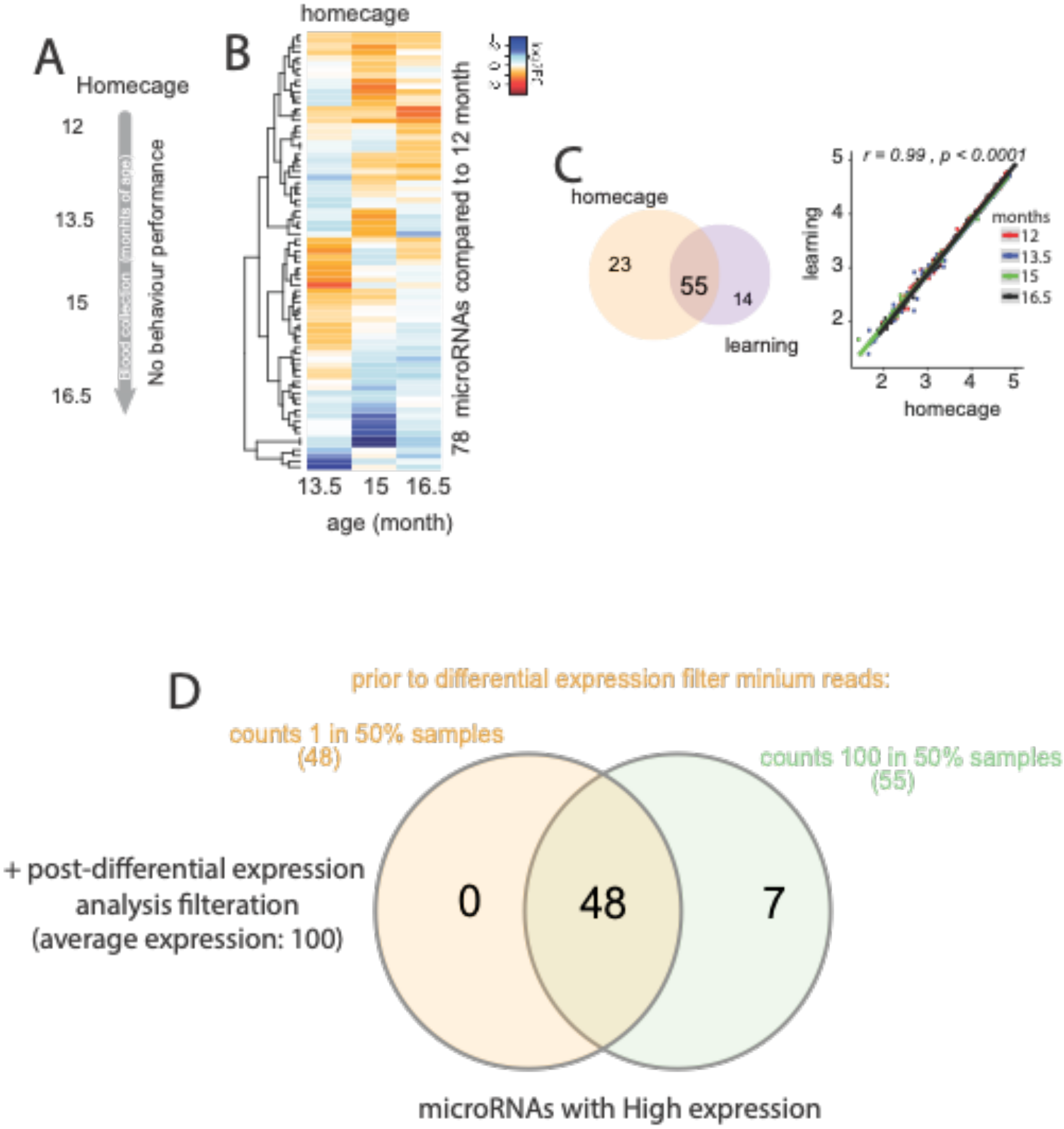

Appendix Fig. S4: Age-associated changes in circulating microRNA in a longitudinal mouse study. A. Experimental design. Paralleling the experiment depicted in Fig 2A, blood was collected from mice at 12, 13.5, 15 and 16.5 month of age and subjected to smallRNA sequencing. These mice were, however, not exposed to water maze testing and are therefore named “homecage” control group. We reasoned that this group would be important to control for microRNAs potentially regulated as a consequence of the water maze training procedure. **B.** Heat map showing 78 differentially expressed microRNAs in aging mice of the “homecage group”. The data shows mircoRNA expression always in comparison to the data at 12-month of age. **C.** Left panel: Venn diagram comparing the microRNAs differentially regulated in the “learning group” shown in Fig 2A and the “home cage control group” shown in panel B. 55 circulating microRNA are regulated during aging and are not affected by the training procedure.

Right panel: Correlation analysis showing that the expression levels of 55 microRNA commonly regulated during aging in mice that were exposed to water maze training (see Fig 2; named here "learning" group) and "home cage" groups show similar expression levels. **D.** Comparison of differential expression analysis applying different filtering thresholds. For the data generated in panel (C) we filtered microRNAs having minimum 100 reads in 50% samples prior to differential expression analysis (approach 1) and found 55 common "age-related" microRNAs between homecage and learning group. As an alternative approach (approach 2), we have performed differential expression analysis in homecage and learning groups after filtering microRNAs having at least 1 read in 50% samples. After comparison between homecage and learning group based on differential expression analyses results from approach 2, we found 48 common "age related" microRNAs with high expression (average expression 100). Venn diagram displays that these 48 microRNAs from approach 2 are part of those 55 microRNAs based on approach 1. More importantly, miR-146a-5p, miR-148a-3p and miR-181a-5p are among the common microRNAs between two approaches. Full list of 48 microRNAs is summarized in Dataset EV4.

Appendix Fig. S5

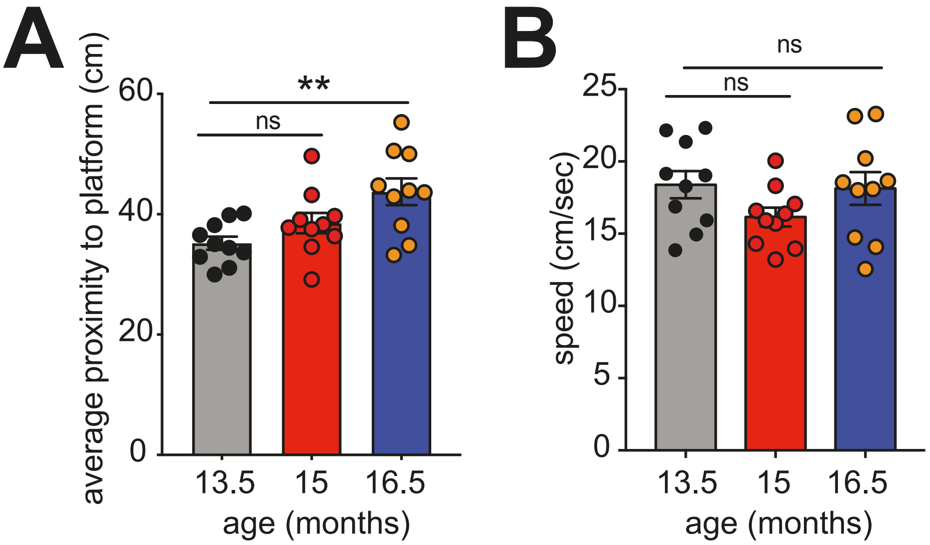

Appendix Fig. S5: Longitudinal analysis of spatial reference memory in aging mice. Aging mice were longitudinally analyzed for spatial reference memory in the Morris water maze paradigm. In addition to the analysis of the various learning strategies during the training (Fig 2), mice were subjected to a memory test at the end of each experiment. During this memory test (probe test) the platform was removed from the pool and the swimming path of the mouse was analyzed. **A.** Depicted is the average proximity to the former platform position during a 60 sec memory test which is a sensitive measure of spatial reference memory. The average proximity to the former platform was significantly increased at 16.5 months compared to 13.5 months (adjusted ***p* value 0.0032), which is indicative of impaired memory retrieval. **B.** Velocity across different age groups. Speed of the mice performing water maze was not statistically different. One-way ANOVA, Error bars indicate mean ± SEM.

Appendix Fig. S6

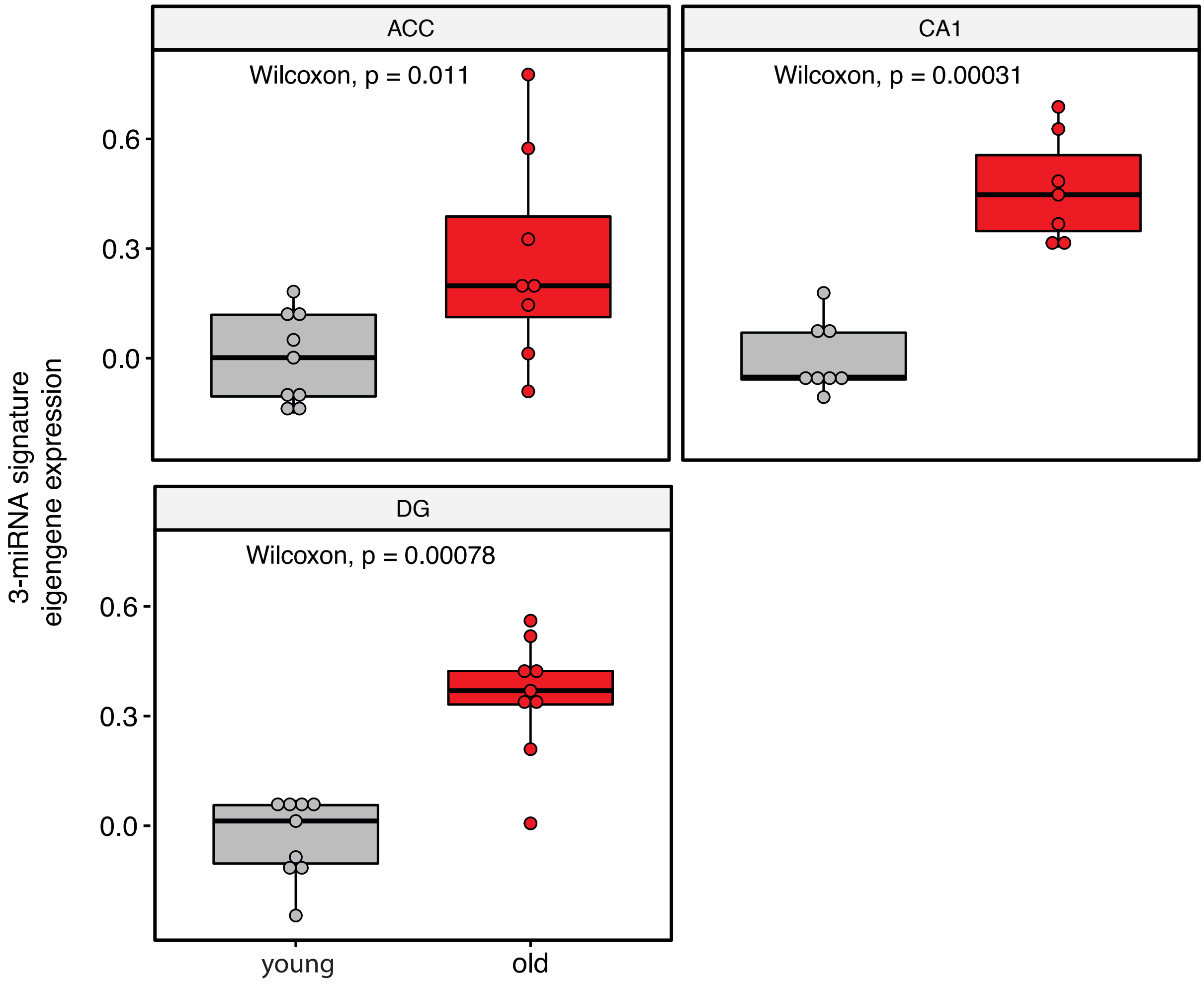

Appendix Fig. S6: Age effect on expression of 3-miRNA in different brain regions. SmallRNAome analyses were performed on different brain sub-regions from 3- (young) and 16.5-months (old) mice (See Fig S8). Co-expression of the 3-microRNA signature is increased at old age in anterior cingulate cortex (ACC), cornu ammonis 1 (CA1) and dentate gyrus (DG). Number of mice (young: 8-9, old: 7-9), Wilcoxon rank sum test. P value is depicted on each panel.

Appendix Fig. S7

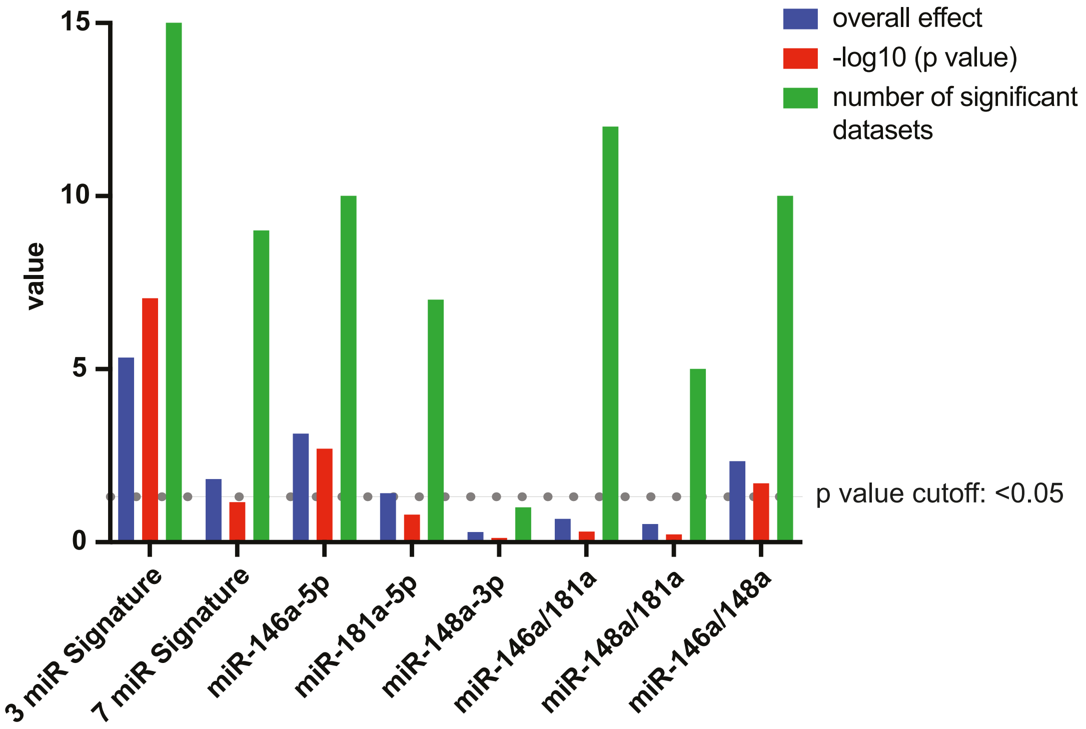

Appendix Fig. S7: Comparison of 3-microRNAs signature to single/dual/seven microRNA signatures from the selected features. The 3-microRNA signature (see also Fig 5I) outperforms the 7-microRNA signature. Additionally, it outperforms the analysis of single microRNAs or the dual combinations. The overall effect was highest for the 3-microRNA signature with high significance. Moreover, the 3-microRNA signature displayed significant differences in all l5 analyzed datasets tested.

Appendix Fig. S8

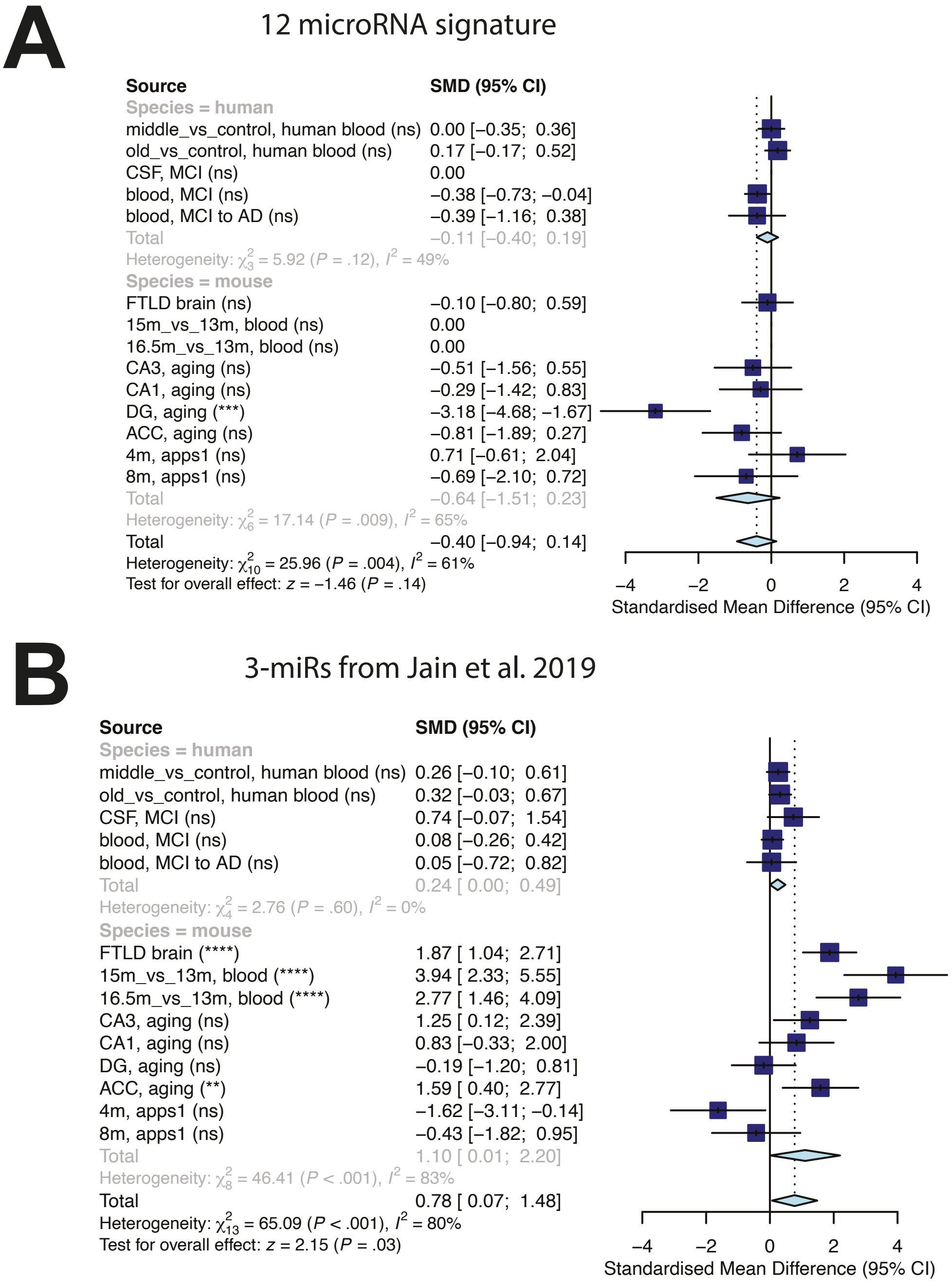

Appendix Fig. S8. Meta-analyses performed using previously described microRNA signatures. Meta analyses in 14 datasets used in Expanded View Fig. 14. for previously described microRNA signatures. **A.** 12-microRNA signature from Leidinger et al. 2013 {Leidinger, 2013} **B.** 3 microRNAs from a previously described CSF based microRNA-piRNA signature (Jain *et al*, 2019). Adjusted p values across studies are summarized in the parentheses next to the study name.

Appendix Fig. S9

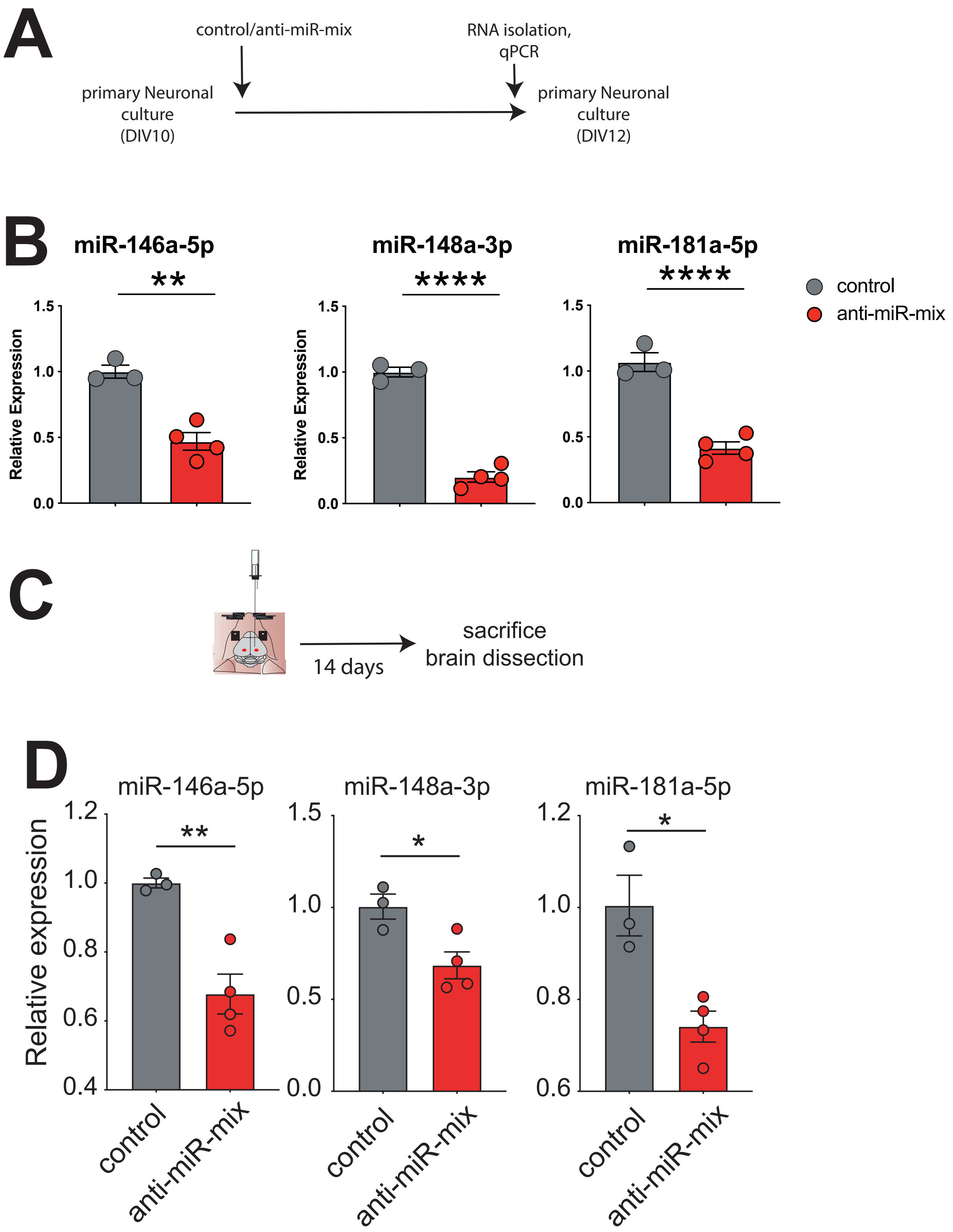

Appendix Fig. S9. The 3-miR inhibitor mix reduces expression level of all three microRNAs *in vitro* and *in vivo*. **A.** Experimental scheme for the experiment in primary cultures. **B.** qPCR analysis showing microRNA expression after inhibitor mix treatment. Note that treatment with the anti-miR-mix significantly reduced the levels of each microRNA. n = 3-4, unpaired t-test, two-tailed, **P<0.01, ****P<0.0001. Error bar indicates mean ± sem. **C.** Experimental scheme for in vivo experiments. An

inhibitor cocktail of the 3 microRNAs (anti-miR-mix) was stereotactically injected into the hippocampus of mice. As control group, mice of the same age were similarly treated with scramble control oligonucleotides. Two weeks post injection mice were sacrificed and hippocampi were isolated for RNA isolation. **D.** Barplots showing qPCR based quantification of (left) miR-146a-5p, (middle) miR-148a-3p and (right) miR-181a-5p. All miRs were significantly reduced in the ani-miR-mix treated group. Number of mice; control = 3, inhibitor-miR mix = 4, unpaired t-test, two-tailed. Error bar = mean ± sem. *P<0.05, **P<0.01

Appendix Fig. S10

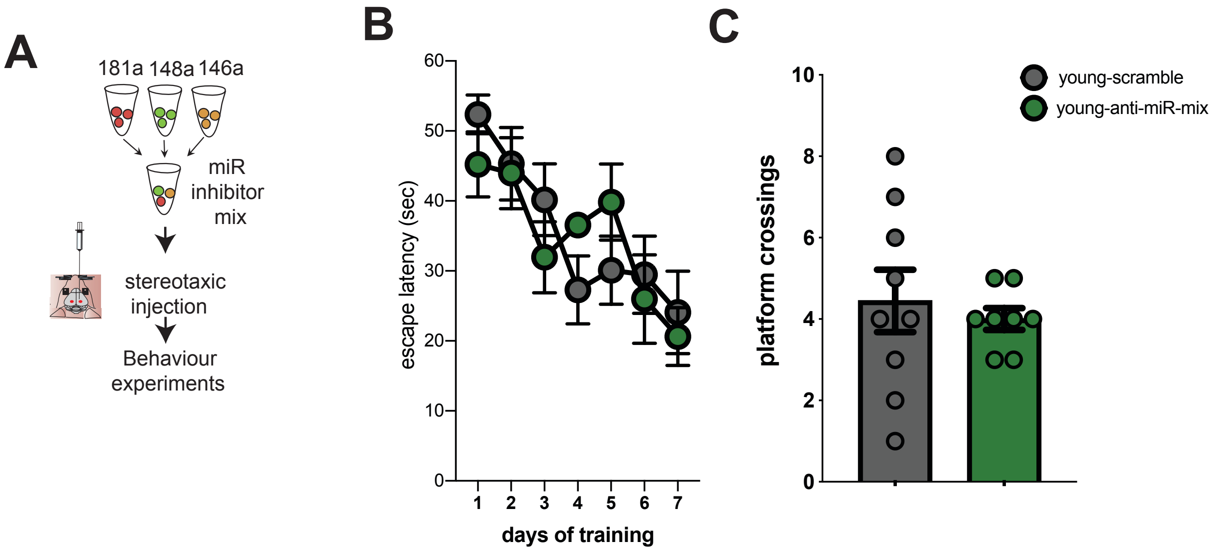

Appendix Fig. S10. The anti-miR mix does not affect water maze performance in wild type mice. A. 3-months old mice were stereotactically injected with anti-miR-mix (young-anti-miR-mix) or scramble control oligonucleotides (young-scramble) and subjected to the water maze pardigm test. Both **(B)** learning and **(C)** memory retrieval performance during the memory test were similar between two groups. Unpaired t-test, two-tailed statistics, N = 8-9/group. Bar plot indicates mean ± sem. Error bar indicates mean ± standard error mean.

Appendix Fig. S11

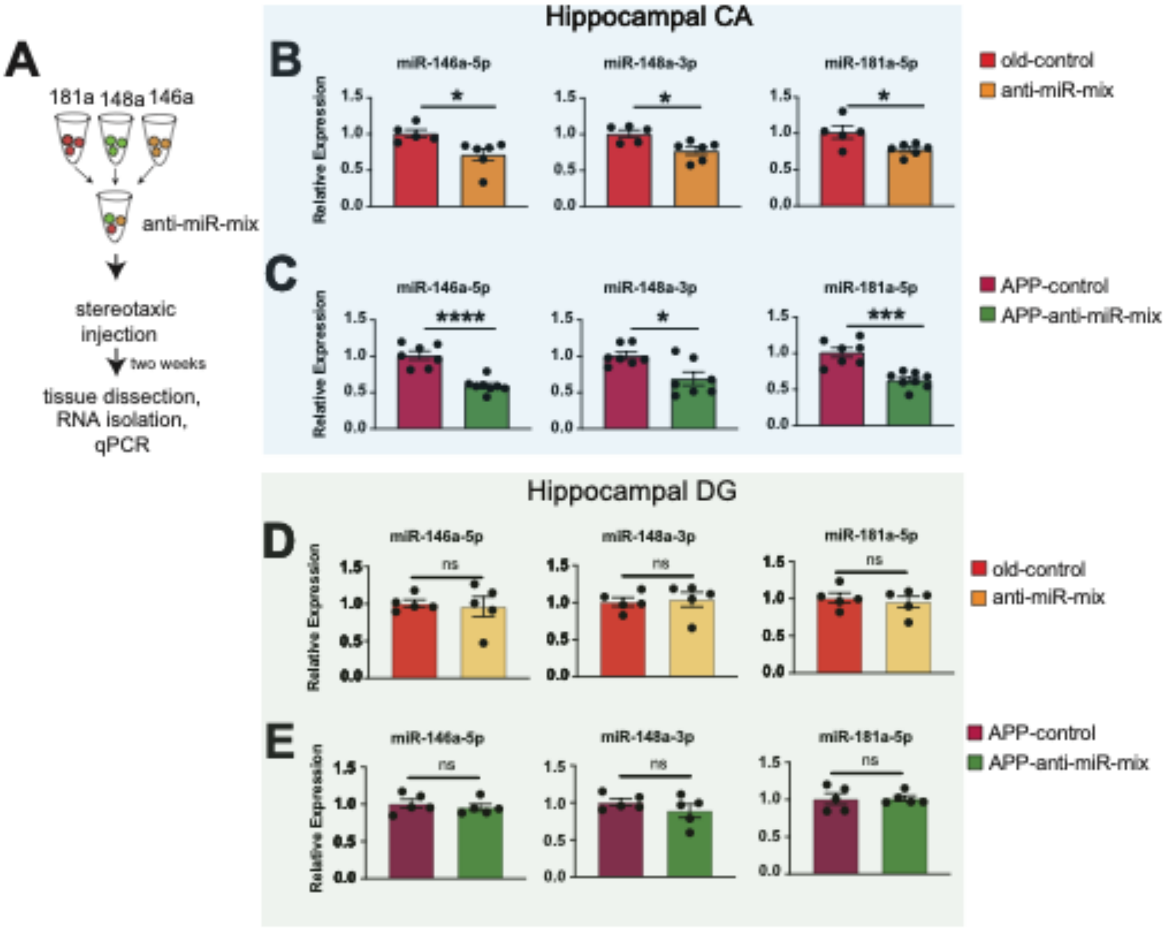

Appendix Fig. S11. The anti-miR mix reduces microRNA expression level at the injected brain region. A. Experimental scheme. **B.** Barplot showing microRNA expression levels in the hippocampal CA region of 16 months old mice injected into the hippocampal CA region with either scramble control oligonucleotides (old-control) or anti-miR-mix (old-anti-miR-mix). N = 5-6. **C.** Barplot showing the expression of the 3 microRNAs in the hippocampal CA region of APP mice injected into the hippocampal CA region with either scramble control oligonucleotides (APP-control) or anti-miR-mix (APP-anti-miR-mix) (n = 7/group). Unpaired t-test, two-tailed. Error bar indicates mean ± sem. *P<0.05, **P<0.01, ***P<0.001, ****P<0.0001. **D and E** are similar to the experiments described in B/C but in this case the hippocampal dentate gyrus (DG) regions was analyzed. Please note the injection into the CA region did not affect miR expression in the DG. N = 5/group Unpaired t-test, two-tailed. Error bar indicates mean ± sem. *P<0.05, **P<0.01, ***P<0.001, ****P<0.0001.

Appendix Fig. S12

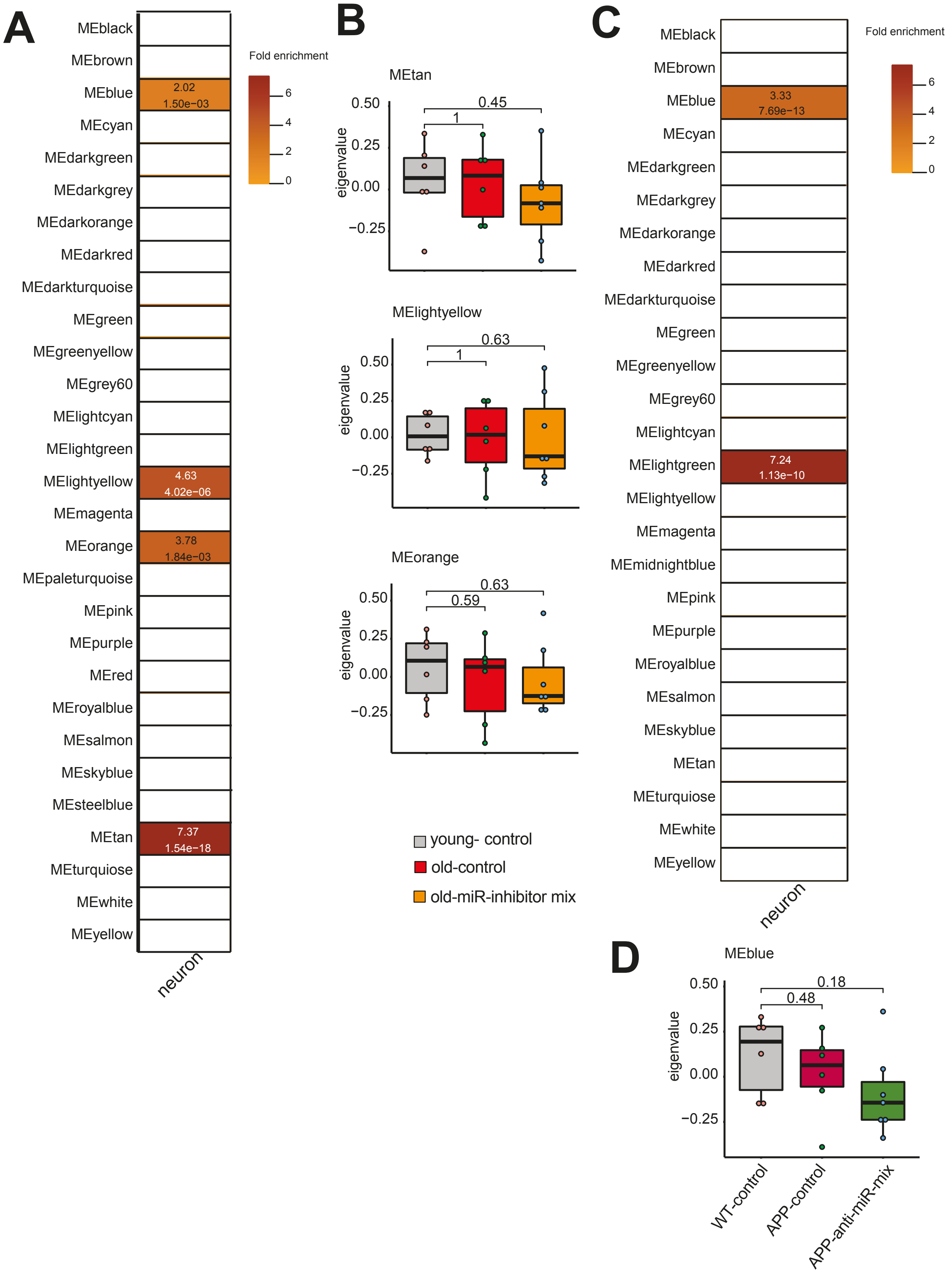

Appendix Fig. S12. Identification of neuronal modules from aging and APPPS1-21 mouse models. Weighted gene co-expression analysis revealed 29 and 26 modules in aging and APPPS1-21mice respectively. Considering a dataset representing neuronal genes from a previous study(Zhang Y,

2014)we performed an overlap analysis of the modules with that data to find which of these modules represent neuronal cluster. Modules with Fold enrichment higher than 1.50 and FDR < 2e-03 were considered as significantly enriched cluster. **A.** MEblue, MElightyellow, MEorange and MEtan modules from aging RNAseq data represent cluster with neuronal genes. **B.** Eigen-expression of MElightyellow, MEorange and MEtan modules among experimental groups. These three clusters did not change in its expression among groups. **C.** Genes belonging in MEblue and MElightgreen modules from APPPS1-21mice significantly overlap with those from neuronal cell type enriched genes. **D**. The changes in Expression of MEblue among groups were not statistically significant. Kruskal-wallis test.

Appendix Fig. S13

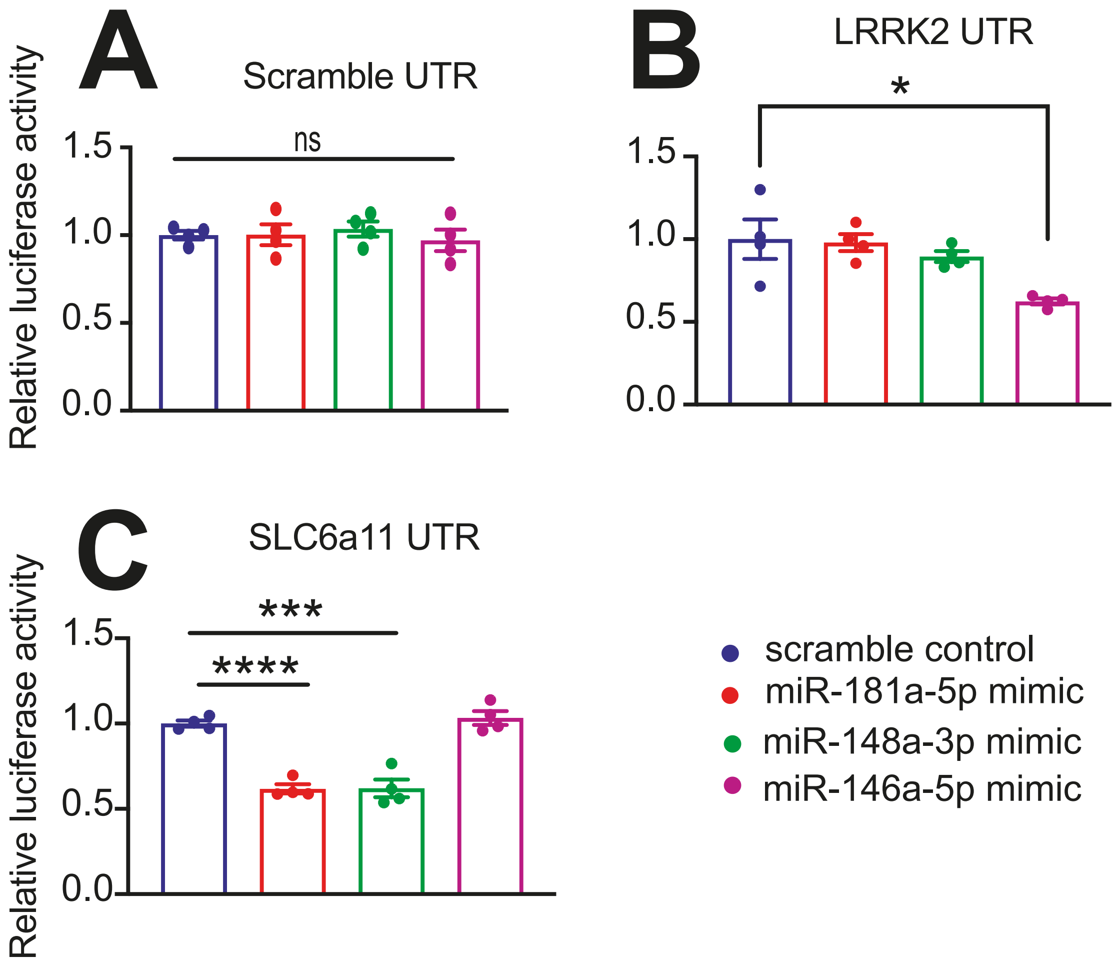

Appendix Fig. S13. Luciferase assay on selected candidate genes. The 3'UTR sequence of LRRK2 and SLC6A11 (mm10) were cloned into pEZX-MT06 Dual-Luciferase miTarget™ vector and downstream to firefly luciferase insert of the vector. Corresponding scrambled sequences were uses as control (scramble-UTR). For a given gene of interest, corresponding vector and mimic or negative control were co-transfected into HEK293-T cells using EndoFectin™ Max Transfection Reagents. After 48 hours of transfection, Firefly and Renilla luciferase activities were measured using a Luc-Pair™ Duo-Luciferase HS Assay Kit. Firefly luciferase activity and Renilla luciferase activity was normalized. **A.** Relative luciferase activity for scramble UTR. No statistical difference was observed among groups. **B.** LRRK2 is a predicted target of miR-146a-5p. Reduced luciferase activity was observed when the LRRK2 3´UTR vector was co-transfected with miR-146a-5p-mimic, while the other microRNAs had no effect (n = 4/group, One-way ANOVA, Dunnett's multiple comparisons test, scramble control vs miR-146a-5p mimic: P = 0.0052). These data show that miR-146a-5p can regulate LRRK2. **C.** SLC6a11 is predicted target gene for both miR-181a-5p and miR-148a-3p. Reduced luciferase activity in SLC6a11 3´ UTR vector - miR-181a-5p and SLC6a11 3´ UTR vector - miR-148a-3p co-transfected cells (n = 4/group, One-way ANOVA, Dunnett's multiple comparisons test, scramble control vs miR-181a-5p mimic: P < 0.0001; scramble control vs miR-148a-3p mimic: P < 0.0001. Error bars on bar plots indicate mean ± standard error mean.

References for Appendix Supplemental figures.

Dutta S SP (2016) Men and mice: Relating their ages. *Life Sci* 1: 244-248

Jain G, Stuendl A, Rao P, Berulava T, Pena Centeno T, Kaurani L, Burkhardt S, Delalle I, Kornhuber J, Hüll M *et al* (2019) A combined miRNA-piRNA signature to detect Alzheimer's disease. *Translational Psychiatry* 9: 250

Zhang Y CK, Sloan SA, Bennett ML, Scholze AR, O'Keeffe S, Phatnani HP, Guarnieri P, Caneda C, Ruderisch N, Deng S, Liddelow SA, Zhang C, Daneman R, Maniatis T, Barres BA, Wu JQ. (2014) An RNA-sequencing transcriptome and splicing database of glia, neurons, and vascular cells of the cerebral cortex. . *J Neurosci* 3: 1929-1947
