# Supplementary material for: A microRNA signature that correlates with cognition and is a target against cognitive decline
Source: EMBO Mol Med. 2021 Oct 11;13(11):e13659. doi: 10.15252/emmm.202013659 (PMC8573587; doi:10.15252/emmm.202013659)
Supplement: Supplementary file 2 — Expanded View Figures PDF [file EMMM-13-e13659-s013.pdf]

Expanded View Figures

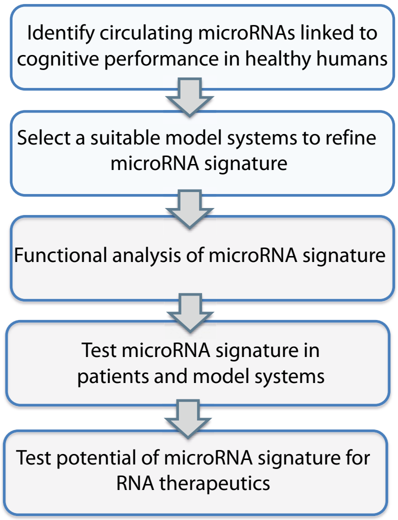

Figure EV1. Experimental approach.

The scheme displays our overall experimental approach and the various filtering steps to identify circulating microRNAs that informs about the cognitive reserve and would allow the early detection of patients at risk for developing cognitive decline. We hypothesized that a promising approach would be to first identify circulating microRNAs that correlate with memory performance in young and healthy humans. Based on the function of such microRNAs, we then thought to employ model systems to further refine these data and develop a microRNA signature that could then be analyzed at the functional level and be eventually tested in patients and disease models. We also planned to test the potential of such a microRNA signature for RNA therapeutics with the aim to ameliorate cognitive decline.

© 2021 The Authors

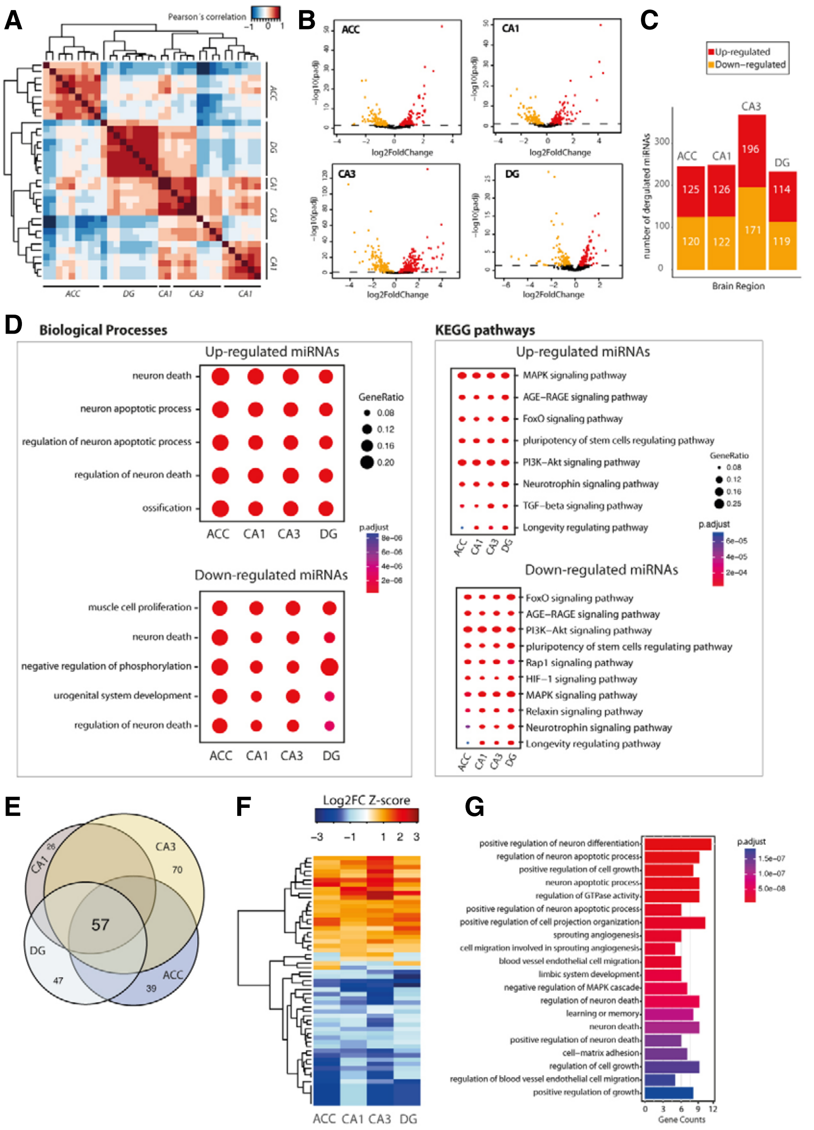

Figure EV2. The microRNAome of learning-related brain regions in the aging mouse brain.

We performed small RNA sequencing of the hippocampal sub-regions CA1, CA3, and dentate gyrus (DG) and the anterior cingulate cortex (ACC) isolated from young (3 months of age) and cognitively impaired old (16.5 months of age) mice.

- A Pearson's coefficient-based correlation followed by unsupervised clustering of all small RNA-seq data from different brain regions (ACC; CA1; CA3; DG) in young mice reveals brain region-specific expression of the microRNAs that were particularly obvious for the ACC versus the hippocampal sub-regions and the DG versus CA1 and CA3. These region-specific differences were, however, mainly attributed to different expression values since the majority of the microRNAs, namely 176 microRNAs, could be detected at reliable levels in all investigated brain regions.
- B Volcano plots showing differential expression of microRNAs in the different brain regions when comparing young versus old mice.
- C Bar plots showing the number of up and downregulated microRNAs in the investigated brain regions.
- D Comparison of gene ontology and functional pathways of experimentally confirmed target genes of the deregulated microRNAs in the investigated brain regions. Pathway is generally linked to neuronal death and longevity pathways.
- E Venn diagram showing that 57 microRNAs are commonly deregulated in the aging brain.
- F Heat map showing hierarchical clustering of the 57 commonly deregulated microRNAs based on Log2 fold change Z-score.
- G Top 20 biological processes affected by the confirmed target genes of the 57 commonly deregulated microRNAs in the aging brain.

© 2021 The Authors

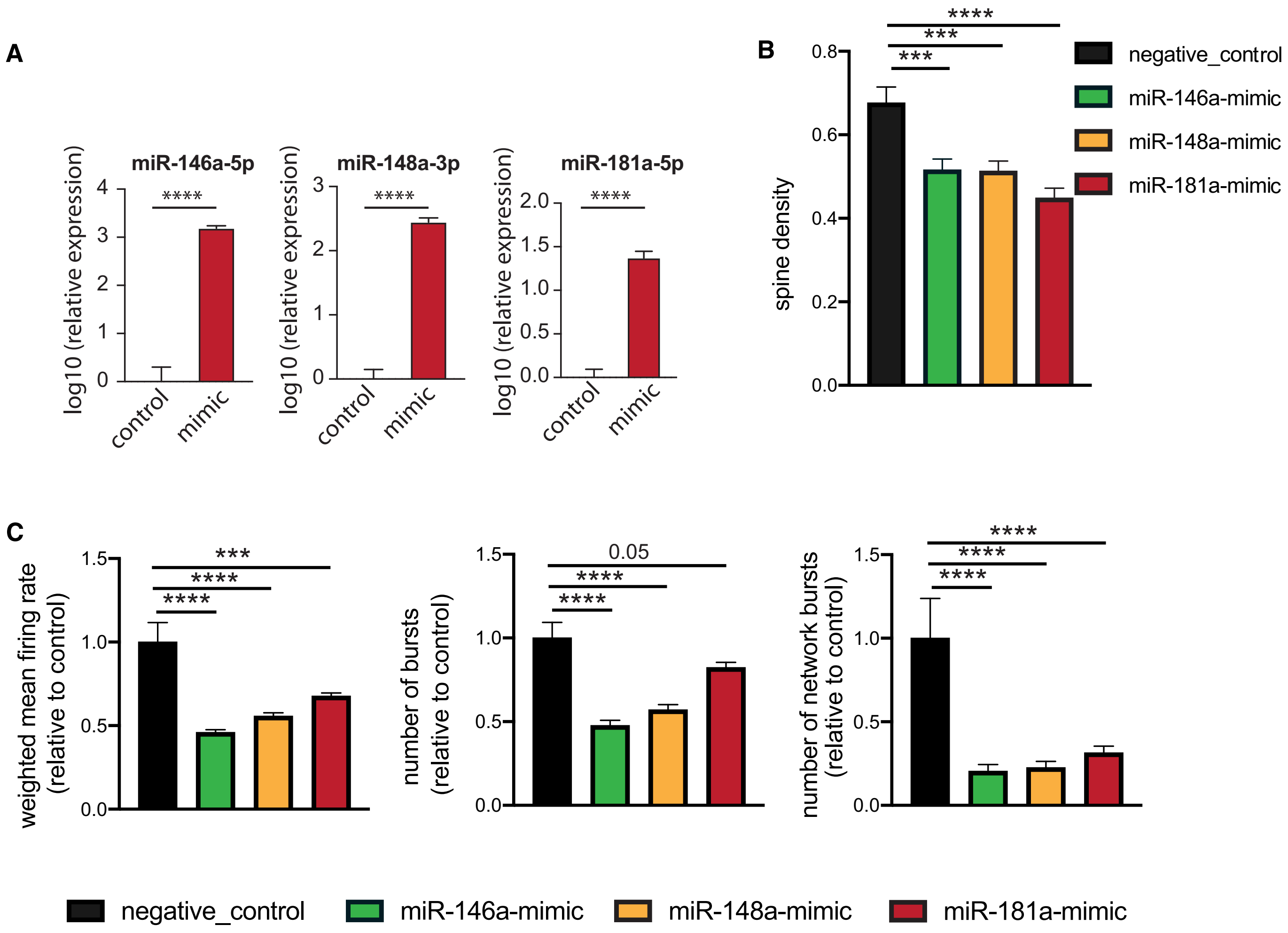

Figure EV3. Effect of individual microRNAs on neuronal functions.

A 3-miR-mix containing (miR-146a-5p, miR-181a-5p, miR-148a-3p mimics) was applied on primary hippocampal neurons at DIV10, and its effect was compared to neurons treated with a scrambled control RNA. After 48 h, cells were prepared for RNA Isolation. qPCR data from isolated RNA reveal increased expression of miR-146a-5p (left), miR-148a-3p (middle), and miR-181a-5p (right). Unpaired *t*-test, two-tailed, $n = 5$/group. ****$P < 0.0001$. Data are normalized to control and log10 scaled. Error bar indicates mean ± SEM.

B Primary hippocampal neurons were transfected with scrambled/individual microRNA mimics at DIV7, and dendritic spines were stained with DiI at DIV10 for dendrite labeling and quantification. Spine density is substantially reduced for mimic-treated primary neurons compared to those treated with scrambled RNA. Barplots showing spine density among groups. $N = 20$ dendritic segments/group, one-way ANOVA, Dunnett's multiple comparisons test, ***$P < 0.001$, ****$P < 0.0001$.

C Hippocampal neurons were cultured in a multielectrode array (MEA) plate equipped with sixteen electrodes. Individual microRNA mimic was applied at DIV7, and a downstream neuronal activity was measured at DIV10 and compared to scrambled control-treated neurons. Weighted mean firing rate, number of bursts, number of network bursts. 6 replicates/group recorded 8× in every 3 h, each record lasting for 10 min. $N = 48$, one-way ANOVA, Dunnett's multiple comparisons test, ***$P < 0.001$, ****$P < 0.0001$. Error bars indicate mean ± SEM.

© 2021 The Authors

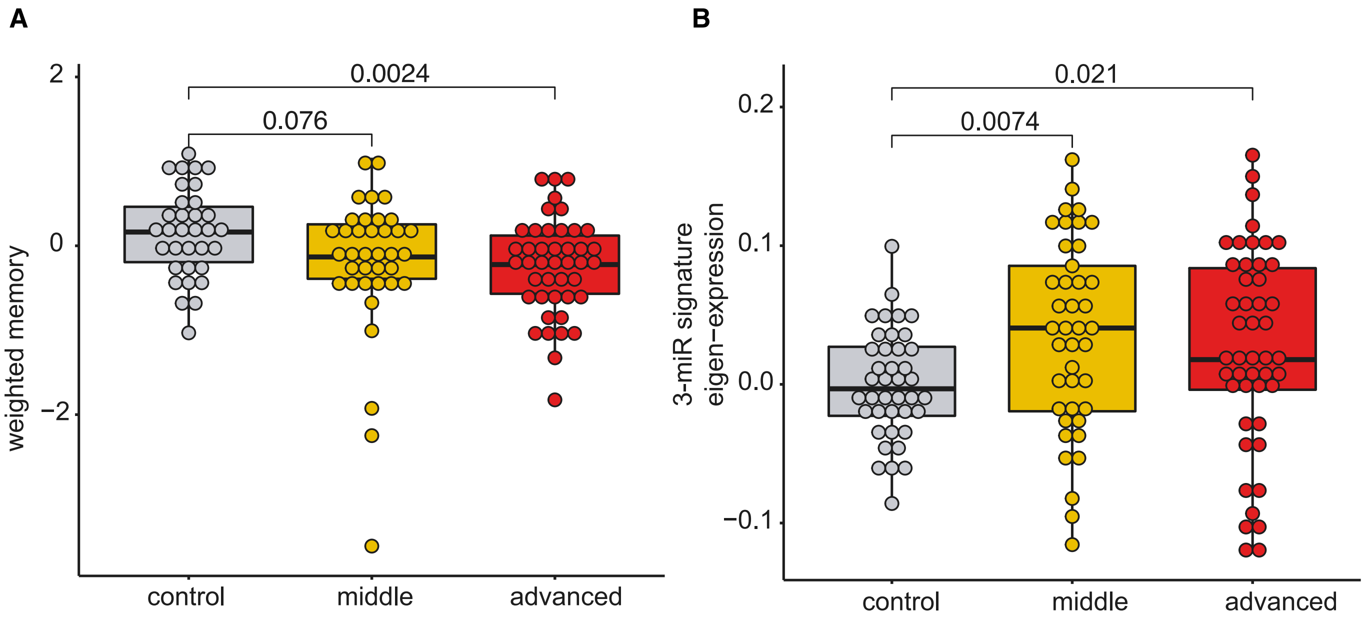

Figure EV4. The 3 microRNA signature changes in aging humans that exhibit cognitive decline.

A Although care has to be taken when comparing data from mice to humans, previous work suggests that 12-month-old mice are comparable to 40-year-old humans and that 1.5 months in a mouse's lifetime approximately reflect 5 years of lifetime in humans (Dutta, 2016). Thus, our longitudinal experiment in mice (see Figs 2 and 3A and B) could be compared best to humans from 40 to 55 years of age. This is in line with previous cross-sectional and longitudinal studies in humans, reporting that impairments of certain cognitive abilities become evident from 40 years of age while after 54 years of age, most cognitive domains significantly decline (Schaie, 1993; Park *et al*, 2002; Hedden & Gabrieli, 2004; Park & Reuter-Lorenz, 2009; Salthouse, 2009; Singh-Manoux *et al*, 2012). Therefore, we recruited healthy individuals aged between 30 and 77 years of age that were subjected to blood collection and the same cognitive phenotyping as in our discovery cohort (See Fig 1). Based on previous data (Schaie, 1993; Park *et al*, 2002; Hedden & Gabrieli, 2004; Park & Reuter-Lorenz, 2009; Salthouse, 2009; Singh-Manoux *et al*, 2012), we divided the individuals into a "control group" that is expected to exhibit full cognitive functioning (30–40 years of age), a "middle-age group" (41–53 years of age) that is expected to show very mild signs of cognitive decline, and an "advanced age group" (54–77 years of age) that are expected to display significant cognitive decline. Despite the fact that older individuals exhibit cognitive impairments, we like to reiterate that all of these individuals were healthy and none of them suffered from mild cognitive impairment or dementia. We confirmed a non-significant trend for reduced cognition in the "middle-age group" that became significant in the "advanced age group".

B Next, we performed small RNA sequencing from all collected samples and analyzed the microRNA expression. Similar to the data obtained in mice, co-expression of the 3-miR signature was significantly increased already in the "middle-age group" and plateaued in "advanced age group" when compared to individuals 30–40 years of age. Although these data are cross-sectional, they are in line with our observation from the longitudinal study in mice and suggest that also in humans, expression of the 3-microRNA signature might increase in blood prior to the detection of significant cognitive impairment, at least in the employed experimental design. Kruskal–Wallis test, n = 40–47 human subjects.

Figure EV5. Comparison of the 3-microRNA signature in blood with established CSF AD biomarker within the same control and MCI patients.

Please note that expression data of the CSF biomarker were not available for all the samples used in Fig 5B. Therefore, the comparative analysis of the blood 3-microRNA signature and the CSF biomarker was performed on control and MCI patients for which all datasets were available; control = 24, MCI = 52.

A–E Eigenvalue of 3-microRNA signature in blood is significantly increased in MCI patients compared with controls. Levels of (B) Aβ38 and (C) Aβ40 did not change significantly between patients and control subjects. Both (D) Aβ42 alone and (E) Aβ42/40 ratio were significantly different between control and MCI patients.

F There was no statistically significant difference in the levels of phosphorylated Tau 181 when comparing MCI patient versus controls. Since Tau could be considered a marker for neurodegeneration, it is not unexpected that changes in Aβ precede changes in Tau pathology. Unpaired t-tests, two-tailed. These data suggest that the 3-microRNA signature is comparable to more invasive established AD biomarker.

▶

 © 2021 The Authors

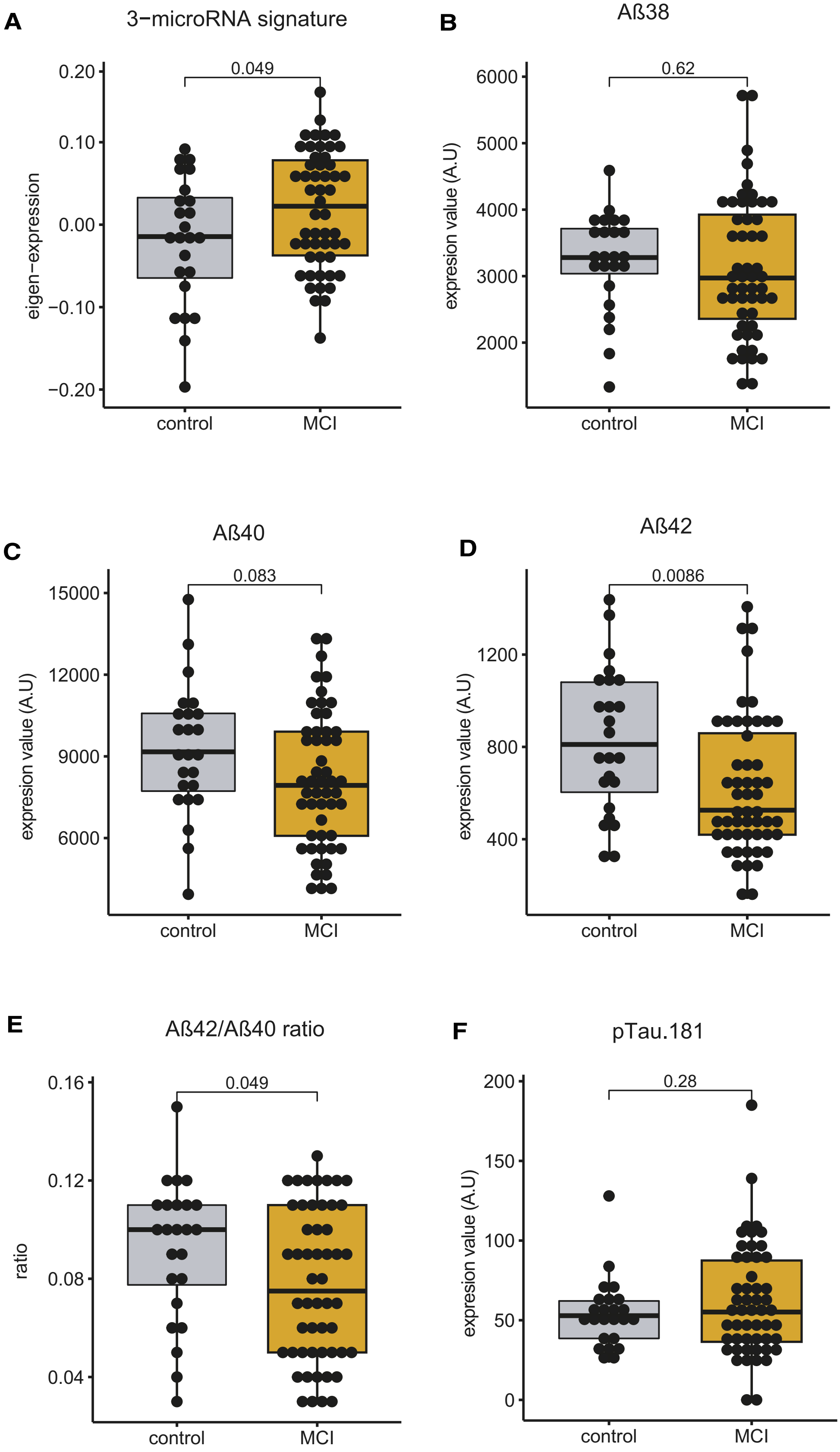
A
3−microRNA signature
0.049
eigen−expression
0.20
0.10
0.00
−0.10
−0.20
control
MCI
B
Aß38
0.62
expresion value (A.U)
6000
5000
4000
3000
2000
control
MCI
C
Aß40
0.083
expresion value (A.U)
15000
12000
9000
6000
control
MCI
D
Aß42
0.0086
expresion value (A.U)
1200
800
400
control
MCI
E
Aß42/Aß40 ratio
0.049
ratio
0.16
0.12
0.08
0.04
control
MCI
F
pTau.181
0.28
expresion value (A.U)
200
150
100
50
0
control
MCI

Figure EV5.
