# Supplementary material for: A microRNA signature that correlates with cognition and is a target against cognitive decline
Source: EMBO Mol Med. 2021 Oct 11;13(11):e13659. doi: 10.15252/emmm.202013659 (PMC8573587; doi:10.15252/emmm.202013659)
Supplement: Supplementary file 20 [file EMMM-13-e13659-s006.pdf]

EMBO Molecular Medicine

A microRNA-signature that correlates with cognition and is a target against cognitive decline

Rezaul Islam, Tea Berulava, Urs Heilbronner, Monika Budde, Tonatiuh Pena-Centeno, Vakhtang Elerdashvili, Maria Zafeiriou, Eva Benito, Sinem Sertel, Fanny Senner, Janos Kalman, Susanne Burkhardt, Mohammad Sakib, Cemil Kerimoglu, Heike Bickeböller, Claudia Bartels, Frederic Brosseron, Katharina Buerger, Nicoleta Cosma, Klaus Fließbach, Michael Heneka, Daniel Janowitz, Ingo Kilimann, Luca Kleinedam, Christoph Laske, Coraline Metzger, Mattias Munk, Robert Perneczky, Oliver Peters, Josef Priller, Boris Rauchmann, Nina Roy, Anja Schneider, Annika Spottke, Eike Spruth, Stefan Teipel, Maike Tscheuschler, Michael Wagner, Jens Wiltfang, Emrah Düzel, Frank Jessen, Silvio Rizzoli, Wolfram Zimmermann, Thomas Schulze, Peter Falkai, Farahnaz Sananbenesi, André Fischer, Oliver Wirths, Anne Oepen, and Maria Goldberg
DOI: 10.15252/emmm.202013659

Corresponding author: André Fischer (A.Fischer@eni-g.de)

Review Timeline:

Submission Date:	28th Oct 20
Editorial Decision:	19th Jan 21
Revision Received:	6th Aug 21
Editorial Decision:	16th Aug 21
Revision Received:	23rd Aug 21
Accepted:	24th Aug 21

Editor: Jingyi Hou

Transaction Report:

(Note: With the exception of the correction of typographical or spelling errors that could be a source of ambiguity, letters and reports are not edited. Depending on transfer agreements, referee reports obtained elsewhere may or may not be included in this compilation. Referee reports are anonymous unless the Referee chooses to sign their reports.)

1st Editorial Decision 19th Jan 2021

19th Jan 2021

Thank you for the submission of your manuscript to EMBO Molecular Medicine. We have finally received feedback from the three referees whom we asked to evaluate your manuscript. As you will see from the reports below, the referees acknowledge the potential interest of the study and the importance of the topic. However, they also raise substantial concerns about your work, which should be convincingly addressed in a major revision of the present manuscript.

Without reiterating all the points raised in the reviews below, some of the more substantial issues are the following:

1. The point #33 raised by Referee #2 is of particular importance and needs to be satisfactorily addressed.

2. The sex difference should be taken into consideration in the mouse experiments, as Referees #1 and #2 pointed out. Referee #2 also requests a separate analysis of male and female humans (instead of the tests being done together and the contribution of each gender being corrected for), which we would encourage you to address.

3. Referee #2 mentioned that the provision and sharing of computer code is not optimal (points # 21-24). Please revise and improve according to this referee's suggestions.

4. The referees raised a series of issues regarding the computational analysis and statistics, which need to be carefully addressed and clarified.
Referee# 2 also performed a re-analysis on some of your data and the resulting figures are bundled together in a single pdf file called *islam_et_al_review_01_21* (please see attached). During our pre-decision cross-commenting process (in which the reviewers are given the chance to make additional comments, including on each other's reports), Referee #2 added "in our assessment of the methodical and statistical basis of the authors' reasoning, we have come to the conclusion that the application of bioinformatics methods is not up to the standards of rigorous experimentation, which is a pity as it is of utmost importance in such a consequential manuscript. Our doubts upon retracing their analyses step-by-step were considerable to the point of stopping examination of their code prematurely, at figure 3, as the statistical basis of the authors' deductions seems fundamentally flawed (please compare in particular our points #26-34). As such, we would recommend a thorough re-analysis and more transparent presentation of all of the manuscript's statistical foundations." In light of the comment of Referee #2, particular attention should be paid to addressing the issues related to bioinformatic analyses, statistics and data/code presentation.

All other issues raised by the referees need to be satisfactorily addressed as well. We would welcome the submission of a revised version within three months for further consideration. Please note that EMBO Molecular Medicine strongly supports a single round of revision and that, as acceptance or rejection of the manuscript will depend on another round of review, your responses should be as complete as possible. Please feel free to contact me in case you would like to discuss in further detail any of the issues raised.

EMBO Molecular Medicine has a "scooping protection" policy, whereby similar findings that are published by others during review or revision are not a criterion for rejection. Should you decide to submit a revised version, I do ask that you get in touch after three months if you have not completed it, to update us on the status.

We are aware that many laboratories cannot function at full efficiency during the current COVID-19/SARS-CoV-2 pandemic and have therefore extended our "scooping protection policy" to cover the period required for a full revision to address the experimental issues. Please let me know should you need additional time, and also if you see a paper with related content published elsewhere.

Please read below for important editorial formatting and consult our author's guidelines for proper formatting of your revised article for EMBO Molecular Medicine.

I look forward to receiving your revised manuscript.

Kind regards,
Jingyi

Jingyi Hou
Editor
EMBO Molecular Medicine

*** Instructions to submit your revised manuscript ***

** PLEASE NOTE ** As part of the EMBO Publications transparent editorial process initiative (see our Editorial at https://www.embopress.org/doi/pdf/10.1002/emmm.201000094), EMBO Molecular Medicine will publish online a Review Process File to accompany accepted manuscripts.

In the event of acceptance, this file will be published in conjunction with your paper and will include the anonymous referee reports, your point-by-point response and all pertinent correspondence relating to the manuscript. If you do NOT want this file to be published, please inform the editorial office at contact@embomolmed.org.

To submit your manuscript, please follow this link:

Link Not Available

When submitting your revised manuscript, please include:

1) a .docx formatted version of the manuscript text (including Figure legends and tables). Please make sure that the changes are highlighted to be clearly visible to referees and editors alike.

2) separate figure files*

3) supplemental information as Expanded View and/or Appendix. Please carefully check the authors guidelines for formatting Expanded view and Appendix figures and tables at https://www.embopress.org/page/journal/17574684/authorguide#expandedview

4) a letter INCLUDING the reviewers' reports and your detailed responses to their comments (as Word file)

Also, and to save some time should your paper be accepted, please read below for additional information regarding some features of our research articles:

5) The paper explained: EMBO Molecular Medicine articles are accompanied by a summary of the articles to emphasize the major findings in the paper and their medical implications for the non-specialist reader. Please provide a draft summary of your article highlighting
- the medical issue you are addressing,
- the results obtained and
- their clinical impact.

This may be edited to ensure that readers understand the significance and context of the research. Please refer to any of our published articles for an example.

6) For more information: There is space at the end of each article to list relevant web links for further consultation by our readers. Could you identify some relevant ones and provide such information as well? Some examples are patient associations, relevant databases, OMIM/proteins/genes links, author's websites, etc...

7) Author contributions: the contribution of every author must be detailed in a separate section (before the acknowledgments).

8) EMBO Molecular Medicine now requires a complete author checklist (https://www.embopress.org/page/journal/17574684/authorguide) to be submitted with all revised manuscripts. Please use the checklist as a guideline for the sort of information we need WITHIN the manuscript as well as in the checklist. This is particularly important for animal reporting, antibody dilutions (missing) and exact p-values and n that should be indicated instead of a range.

9) Every published paper now includes a 'Synopsis' to further enhance discoverability. Synopses are displayed on the journal webpage and are freely accessible to all readers. They include a short stand first (maximum of 300 characters, including space) as well as 2-5 one sentence bullet points that summarise the paper. Please write the bullet points to summarise the key NEW findings. They should be designed to be complementary to the abstract - i.e. not repeat the same text. We encourage inclusion of key acronyms and quantitative information (maximum of 30 words / bullet point). Please use the passive voice. Please attach these in a separate file or send them by email, we will incorporate them accordingly.

You are also welcome to suggest a striking image or visual abstract to illustrate your article. If you do please provide a jpeg file 550 px-wide x 400-px high.

10) A Conflict of Interest statement should be provided in the main text

11) Please note that we now mandate that all corresponding authors list an ORCID digital identifier. This takes <90 seconds to complete. We encourage all authors to supply an ORCID identifier, which will be linked to their name for unambiguous name identification.

Currently, our records indicate that the ORCID for your account is 0000-0001-8546-1161.

Please click the link below to modify this ORCID:
Link Not Available

12) The system will prompt you to fill in your funding and payment information. This will allow Wiley to send you a quote for the article processing charge (APC) in case of acceptance. This quote takes into account any reduction or fee waivers that you may be eligible for. Authors do not need to pay any fees before their manuscript is accepted and transferred to our publisher.

*Additional important information regarding Figures

Each figure should be given in a separate file and should have the following resolution:
Graphs 800-1,200 DPI
Photos 400-800 DPI
Colour (only CMYK) 300-400 DPI"

Figures are not edited by the production team. All lettering should be the same size and style; figure panels should be indicated by capital letters (A, B, C etc). Gridlines are not allowed except for log plots. Figures should be numbered in the order of their appearance in the text with Arabic numerals. Each Figure must have a separate legend and a caption is needed for each panel.

*Additional important information regarding figures and illustrations can be found at
https://bit.ly/EMBOPressFigurePreparationGuideline

***** Reviewer's comments *****

Referee #1 (Remarks for Author):

In the present manuscript, Fischer and colleagues report the identification of three circulating microRNAs in the blood whose expression levels inform about the cognitive status of human subjects. Furthermore, they provide data from rodents suggesting a role of these microRNAs in synaptogenesis and long-term memory formation. The microRNA signature correlates with aging-dependent cognitive decline, raising the possibility that expression of these microRNAs could be used as a prognostic marker for dementia.
microRNAs have been recently associated with the control of synaptogenesis and memory in rodent studies, making them interesting candidates for the diagnosis and therapy of neurological diseases characterized by memory decline, such as neurodegeneration. However, microRNAs have not yet been firmly established as biomarkers or therapeutic targets in these conditions. An integrative approach like the one presented here is timely and could represent an important step towards establishing microRNAs for clinical application in the diagnosis and therapy of dementia.

Overall, the manuscript is well-written and the findings are mostly supported by the presented data. A particular strength of the manuscript is the robust statistical assessment of microRNA expression in multiple transcriptomic datasets from human and mice, including the integration of already published data from other groups. Moreover, the functional and mechanistic follow-up of the candidate microRNAs distinguishes this study from the wealth of already published miRNA profiling papers in the field of neurodegeneration.
One shortcoming of the study is the limited mechanistic insight with regard to the function of microRNA candidates. Previous studies had already identified a role for miR-146 and miR-181 in memory decline (e.g. Ansari et al., Neurobiology of Aging 2019; Rodriguez et al., Aging Cell 2020 (this paper unfortunately was not cited)), and miR-181 had been shown to control dendritic spine development by regulating the expression of GluA2 (Saba et al., MCB 2012). Moreover, as outlined below in more detail, some of the functional experiments lack important controls and are underpowered, preventing more definitive conclusions at this time.
Taken together, the authors should revise their manuscript according to my suggestions below before it should be considered for publication in EMBO Mol. Med.

Major concerns:
- In the functional experiments provided in Fig. 4, 6 and 7, the authors employ synthetic microRNA mimics or anti-miRs to manipulate microRNA candidates in primary neurons and in the mouse brain in vivo. It is imperative to demonstrate that the treatments actually lead to the expected effects, e.g. microRNA overexpression or inhibition in the respective systems. This could for example be achieved by so-called microRNA sensor assays which monitor microRNA activity via the expression of fluorescent reporter genes coupled to the cognate microRNA binding sites. Alternatively, microRNA levels (if affected) could be measured by qPCR or FISH. With regard to the in vivo injections (Fig. 6), it would in addition be important to monitor the spreading and stability of the anti-miRs in the tissue, e.g. by using fluorescently labeled oligonucleotides.
- Behavioral experiments presented in Fig. 6 are largely underpowered, with n<10 animals for most experimental conditions. It would therefore be important to replicate these findings with an independent, similarly sized cohort of animals. In addition, a statistical assessment of the behavioural data taking into consideration the sex of the mice has to be provided.
- The design of the anti-miR experiments is missing an important control (anti-miR treatment in wt mice), which is not considered good scientific practice. These experiments should follow a classical 2x2 design (2 genotypes, 2 treatments). Data should be assessed by a 2-way ANOVA following appropriate post-hoc tests. Data variance should be reported by standard deviation instead of standard error of the mean.

More minor concerns:
- miR-181 has been previously shown to negatively regulate dendritic spine development (Saba et al., 2012). Therefore, it would be very informative to disentangle the contribution of the individual microRNA mimics to the observed reductions in spine density and length in Fig. 4. This could be addressed by transfecting individual miRNA mimics followed by spine analysis and electrophysiological recordings.
- Little is known regarding the cell-type specific expression of the microRNA candidates. There is some data suggesting a preferential expression of miR-146 in microglia, which complicates the interpretation of the functional data in neurons. Therefore, the authors should preform FISH analysis to assess the cellular localization of the studied microRNA candidates in their mixed primary neuronal cultures and hippocampal slices.
- In Fig. 7, the authors who a rescue of the expression of specific synaptic genes in old or APP-transgenic mice by the injection of the anti-miR mix. Are these genes direct targets of the candidates miRNAs? If so, can the authors provide some target validation experiments, e.g. using

luciferase reporter gene assays. Furthermore, do the 3'UTRs of genes upregulated by the anti-miR mix injection display an enrichment of the respective microRNA binding sites compared to the sequencing background? Such an analysis could also be used as an alternative validation of the effectiveness of the anti-miR injections.

Referee #2 (Remarks for Author):

This highly ambitious study, submitted by 49 co-authors from 31 different research groups and representing very different disciplines in Germany, the UK, and Portugal aims to discover the molecular controllers of the known aging-related decline in memory processes and develop a novel RNA-therapeutics approach to attenuate this process.

Briefly, the authors used PAXgene columns-based RNA-sequencing of nucleated blood cells' short transcripts to identify a translationally valuable 'signature' of three different blood cells-derived microRNAs (miRs) which they report can reflect aging-related changes in the elaboratively studied cognitive capacity of both apparently healthy humans and laboratory mice by means of its eigenexpression, based on accompanying analyses of detailed species-specific cognitive tests. They further present evidence demonstrating that the currently discovered 3-miR signature is superior to miR sets discovered by other researchers and is supported by analyzing multiple independent web-available datasets deposited by others.

Furthermore, this manuscript presents experimental interference studies of the impact of antisense oligonucleotide-based suppressed expression of the identified three blood miRs when intrathecally injected into the hippocampus of model mice, or added to cultured cells, multielectrode electrophysiology-amenable cell plates and brain organoids; which is taken to support a potential therapeutic utility for retrieval of improved cognitive performance following simultaneous suppression of the expression of these three miRs in vitro and in vivo, possibly even in Alzheimer's disease patients as reflected in the employed model mice.

The topic of this study is of utmost importance and translational relevance, especially in these days when RNA-therapeutics rapidly becomes an innovative reality. Also, the presented body of work is unusually multi-disciplinary and reflects numerous state-of-the-art technologies the combined impact of which is essential for the success of this approach. While both the topic and these technologies are strength points of this study, these same issues call for especially careful presentation of the approach and the outcome, errors in which may hamper the clarity and reliability of the presented message. Responses to the queries below and the corresponding revision work may help.

Conceptual queries

1. The implicated pan-species impact of the identified miRs is highly surprising, especially considering that one third of the human-expressed miRs are primate-specific, and could not be detected in mice. The presented findings may therefore be interpreted as implying that aging-dependent cognitive decline is common to all mammals and that it is evolutionarily conserved, which is utterly surprising. This issue needs to be referred to.

2. Likewise, it is surprising to learn that the authors kept seeking data which support their working hypothesis (line 308): in exploratory research, one should try to CHALLENGE one's theory, rather than making efforts to support it.

3. The abstract correctly highlights the need for simple and low-cost therapeutics; but repeated preventive treatment by intrathecal injection of three oligonucleotide mixtures into the brain of apparently healthy individuals is neither simple nor cheap. Please comment on the approach and the timeline predicted to last between treatments.

4. That the identified 3 miRs signature is derived from nucleated blood cells raises the question of why their cumulative antisense suppression in the brain is effective; an in-depth explanation of the identification of this signature and the predicted cellular and molecular route involved and the shared impact on blood and brain cells may help to convince the readers, especially if accompanied by a comparison to the other smRNA signature proposed by these authors in their 2019 article, where they identified a three-miRNA-three-piRNA-signature for AD, which includes miR-27a-3p, miR-30a-5p, and miR-34c; three entirely different miRNAs than the ones described in their current manuscript. This previous study is cited in the methods section (because some methods and the CSF dataset were retrieved from the previous study); it should also be prominently discussed under the Results and Discussion sections, addressing the apparent contradiction.

5. Given that the authors perform targeting of brain-enriched transcripts, does this mean they assume the miRNAs measured in blood derive from brain expression? This is not discussed, but very relevant for the main purpose of the manuscript, to "specifically identify microRNAs that could inform about the cognitive status and reserve" (line 487).

6. Also, the 2 sentences declaration regarding the maintenance of synapses and neuronal dendrite branching (Fig 4c) is disappointingly brief; others devote entire articles to present such data convincingly, please detail the experimental data and analyses on which this intriguing declaration is based.

7. Likewise, a deeper explanation of the cognitive score calculation in humans is called for. Also, while hippocampal navigation memory tests make sense for analyzing the treated mice, one needs to describe the logics of those tests used for assessing aging-related cognitive decline in humans, especially given the early damage in deep brain nuclei prior to that of the hippocampus.

8. The aging-related changes in the composition of blood cell miRs may well reflect modified composition of nucleated blood cell types or the general level of inflammation. Therefore, differential blood cell composition data and inflammation biomarkers (such as CRP) should be measured and discussed.

9. Please provide separate analyses of male and female humans and mice, seeking the sex-specific differences in the analyzed criteria. Also, while the reproducible changes in experimental mice may reflect their genomic homogeneity, specific pathogen free (SPF) advantages and identical nutrition, none of those elements exist in humans, which makes the similar findings in mice and men even more surprising.

10. That inflammation, blood brain barrier disruption and neuronal plasticity are all causally involved in aging-related cognitive decline makes sense; however, blood brain barrier disruption is generally considered to be irreversible, raising the question how antisense suppression of three blood-expressed miRs reverses this process and renews neuronal plasticity. This issue as well requires further explanation.

11. The identified three miRs target well-known pathways which are also targeted by many other

miRs. This raises the possibility that these, and no other miRs may have emerged in this study based on their genomic origin and mode of regulation, which should be explained.

12. The small fraction of MCI individuals who shifted into Alzheimer's disease in the studied cohort (line 362) differs from many other reports, where about 80% of such shifts were reported. This raises a question regarding the studied cohort, which may be exceptionally resilient to aging-related memory decline.

13. microRNA naming conventions should be adhered to throughout the manuscript (e.g. figure 3 legend features three different incorrect spellings: micro-146a-5p, micro-RNA--146a-5p, micro-RNA-146a-5p; correct is "mmu-miR-146a-5p"). Correct nomenclature is used only once in the entire manuscript (i.e., "miR-181a-5p").

Methods-related comments

14. Quality control should be more prominently displayed to avoid casting doubt on the rigorous execution of the research performed. Methods should be reported completely, with attention to critical detail. Quality measures undertaken during the analyses should be openly presented and not deleted from code when publishing or sharing with reviewers. Rationale should be provided for the selected statistical methods to avoid the impression of ineptitude.

15. Line 715: Neither main text, nor methods, nor figure legend, nor the discussion specify which blood compartment was utilized for sequencing (referring to "circulating miRNAs" or simply "blood"). Judging by the Methods section, this could have been any of whole blood, PBMCs, plasma, or exosomes. However, these different compartments each possesses vastly different miRNA expression patterns, which is paramount for the interpretation of results. Specifically, miR populations present in different cell pools, exosomes and plasma are shown by others to be very different; therefore, re-check and correction of this paragraph is required.

16. Line 738: 100 ng is the lowest input limit for the TruSeq kit, and additionally, small RNA quality testing (e.g. by Bioanalyzer) is not reported. This should be amended to support downstream analyses.

17. Lines 747, 800, 805: please add the actual quality values to the reported quality control by Bioanalyzer (mRNA) and FastQC.

18. One is curious- why were individuals with unknown nationality (line 779) excluded from this study?!

19. Line 821: sex, not gender is compared here...Line 882: capital C, commemorating Celsius.

20. Line 972: microarray datasets cannot be co-analyzed with RNA-seq data, since they are based on totally different technologies: microarrays 'see' only those miRs for which probes were printed, whereas RNA-seq detects every expressed RNA. This result must be deleted.

21. Code shared for review and reproduction should adhere to best practices in biomedical programming, including sensible file names, functional organization, and commenting (as an unrelated example for good practice, compare here).

22. The currently presented analysis code is a collection of tutorial materials from the respective

packages pasted together; however, one wonders why the structure-giving comments of the tutorial blueprints have been deleted by the authors. Similarly, some of the quality control steps, such as the visual controlling of WGCNA analyses, show problems with quality and were deleted from the script. Please revise and improve.

23. The authors use "auxiliary functions", which are not functions but rather simply lines of code in other, nondescript ("auxFuncxx.R") files, which further complicates retracing of the analyses. It is recommended to source actual, parameterized R functions in these instances, which can be informatively read in the context of code, to avoid complicated code review.

24. While the explanation of requested code in the form of a 87-page PDF file may have been well-intentioned, it requires manual copying of code into the R environment by the reviewer, and includes pages' worth of unnecessary and uninformative R output.

Code-based re-analysis

25. Figure 1: The total output of only four modules is very uncommon for a WGCNA dendrogram (below); particularly in miRNA expression, which is often complex. WGCNA parameters can be iterated for robustness in this case, to exclude chance results (as in this example). The merge step performed in the code is not necessary.

26. Re-alignment of the supplied raw fastq files, variance stabilization, and WGCNA analysis found 11 co-expression modules; however, none of those correlated with cognitive status or sex. This discrepancy should be re-assessed. Further, the code used for alignment and preprocessing (which was not available for review) as well as this result should be reconsidered critically, with particular attention to quality control steps.

27. The (original) correlations of eigengenes to cognition are very shallow (Pearson correlations between 18 and 25%, see below). Figure 1C shows only p-value asterisks, although there would be ample space for displaying numeric values. This mode of presentation limits one's confidence in the claimed large predictive value of circulating miRs on cognition in healthy, young individuals.
28. That all WGCNA modules identified by the authors are very similar in their function is very surprising. Module sizes are not given in the manuscript, but upon re-running the code, the following module sizes emerge: blue 115 miRs, brown 94, turquoise 217, yellow 32. According to this analysis, as much as 93% of blood cells miRNAs are all supposedly involved with cognitive processes and only about 7% of miRNAs do not correlate with cognitive performance (which is rather unlikely).

29. The prominent identification of age-related processes in all modules is likely a result of confounding of the gene ontology analyses, possibly by filtering for only brain-expressed genes. "Confirmed mRNA targets" likely means that the authors used experimentally validated interactions from miRTarBase in these analyses. This could likewise lead to confounding, as validated miRNA interactions are extremely biased and reflect only a very small subset of all true interactions. On the other hand, predicted interactions are notoriously incorrect and yield a large number of false positives. A state-of-the-art publication on miRNA interactions should address these shortcomings in a quantitative manner, as has recently been demonstrated multiple times. The process of miRNA targeting analyses, gene filtering, and GO analysis was not reported in detail (e.g., what was the source of "brain-expressed" genes) and is not available in the supplied code. These analyses should be critically re-examined and described in sufficient detail for enabling in-depth review and

replication.

30. Figure 2, differential expression: "Unwanted variation" is removed via RUVSeq; however, the negative control genes used for this algorithm are all genes in the expression matrix. The reason for this is unclear, no rationale is given. Additionally, the expression matrix is heavily filtered (more than 100 counts in at least half the samples), which is not recommended by the authors of DESeq2, and greatly decimates the already small pool of initial miRs. The MA plot of the differential expression is highly irregular, with almost every studied miR differentially expressed (below).

31. Simple quality control such as mean-SD plots for the count data should be performed routinely in the course of analyses. The mean-SD plots for the filtered counts used in the authors' analysis (left) and the variance-stabilized, unfiltered count data (middle) differ significantly from regular examples of mean-SD plots from the DESeq2 vignette (right). In the case of the authors' original analysis, the prior assumptions of DESeq2 probably do not hold.

32. Downstream analyses based on the same data, namely the feature selection (2F) and eigenexpression in the time course (3A), may also be deeply affected by these irregularities. These analyses should be repeated adhering to the basic principles of biostatistics.

33. The authors preselected three out of 145 miRNAs (the reason for choosing these 145 is not given) out of the human data, calculated eigenexpression values, performed statistical testing (Figure 3B) and compared the eigenexpression values using the standard Mann-Whitney test used in stat compare means(). However, this is a simple nonparametric test without multiple correction, although correction for multiple testing is an absolute must in this case. The potential number of three-miRNA combinations in only those 145 miRNAs used is . Arguably, all miRNAs should be used, not only those 145, but that yields an almost infinite number of combinations. Randomly selecting 12 three-miR-combinations (by setting set.seed() to 1-12) and repeating the authors' analyses for each combination resulted in 7 combinations with significant predictive capacities, five of those with better separation power than the three-miR-signature reported by the authors (below). This is very obviously a statistical artifact caused by the lack of correction for multiple testing. All similar analyses should hence be repeated with adherence to basic statistical principles.

34. Not excluding the excluded samples (due to unique center or missing nationality, see also point 17) also leads to a non-significant difference (p = 0.05) in this comparison. The excluded samples are either MCI with very low values, or controls with high values (below). It is not possible to control exclusion criteria, because nationality and center are not given in the metadata.

35. Note: Due to numerous problems in these first three figures, further examination of code was not performed.

Referee #3 (Remarks for Author):

In the submitted work by Md Rezaul Islam et al., where Schulze, Falkai, Sananbenesi and Fischer are the corresponding authors, they describe a microRNA-signature that correlates with cognition and is a potential target against cognitive decline. Using human data multi-filtration approaches and

animal models. Authors find miRNAs in blood that may serve to classify / predict cognitive status. Indeed, identifying individuals at risk for cognitive decline, before full-blown dementia manifests is an extremely important biomedical goal. They had identified three miRNAs (mir-181,mir-146,mir-148) that correlate with differences in cognitive status in healthy young individuals. There is an attempt to mechanistically link the change in the expression of these miRNAs and brain (dys)function via studies in mice.

Advantages:
The integration of functional datasets for in-depth analysis of the selected miRNAs is valuable.
The analysis of MCI turning to AD is very interesting.
The approach of looking at healthy individuals and generating a miRNA signature using eigenvalue decomposition (SVD) is innovative.
A biomarker of cognitive decline is a great achievement.
Not only was it shown that some miRNAs are correlated with cognitive decline, but also that the miRNAs can be therapeutic targets.
The paper's hypotheses, methods and figures provide in-depth analysis of existing literature, thus can have an interest for the non-specialists.
Authors have properly exhausted existing datasets and gained rich data (human blood cohorts, GWAS data, relevant cell culture and mouse experiments).

Major concerns:

Cohorts:
Subjects have a different cognitive abilities - authors should address the concern that variables such as IQ, education and demographics can act as a confounder to cognitive status at baseline. Are individuals from different groups (control, MCI, and MCI converted to AD) comparable in terms of IQ, demographics, education etc?

Computation and machine learning:
Authors machine-learning approaches suffer from limitations that should be corrected:
Performance matrices should be reported on each occasion machine learning is utilized, for expert reader in the field to be able to assess the method and to draw meaningful conclusions from the analysis.
Authors should report the accuracy of the ML regression algorithms
Authors should explain the validity of the selected features, which is currently limited.
The exact magnitude of the main correlations should be reported.
Authors should characterize the predictive power and quantitative parameters of miRNA signature including specificity and sensitivity, concentration cutoff.

Regress miRNAs on the cognitive decline slop in healthy subjects, if you can gain such data.

Authors acknowledge in discussion that they should have better performed longitudinal study on humans and not only on mice, but they should hopefully find a way to approximate this gap.

Mouse models:
Harmonization required, to be gained by testing similar endpoints (memory performance, neuronal function) with miRNA mimics and miRNA inhibitors.

Authors had tested the simultaneous introduction of miRNA mimics or miRNA inhibitors for all

miRNAs. However, miR-146 and miR-181 negatively correlates with cognition (Figure 2G). Authors are asked to explain or correct the experimental design: should cognition impairing perturbation be to combine overexpression mimics of miR-146 and miR-181 with a miR-148 inhibitor? Should cognition enhancement be driven by miR-146/181 inhibitors together with miR-148 overexpression?

Brain regions studied:
The manuscript focuses on the hippocampus and reports only one cognitive test in mice, which reflects mostly hippocampal function. This should be justified and better if analysis would have been expanded. In this context Wolf et al. may be referenced (Hippocampal volume discriminates between normal cognition; questionable and mild dementia in the elderly. Neurobiol. Aging 22, 177-186. 10.1016/s0197-4580(00)00238-4) and also Dicks et al., (NeuroImage: Clinical 22, 2019, 101786 10.1016/j.nicl.2019.1017860) who found a widespread atrophy pattern with the strongest associations for decline over time for the bilateral hippocampi, insulae and Rolandic opercula (NeuroImage: Clinical 22, 2019, 101786 10.1016/j.nicl.2019.1017860). In contrast, a recent study suggests the precuneus and inferior temporal regions as key regions in physiological and pathological brain aging (Lee et al., Front. Aging Neurosci. 2019 10.3389/fnagi.2019.00147), to make a point that the regions studied in vivo and in vitro should be thoroughly justified. Are the findings relevant to hippocampus-dependent function and not to cognitive function in general?

It should be discussed that extending the study to other regions of the aging brain may be important in the future.

Specific comments on figures:

1. Figure 1: what is the cognitive function difference between males & females? What is the correlation between age and miRNA levels and/or cognitive performance? Denote the value of correlation in Figure 1C.
2. Figure 2H, 3H: Venn diagram of feature overlap between the targets of the three miRNAs and the genes found in GWAS studies, or the up-regulated/down-regulated genes in AD datasets.
3. Fig. 3H: Venn diagram of feature overlap needed.
Fig 3: How come that Discussion and interpretation:
Authors do not rule out the simplest working hypothesis that the miRNAs simply correlate with aging and are not mechanistically linked to a brain disease. miRNA markers of aging might be important to report and this should be clarified in discussion.
4. enrichment values of 0.92, 1.02 and 1.15 are significant (human AD transcripts)?
5. Expanded view Fig. 2C: what is the difference between time points 1 and 2?
6. Expanded view Fig. 2C: What does each small square represent?
7. Expanded view Fig. 2C: Why are there two exosome groups, one with N=6 and one with N=16?
8. Expanded view Fig. 2C: the title "exosome" is written upside down at the bottom of the figure.
9. Expanded view Fig. 2E: what does each pixel stand for?
10. Expanded view Fig. 2E: How was the overlap between cohort 1 and cohort 2 measured? By correlation? If so, what did you correlate, considering the fact that these were two different cohorts?
11. Expanded view Fig. 2E: what was the miRNA expression?
12. Expanded view Fig. 2F: does each dot represent one miRNA?
13. Expanded view Fig. 5C: is the overlap between "home cage group" and "learning group" statistically significant?
14. Expanded view Fig. 5C: What are the axes units?

15. Expanded view Fig. 6: is a higher level of proximity indicative of greater or smaller distance from the former platform?
16. Expanded view Fig. 6: Why does the figure reflect impaired memory retrieval?
17. Expanded view Fig. 7: not informative: the 3-miRNA signature is shown in the next figure (expanded view figure 8) and not in figure 7.
18. Expanded view Fig. 7: are there two "CA1" labels in panel A?
19. Expanded view Fig. 9: it is surprising that the time-dependent memory loss is not correlated with temporal increase in the 3-miR expression.
20. Expanded view Fig. 9: Is the 3-miR signature in "advanced age group" significantly different from that in the "middle-age group"?
21. Expanded view Fig. 14D: discrepancy between the text description (MElightgreen module) and figure itself (MEblue module).

Minor textual comments:

1. General comment: the authors keep using the word "substantialize" throughout the text instead of "substantiate" which is more appropriate. This should be corrected.
2. p. 8, line 269: the word "that" appears twice.
3. p. 17 line 546: "complimentary" should be replaced by "complementary" (complimentary means "free of charge")
4. p. 17 line 561: should be "minimally invasive" instead of "minimal invasive".
5. p. 17 line 563: please rewrite "...further supports their potential as suitable biomarkers" instead of the sentence as it is written now.
6. p. 17 line 568: "comparable" and not "comparably".
7. p. 33, line 1081: please rephrase to "in the hippocampus of cognitively intact 3 month-old and cognitively impaired 16.5 month-old mice"
8. p. 33, line 1091: rephrase to "...the genes that were down-regulated".
9. p. 33, line 1110: rephrase to "Proteins that are over- and under-expressed..."
10. p. 37, line 1246: remove the word "was".
11. p. 40, lines 1339-1341: please add bold "A" where needed, to denote referring to panel A in the text.
12. p. 42, line 1386: typo in "3-mciroRNA" (should be "3-microRNA").
13. p. 42, line 1405: change "suggestion" to "suggesting".

Reviewer 1

Major concerns:

#1.

In the functional experiments provided in Fig. 4, 6 and 7, the authors employ synthetic microRNA mimics or anti-miRs to manipulate microRNA candidates in primary neurons and in the mouse brain in vivo. It is imperative to demonstrate that the treatments actually lead to the expected effects, e.g. microRNA overexpression or inhibition in the respective systems. This could for example be achieved by so-called microRNA sensor assays which monitor microRNA activity via the expression of fluorescent reporter genes coupled to the cognate microRNA binding sites. Alternatively, microRNA levels (if affected) could be measured by qPCR or FISH.

Using qPCR analyses we now show within novel Expanded View Figure 9A that increased miR levels are detected when the miR-mimics are administered to the cell cultures. We also show that the corresponding miR levels are decreased when the anti-miR-mix is administered. These data are shown in novel Expanded View Figure 14 A-B. Moreover, we generated corresponding *in vivo* data and analyzed miR expression via qPCR, when the anti-miR-mix was injected into the hippocampus of mice. Please see novel Expanded View Figure 14 C-D and Expanded View Figure 16. All of these data are described in the corresponding figure legends and within lines 345-346, 463-466, 483-486, and 509-511 of the revised manuscript.

#2.

-With regard to the in vivo injections (Fig. 6), it would in addition be important to monitor the spreading and stability of the anti-miRs in the tissue, e.g. by using fluorescently labeled oligonucleotides.

To address this question, we have now employed qPCR analysis to assay the expression of the 3 miRs in the hippocampal cornu ammonis (CA) region representing the hippocampus without the dentate gyrus (DG) and DG. Since the injections were targeted to the CA region, we reasoned that the specific analysis of CA and DG would allow us to estimate potential spreading. While miR levels were decreased in the CA region upon injection of the anti-miR-mix, levels were not affected in the DG. The results are summarized in novel Expanded View Figure 16 and are described within lines 483-486, and 509-511 of the revised manuscript.

#3.

-Behavioral experiments presented in Fig. 6 are largely underpowered, with n<10 animals for most experimental conditions. It would therefore be important to replicate these findings with an independent, similarly sized cohort of animals. In addition, a statistical assessment of the behavioural data taking into consideration the sex of the mice has to be provided.

We have now repeated the behavioral experiment for the aging cohort described in Fig 6 A-F in a larger cohort of mice (young-control, n = 18, old-control, n = 18, old miR-inhibitor mix, n = 20).

We could confirm our initial results and present the novel data now in the revised Fig 6 panel B-F of our manuscript and report the new numbers in the figure legend. We also mention that in this experiment all mice were male. The reason is that we are not allowed to age mice in our animal facility but can order aged male mice from a commercial supplier (Janvier) as described in the material and methods part (see line 689).

As suggested by this reviewer we now also present additional data from the experiment employing APP mice (Figure 6 H-L). In contrast to the experiment in aged mice described above, it is more difficult to address this question, since we were unable to order 7 month-old APP mice and could also not obtain a sufficient number of mice from collaborators. In the previous version of the manuscript, we had only presented data from male APP mice, since we also only used male mice in the aging experiment. In the original experiment we had however analyzed male and female mice from our colony. We now present the combined data and furthermore show that the combined data from the both genders yielded similar results as previously observed, namely that injection of the anti-miR-mix ameliorates learning defects in APP mice. This data is now presented as revised panels H-I within Fig.6 and described in the corresponding figure legend. (WT-control, n = 17, male: 9, female: 8; APP-control, n = 8, male: 6, female: 2; APP anti-miR-mix, n = 12, male: 8, female: 4). Please see legend of Fig. 6.

#4.

- The design of the anti-miR experiments is missing an important control (anti-miR treatment in wt mice), which is not considered good scientific practice. These experiments should follow a classical 2x2 design (2 genotypes, 2 treatments). Data should be assessed by a 2-way ANOVA following appropriate post-hoc tests. Data variance should be reported by standard deviation instead of standard error of the mean.

We like to state that we analyzed the presented data using a Mixed-effects analysis followed by a post-hoc test to assess the difference in escape latency across training trials. We now specifically refer to this in the legend of the revised Fig. 6 and also refer to the standard deviation. Regarding the experimental design, our aim was not to study the effect of the anti-miR-mix in wild type mice and thus we used the experimental design illustrated in Fig. 6. Although, it might be helpful to use the suggested 2x2 design, the choice for an animal experiment is a complex decision that is also based on the consideration of animal welfare, practicality and scientific rationale. Researchers are requested to employ every possibility to reduce any unnecessary use of animals and minimize the number of animals included in experimental approaches. The principles of the three R's (Reduction, Refinement, and Replacement) have to be applied and the local animal care committees in Germany became much more restrictive in the recent years. Thus, a 2x2 design is not considered relevant for the scientific question we are addressing, namely the question if the anti-miR-mix would affect disease phenotypes. In addition, our approach is not uncommon and documented in the literature (e.g. see PMID: 28540926, PMID: 32342860).

However, we had conducted a pilot experiment that can address this reviewers question if the anti-miR-mix treatment would affect learning behavior in healthy wild type mice, without the need to perform a 2x2 design for all experiments. To initially address the question if administration of the anti-miR-mix would have detrimental effects in healthy animals, 3-month-old male mice had been treated with either the anti-miR mix or scramble control RNA and then subjected to behavior testing. Our data suggest that in this experimental setting the anti-miR-mix does not affect learning behavior. The new results are summarized in novel Expanded View Figure 15 and described within line 474-476 of the revised manuscript.

More minor concerns:

#1.

-miR-181 has been previously shown to negatively regulate dendritic spine development (Saba et al., 2012). Therefore, it would be very informative to disentangle the contribution of the individual microRNA mimics to the observed reductions in spine density and length in

Fig. 4. This could be addressed by transfecting individual miRNA mimics followed by spine analysis and electrophysiological recordings.

We performed a new set of experiments to address this question and present the results within novel Expanded View Fig 9B-C. In brief, individual overexpression of each microRNA reduces spine density and neuronal activity. Please see also lines 351-352 of the revised manuscript.

#2.

-Little is known regarding the cell-type specific expression of the microRNA candidates. There is some data suggesting a preferential expression of miR-146 in microglia, which complicates the interpretation of the functional data in neurons. Therefore, the authors should perform FISH analysis to assess the cellular localization of the studied microRNA candidates in their mixed primary neuronal cultures and hippocampal slices.

We had addressed this issue – in part – via the data shown in Fig 3D of the former manuscript. In addition, we now prepared RNA from mouse neuron-enriched primary hippocampal cultures, primary astrocytes and primary microglia and tested the miR-expression via qPCR. miR-146a-5p is significantly enriched in microglia but lower levels can also be detected in neurons. miR-148a-3p and miR-181a-5p were enriched in neurons. We present these data as novel panel D within Fig 3. Please also see lines 294-302, 1076-1089 of the revised manuscript.

#3.

-In Fig. 7, the authors who a rescue of the expression of specific synaptic genes in old or APP-transgenic mice by the injection of the anti-miR mix. Are these genes direct targets of the candidates miRNAs? If so, can the authors provide some target validation experiments, e.g. using luciferase reporter gene assays.

As suggested, we have performed luciferase assay for selected genes that were found to be similarly regulated in aged and APP mice. We could confirm that the 3 candidate microRNAs regulate these genes. These data are presented as novel Expanded View Fig 18 and are described within 551-552.

Reviewer 1 continues *"Furthermore, do the 3'UTRs of genes upregulated by the anti-miR mix injection display an enrichment of the respective microRNA binding sites compared to the sequencing background? Such an analysis could also be used as an alternative validation of the effectiveness of the anti-miR injections."*

We retrieved the genes with 3' UTR binding sequences for miR-146a-5p, miR-148a-3p and miR-181a-5p from miRWalk database. We provide this list within a new supplementary table (Appendix table 16). Next, genes having predicted binding sites for these three microRNAs were overlapped to the blue and lightgreen modules from aged and APP/PS1 mice, respectively. We observed an above chance 35% overlap (Fisher's Exact test,****$P < 0.0001$) for the blue module detected in aged mice and a 38% non-random overlap for the lightgreen module found in APP mice (Fisher's Exact test, ****$P < 0.0001$). Of note, both of these modules display increased gene expression after administration of the anti-miR mix (Figure 7). See novel Appendix table 16 and lines 548-551 of the revised manuscript.

Reviewer 2

Conceptual queries

#1

The implicated pan-species impact of the identified miRs is highly surprising, especially considering that one third of the human-expressed miRs are primate- specific, and could not be detected in mice. The presented findings may therefore be interpreted as implying that aging-dependent cognitive decline is common to all mammals and that it is evolutionarily conserved, which is utterly surprising. This issue needs to be referred to.

Based on our data we suggested that the role of the 3 miRs identified in our study might be conserved amongst mice and humans. This view is supported by the fact that the 3 miRs are 100% conserved amongst mice and humans. To illustrate this, we retrieved the mature sequences of these three microRNAs from miRBase (v 22.1) and performed a multiple sequence alignment using Clustal omega (https://www.ebi.ac.uk/Tools/msa/clustalo/).

```
hsa-miR-146a-5p     UGAGAACUGAAUUCCAUGGGUU     22
mmu-miR-146a-5p     UGAGAACUGAAUUCCAUGGGUU     22
                    **********************

hsa-miR-148a-3p     UCAGUGCACUACAGAACUUUGU     22
mmu-miR-148a-3p     UCAGUGCACUACAGAACUUUGU     22
                    **********************

hsa-miR-181a-5p     AACAUUCAACGCUGUCGGUGAGU    23
mmu-miR-181a-5p     AACAUUCAACGCUGUCGGUGAGU    23
                    ***********************
```

Our approach is based on the combined analysis of human data and mouse models in a feed-forward feed-backward approach. Thus, it is expected - and not surprising - to identify candidate microRNAs with conserved function.

Regarding the second part of the questions: We did not state in the previous version of our manuscript that "*cognitive decline is common to all mammals and that it is evolutionary conserved*". Nevertheless, the literature suggests that common mechanisms can be identified (e.g. see PMID 32877673 or 26607684). We now refer to these issues within lines 578-580 and 658-660 of the revised manuscript.

#2

Likewise, it is surprising to learn that the authors kept seeking data which ***support*** *their working hypothesis (line 308): in exploratory research, one should try to CHALLENGE one's theory, rather than making efforts to support it.*

We believe that this is a misunderstanding. First, we do not find any statement to suggest that we had only designed experiments that would support our hypothesis, while ignoring other approaches. Each experiment has been designed to prove or disprove a hypothesis. We carefully checked the original manuscript and found the wording "*In sum, these data support the hypothesis…*" and "*These data further support the hypothesis…*" . However, here we simply summarize the results of several experiments that had been described in the text before and conclude that the data support our initial hypothesis. This is something fundamentally different from the issue raised by this reviewer.

#3

The abstract correctly highlights the need for simple and low-cost therapeutics; but repeated preventive treatment by intrathecal injection of three oligonucleotide mixtures into the brain of apparently healthy individuals is neither simple nor cheap. Please comment on the approach and the timeline predicted to last between treatments.

We agree with the reviewer that simple and low-cost therapeutics would be desirable. However, in the abstract and throughout the manuscript we never refer to *low-cost therapeutics* but the need for "*inexpensive and minimal invasive*" biomarker that could be used in screening approaches with the aim to identify risk individuals that could then undergo further diagnostics and eventually stratified therapies. We also referred to potential approaches how the analysis of microRNAs might be simplified in the future (now lines 690-688 of the revised manuscript).

Related to therapeutics, we suggest that RNA therapeutics is a promising research field also for CNS diseases and we referred to this in our previous version of the manuscript. We extended this part now and specifically refer to clinical approaches in which RNA drugs are delivered to patients. As this reviewer asked for a "timeline", we like to refer - as an example - to clinical studies related to Huntington's disease. Here, patients were treated every 4 weeks with ASOs via intrathecal administration (e.g. see PMID:31059641). We address this issue now in more detail. Please see lines 665-666 of the revised manuscript.

#4

That the identified 3 miRs signature is derived from nucleated blood cells raises the question of why their cumulative antisense suppression in the brain is effective; an in-depth explanation of the identification of this signature and the predicted cellular and molecular route involved and the shared impact on blood and brain cells may help to convince the readers, especially if accompanied by a comparison to the other smRNA signature proposed by these authors in their 2019 article, where they identified a three-miRNA-three-piRNA-signature for AD, which includes miR-27a-3p, miR-30a-5p, and miR-34c; three entirely different miRNAs than the ones described in their current manuscript. This previous study is cited in the methods section (because some methods and the CSF dataset were retrieved from the previous study); it should also be prominently discussed under the Results and Discussion sections, addressing the apparent contradiction.

This reviewer formulates multiple thoughts, which we will address one by one.

To answer this reviewer's question "*why their cumulative antisense suppression in the brain is effective",* we like to reiterate that we observed increased levels of the 3 miR signature not only in blood but also in the CSF of MCI patients and in brain tissue obtained from the employed animal models. On this basis we conducted the pre-clinical experiments to test the potential therapeutic efficacy of targeting the 3 miR signature in aged and APP mice. At present, we do not suggest that targeting the 3 miR signature in the blood would be a suitable therapeutic strategy.

The additional request for "*in-depth explanation of the identification of this signature and the predicted cellular and molecular route involved and the shared impact on blood and brain cells"* is an important question but has to be addressed independent from the question why administering the anti-miR-mix to the brain shows therapeutic efficacy. In the revised version of the manuscript, we provide a more detailed discussion on this issue. Please see lines 648-658 of the revised manuscript.

Finally, this reviewer refers to our study by Jain et al., which is based on the analysis of CSF. Moreover, in this study we used a classical approach and compared controls (patients that underwent CSF collection for reasons not related to neurodegenerative diseases) to patients that were already diagnosed with MCI or AD. In our current manuscript we now challenge such type of approaches when the aim is the identification of biomarker for early detection of patients at risk to develop pathological cognitive decline. Thus, we suggest and follow an alternative strategy that was outlined in Expanded View Figure 1. We address this issue now specifically within lines 568-573.

#5

Given that the authors perform targeting of ***brain****-enriched transcripts, does this mean they assume the miRNAs measured in blood derive from brain expression? This is not discussed, but very relevant for the main purpose of the manuscript, to "specifically identify microRNAs that could inform about the cognitive status and reserve" (line 487).*

We understand that this reviewer refers to the following sentence that we wrote in the discussion of the former manuscript: "*Rather, we employed an integrative approach starting with the analysis of cognitive variability in healthy humans and subsequently used multiple filtering steps with the aim to specifically identify microRNAs that could inform about the cognitive status and reserve and help to detect individuals at risk for pathological memory impairment."*

We believe that part of his/her questions refers to the data shown in Fig 2I of the previous version of our manuscript. Here we performed a GO-analysis for microRNA targets that were filtered based on their expression in the brain. First, we like to state that this type of analysis should of course be viewed as an exploratory approach. This is also why we subsequently followed up with mechanistic experiments described in Figs. 3 and 4.

To address this reviewer's concern, we have now re-analyzed the data and excluded the specific analysis of "*brain-enriched targets*" and rather present the analysis without prior filtering steps. Please note, that the main message that we had communicated remains the same. See novel panel I of Fig2, lines 262-273 of the revised manuscript and the revised section "Gene ontology and pathway analysis of microRNA target gene" within the Materials and methods section.

In addition, we discuss now in greater detail how expression changes in blood and brain might be linked. Please see lines 648-658 of the revised manuscript.

#6

Also, the 2 sentences declaration regarding the maintenance of synapses and neuronal dendrite branching (Fig 4c) is disappointingly brief; others devote entire articles to present such data convincingly, please detail the experimental data and analyses on which this intriguing declaration is based.

With all respect, we disagree with this comment. We do not know to which 2 sentences he/she is specifically referring to and which part of the data is not presented "convincingly". We describe in sufficient detail our corresponding experimental approaches. Moreover, we did not study dendritic branching. As part of our experimental approach, we analysis synapse and dendrite number and performed MEA measurements. The aim of these experiments was to further test the hypothesis that the candidate microRNAs may or may not play a role in synaptic function and not to elucidate all synaptic parameters that might be affected by the 3 microRNAs described in this study.

In response to minor comment # 1 made by reviewer 1, we have now also studied the effect of the individual overexpression of each microRNAs on spine density and neuronal activity. Please see our response to minor comment #1, reviewer 1, novel Expanded View Figure 9 B-C and also lines 351-352 of the revised manuscript.

#7

Likewise, a deeper explanation of the cognitive score calculation in humans is called for. Also, while hippocampal navigation memory tests make sense for analyzing the treated mice, one needs to describe the logics of those tests used for assessing aging-related cognitive decline in humans, especially given the early damage in deep brain nuclei prior to that of the hippocampus.

(1) We have now expanded our explanation of the cognitive score calculation within the corresponding material and methods section. See lines 721-735.
(2) We understand that the second part of the question also generally refers to the debate if animal models are suitable to understand human cognitive diseases. Of course, no animal model can fully recapitulate a human disease. Nevertheless, we believe it is fair to use the Morris water maze test in the context of our project since the hippocampus is one of the regions affected early in humans that develop cognitive decline and eventually AD. Moreover, hippocampus-dependent function can be routinely assayed in mice via the water maze test and is impaired during aging and in neurodegenerative diseases (e.g. see PMID 32298784; 20448184, 21421011, 31422072) and allows the longitudinal analysis of learning in mice. We described these issues in the previous version of our manuscript and have now expanded our explanation and added additional citations. See lines 190-194 of the revised manuscript.

#8

The aging-related changes in the composition of blood cell miRs may well reflect modified composition of nucleated blood cell types or the general level of inflammation. Therefore, differential blood cell composition data and inflammation biomarkers (such as CRP) should be measured and discussed.

This question is related to his /her comment #5. We outlined our reasoning for analyzing blood samples collected via Pax-gene tubes. At the same time, we acknowledge, that it would be interesting to address the question if changes in the expression of the 3 microRNAs in circulation are for example due to the presence of brain-derived extracellular vesicles, cell-free microRNAs that originate from the brain other organs or alterations in the composition of bloods cells. We discuss this issue now within lines 648-658 of the revised manuscript but we would argue that it is beyond the scope of our manuscript (that by now already includes 7 main and 18 expanded view figures) to conclusively address these questions experimentally.

#9

Please provide separate analyses of male and female humans and mice, seeking the sex-specific differences in the analyzed criteria. Also, while the reproducible changes in experimental mice may reflect their genomic homogeneity, specific pathogen free (SPF) advantages and identical nutrition, none of those elements exist in humans, which makes the similar findings in mice and men even more surprising.

The second part of this this comment is essentially the same as comment #1 by this reviewer, stating that it would be surprising to find the same 3 microRNAs implicated with age-associated memory decline in mice and humans. Please refer to our response to comment 1. In addition to our response to comment 1, we like to mention that there are numerous examples of molecules and molecular processes that appear to serve similar functions in mice and humans, also related to cognitive diseases. For example, general processes such as neuroinflammation or synaptic loss have been linked to cognitive decline in humans (e.g. PMID: 30930767) and rodents (e.g. PMID: 19047808). In turn, aerobic exercise is an environmental stimulus that can improve cognitive performance in mice and humans (e.g. PMID: 10557337; 17468743; 34067861; 33820516) making animals a suitable model to elucidate the underlying mechanisms. Related to very specific molecules we like to refer as an example to the genes that encode the six mammalian histone 3 lysine 4 (H3K4) methyltransferases that are all genetically linked to cognitive diseases in humans (PMID:29309830; 24434855). Similarly, the corresponding animal models analyzed so far exhibit cognitive dysfunction (PMID:31606247; 23426673; 30891914; 28723559).

Regarding the question related to sex effects, we now specifically provide this information for the animal experiments. Please see figure legends of Figs 2 and 6.

To study sex-specific differences in the human datasets shown in Figs. 1, 5 and Expanded View Fig 10 is an interesting question that we decided not to address in our study. We have realized that in the original manuscript this was not clearly stated in the results section. We have made it now clear in the revised manuscript. Please see line 160 and 1348-1349.

As this reviewer pointed out, there is greater genomic and environmental *homogeneity* in laboratory mice when compared to humans. However, we do not follow this reviewer's argument that this is a problem. Thus, human data is always descriptive, often cross sectional, limited by sample size and not gender balanced. As we outlined in detail, to combine such human data, with longitudinal and mechanistic experiments in mice is therefore - in our view - a promising strategy.

We combined both male and female human data to increase sample size and improve statistical power and precision. However, we like to reiterate that the human data presented in our study should not be judged independent of the other data presented within Figs 2,3,4,6 and 7.

To nevertheless address this reviewer's questions, we have now re-visited our human data(Fig 1C, Fig 5B, E, F and Expanded View Fig. 10) and depict it in the figure below, all along with results from analogous analyses but splitted by sex. Panels on the left show always results from using both sexes together, while the right panel shows the data for each sex separately. In panel (A), results from co-expression analyses are shown, while panels B, C, D and E show the comparisons from our 3 miRNA signature across the different human datasets.

Generally, while splitting the data into male and females did not always yield significant results in both groups, the described changes always go in the same direction

In the case of WGCNA, results are comparable or both males and females. Indeed, the correlation values between each of the modules and the weighted memory score go in the same direction in both sexes, although for males the correlations are lower and the p-values higher because they are the smaller dataset (74 females vs 58 males).

Comparisons of the miRNA signature across different datasets and conditions (B, C, D and E below) show that first, patterns of change are similar for both sexes but that second, significance may be lost on the sex having smaller amount of samples. Regarding the first point, it is encouraging to see that independently of sex, the miRNA signature increases along with cognitive decline. Regarding the second point, it is well known that p-values depend on sample size (Sullivan and Feinn, 2012; Irizarry and Love, 2017) and therefore significances may be lost when splitting the data by sex.

Nevertheless, for the miRNA signature, we have gone beyond p-values and report effect sizes (Cohen's d) along with their corresponding confidence intervals in the caption of the figure below. The advantage of using coefficient 'd' is that it is a measure independent of sample size (Sullivan and Fein, 2012). As it can be read in the caption, the magnitude and sign of the effect size always goes in the same direction for both males and females and amthc the combined data.

To summarize, we agree that analyzing gender-specific differences is a relevant question. However, given the limited amount of human data, an analysis of this kind is not possible and therefore it is required to pool both sexes together. Indeed, in all of our experiments in which statistical significance is compromised when splitting data by sex, it is the smaller dataset that is affected the most. Thus, combining both male and female increases sample size, improves statistical power and precision in our results.

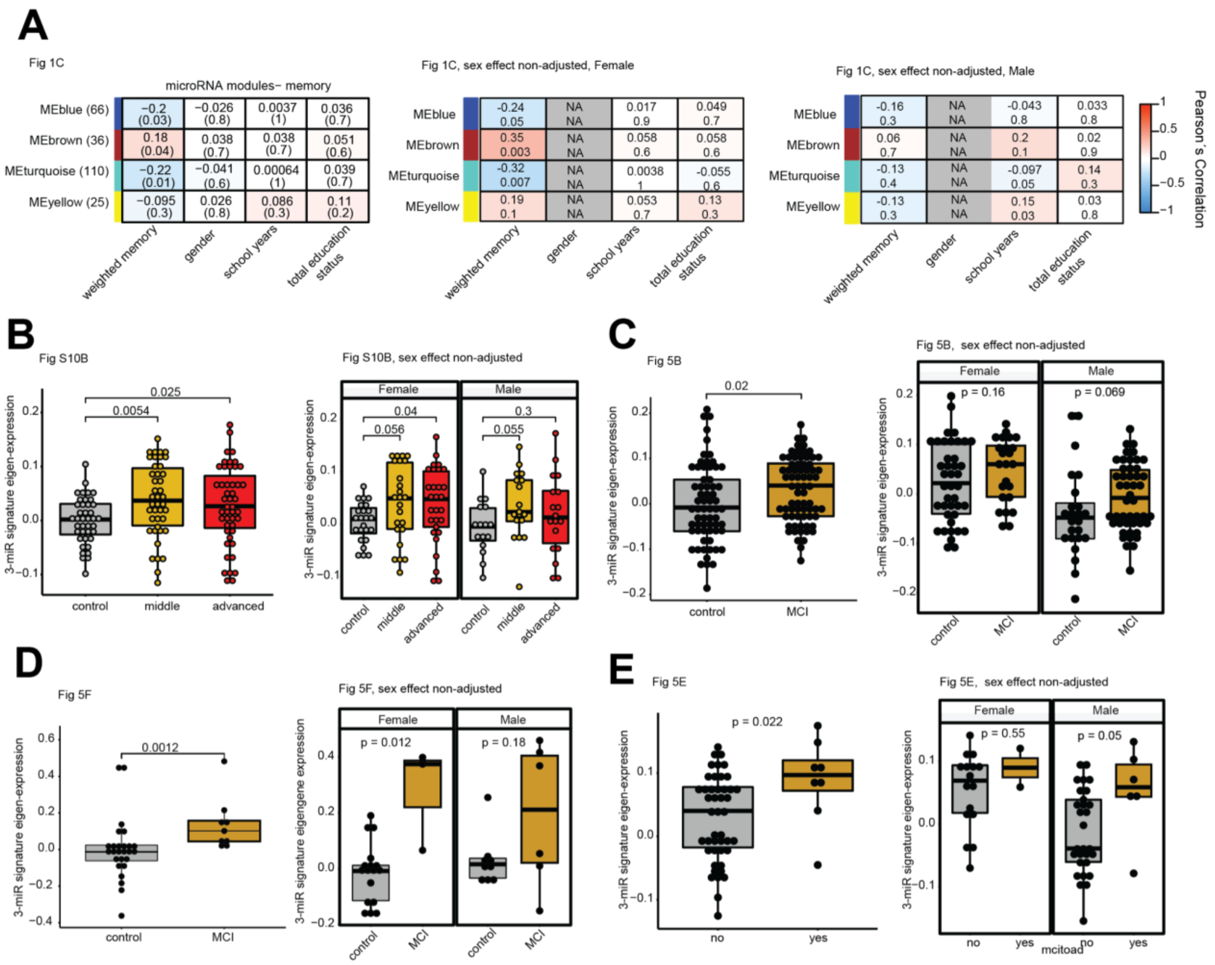

Figure in response to comment 9: Human data split in the sex-specific analysis.

Left panels always show the data as depicted in the corresponding figure panel of the manuscript using pooled data from male and female and corrected for sex. Middle panel always shows the data from females and the right panel always shows the data from males only. ***A.*** *As shown in Fig 1C, a significant correlation of the co-expression modules with cognitive function in healthy humans was observed for the blue, brown and turquoise modules using all data that was corrected for sex (same as n = 132). Performing the same data for females only (n = 74) identifies the brown and turquoise model as significantly correlated, while no module is significant in males. However, the correlation values between blue, brown and turquoise modules and the weighted memory score all go in the same direction in both males and females. Please note that the male dataset is the smallest.* ***B.*** *As shown in expanded view Fig. 10 the eigen-expression of the 3 microRNA signature is significantly increased with age (n = 129; $n_{control}$ = 40; ; n_{middle} = 42; ; $n_{advanced}$ = 47, effect size, middle vs control : medium, 0.65 [0.21, 1.11] (cohen´s d [lower confidence interval, upper confidence interval]);effect size, advanced vs control : small, 0.46 [0.03, 0.9] (cohen´s d [lower confidence interval, upper confidence interval]. When data were analyzed without adjusting sequencing data for sex effect, similar effect size was observed for male (ncontrol = 16; ; nmiddleel = 18; ; nadvanced = 18;; effect size, middle vs control : medium, 0.68 [-0.03, 1.4], advanced vs control: small, 0.35[-0.36, 1.05]) and female (ncontrol = 24; ; nmiddleel = 24; ; nadvanced = 29; effect size, middle vs control: meidum, 0.63 [0.03, 1.22], advanced vs control: medium, 0.55 [-0.01, 1.11]), particularly for middle vs control comparison.*

C. *As shown in Fig. 5 C, the eigen-expression of the 3-microRNA signature is increased in MCI patients when compared to control (effect size: small, 0.38 [0.04, 0.72];MCI patients n=71, controls n=65). When we only analyzed females (effect size: small, 0.34 [-0.17, 0.85], control: females = 44, MCI: females 24; total = 68) we observed a similar effect size. The same was true for males (effect size: small, 0.43 [-0.07, 0.94], control: males = 24; MCI: males = 50, total = 74). However, the p-values for females (P = 0.16) and males (P = 0.069) were close to significance (more so for the males that represent the larger sample).* ***D.*** *As shown in Fig. 5 F, the eigen-expression of the 3 microRNA signature is increased (effect size: large, 1.85 [0.95, 2.76]) in the CSF of MCI patients when compared to control (MCI patients n=9, controls n=26, total = 35,). Significance is maintained when analyzing the female data alone (effect size: large, 2.47 [0.92, 4.02],MCI patients n=3, controls n=17, total = 20,) but not observed when analyzing in lower sample sized male individuals (effect size: large, 0.95 [-0.24, 2.15]. MCI patients n=6, controls n=9, total = 15). The effect size is, however, similar in all 3 analyses.*

E*. As shown in Fig 5E, the eigen-expression of the 3 microRNA signature is increased (effect size: large, 0.89 [0.1,1.68] in MCI patients that converted to AD (n = 7) when all data is analyzed and compared to patients with stable MCI (n = 47). The data is not significant when female (effect size: medium, 0.62 [-0.99, 2.22], Stable MCI n = 16; MCI converted to AD n = 2, total = 18,) and male (effect size: large, 0.96 [0.02, 1.9],Stable MCI n = 29; MCI converted to AD n = 6, total = 35,) individuals are analyzed separately. The male data represents the larger dataset is almost significant (P = 0.05). Most importantly, effect size is similar in all 3 analyses.*

Effect size is presented as Cohen´s d and lower-upper confidence intervals in the parentheses.

To conclude, it is not wrong and neither uncommon to combine sequencing data from males and females (Parikshak et al., 2016) (Swarup et al. 2019). On the contrary, combining both male and female increases sample size, improves statistical power and precision in our results. Moreover, we show now that the described changes go in the same direction in males and females and that the effect size is similar when splitting the data by sex.

References

1. Sullivan GM, Feinn R. Using Effect Size-or Why the P Value Is Not Enough. *J Grad Med Educ*. 2012;4(3):279-282. doi:10.4300/JGME-D-12-00156.1
2. Irizarry R., Love M. Data Analysis for the Life Sciences With R. CRC Press, 2017.
3. Parikshak NN, Swarup V, Belgard TG, Irimia M, Ramaswami G, Gandal MJ, Hartl C, Leppa V, Ubieta LT, Huang J, Lowe JK, Blencowe BJ, Horvath S, Geschwind DH. Genome-wide changes in lncRNA, splicing, and regional gene expression patterns in autism. Nature. 2016 Dec 15;540(7633):423-427.
4. Swarup V, Hinz FI, Rexach JE, Noguchi KI, Toyoshiba H, Oda A, Hirai K, Sarkar A, Seyfried NT, Cheng C, Haggarty SJ; International Frontotemporal Dementia Genomics Consortium, Grossman M, Van Deerlin VM, Trojanowski JQ, Lah JJ, Levey AI, Kondou S, Geschwind DH. Identification of evolutionarily conserved gene networks mediating neurodegenerative dementia. Nat Med. 2019 Jan;25(1):152-164

#10

That inflammation, blood brain barrier disruption and neuronal plasticity are all causally involved in aging-related cognitive decline makes sense; however, blood brain barrier disruption is generally considered to be irreversible, raising the question how antisense suppression of three blood-expressed miRs reverses this process and renews neuronal plasticity. This issue as well requires further explanation.

We now discuss this issue in more detail. Please see lines 666-670 of the revised manuscript.

#11

The identified three miRs target well-known pathways which are also targeted by many other miRs. This raises the possibility that these, and no other miRs may have emerged in this study based on their genomic origin and mode of regulation, which should be explained.

We now discuss this issue in more detail. Please see lines 633-634 of the revised manuscript.

#12

The small fraction of MCI individuals who shifted into Alzheimer's disease in the studied cohort (line 362) differs from many other reports, where about 80% of such shifts were reported. This raises a question regarding the studied cohort, which may be exceptionally resilient to aging-related memory decline.

This reviewer suggests that in other studies 80% of the individuals converted from MCI to AD. However, this is certainly not the case within 2 years following the initial diagnosis as in our study. Rather our data is in agreement with the literature suggesting conversion rates from 10-30% in such a time window (e.g. see PMID 9447429; 19236314; 24174927).

#13

microRNA naming conventions should be adhered to throughout the manuscript (e.g. figure 3 legend features three different incorrect spellings: micro-146a-5p, micro-RNA--146a-5p, micro-RNA-146a-5p; correct is "mmu-miR-146a-5p"). Correct nomenclature is used only once in the entire manuscript (i.e., "miR-181a-5p").

We corrected this. When generally referring to microRNAs we say "microRNA". When referring to specific microRNAs we say "miR-181a-5p", miR-146a-5p" or "miR-148a-3p".

Methods-related comments

#14

Quality control should be more prominently displayed to avoid casting doubt on the rigorous execution of the research performed. Methods should be reported completely, with attention

to critical detail.Quality measures undertaken during the analyses should be openly presented and not deleted from code when publishing or sharing with reviewers.

Since this is a rather general remark, we have now carefully revisited the code. We assume that the reviewer is referring to the whole network connectivity plot using plotDendroAndColors function in previously provided code, since we noticed that this step was missing for some datasets. We have now added this in the revised code and refer to this within line 973-974.

He/She continues: *Rationale should be provided for the selected statistical methods to avoid the impression of ineptitude.*

We understand that this is also a rather general remark. Our team includes statisticians that were involved in all steps of data analysis. Moreover, we are confident that our analytical approach is rigorously designed and that the applied statistical tests are credible. Since this reviewer asks a number of more specific question related to data analysis within his/her comments 25-35, we like to refer to our answers to these questions.

#15

Line 715: Neither main text, nor methods, nor figure legend, nor the discussion specify which blood compartment was utilized for sequencing (referring to "circulating miRNAs" or simply "blood"). Judging by the Methods section, this could have been any of whole blood, PBMCs, plasma, or exosomes. However, these different compartments each possess vastly different miRNA expression patterns, which is paramount for the interpretation of results. Specifically, miR populations present in different cell pools, exosomes and plasma are shown by others to be very different; therefore, re-check and correction of this paragraph is required.

We mentioned in the previous manuscript that we analyzed whole blood collected via Pax-gene tubes (now lines 147-152 and 377-378, Legends of Figs 1A, 5B). To avoid any misunderstandings, we now repeat this fact in the revised manuscript. Please see line 155 of the revised manuscript.

#16

Line 738: 100 ng is the lowest input limit for the TruSeq kit, and additionally, small RNA quality testing (e.g. by Bioanalyzer) is not reported. This should be amended to support downstream analyses.

The RNA amount used for sequencing is within the suggested range of the kit and allowed us to follow the protocol recommended by the provider. While the obtained RNA integrity (RIN) value is a suitable estimate for the integrity of long RNAs such and lncRNA or mRNAs, in our view it is less meaningful for smallRNAs and especially microRNAs that highly stable. Thus, we did not report this information. RIN values are now provided in the appendix tables 1 and 12 of the revised manuscript.

#17

Lines 747, 800, 805: please add the actual quality values to the reported quality control by Bioanalyzer (mRNA) and FastQC.

We understand that here the reviewer is referring to our mRNA sequencing experiments described in Figs 3, 5G and 7. RIN value of the analyzed samples were above 8. Mean quality score of the analyzed samples based on FastQC analyses were above 30. We now mention this within lines 943 and 951 of the revised manuscript.

#18

One is curious- why were individuals with unknown nationality (line 779) excluded from this study?!

This is the same issue as raised in comment 34. Please find further detail on this issue in response to comment 34.

#19

Line 821: sex, not gender is compared here… Line 882: capital C, commemorating Celsius.

We have corrected "sex" for "gender" as suggested. In addition, we now carefully checked all abbreviations for "Celsius" and corrected the sentence "*Concentration of RNA was measured on Nanodrop and isolated RNA was stored at -80°c for future use*" to "*Concentration of RNA was measured on Nanodrop and isolated RNA was stored at -80°C for future use*". Please see line 829, of the revised manuscript.

#20

Line 972: microarray datasets cannot be co-analyzed with RNA-seq data, since they are based on totally different technologies: microarrays 'see' only those miRs for which probes were printed, whereas RNA-seq detects every expressed RNA. This result must be deleted.

Here we referred to a smallRNA sequencing dataset from a mouse model for FTLD. This dataset is not based on microarray technology. The only microarray data we used was related to RNAseq and had accession ID GSE44770. We agree that a microarray is biased by probe design and that expression changes observed in an RNAseq experiment would not be detected if no corresponding probe is available. Moreover, RNAseq has a wider dynamic range. In our view, that does not mean that all previously published data that are based on array technology should be ignored and that these data cannot be compared to RNA-seq results. In fact, a substantial overlap amongst the genes that are detected as differentially expressed in a microarray and those detected by RNA-seq has been reported (e.g. see PMID: 30723492). Of course more genes are usually detected via RNA-seq. Nevertheless, we would argue that depending on the specific question, the results from a microarray analysis can be compared to RNA-sequencing experiments. In our study we employed several previously published datasets and we listed them under the section "Published datasets used in this study" with the material and methods section. We clearly highlighted if a dataset was generated via microarray analysis and only use them to compare lists of differential expressed transcripts as shown in former Fig 3H, now Fig 3I of the revised manuscript. Here, we report significant overlaps between the list of genes detected for example via microarray in previous experiments and our data. To address

this reviewer's concern we now more specifically explain our approach. Please see lines 327-329, 372-375 and 1164-1170 of the revised manuscript.

#21

Code shared for review and reproduction should adhere to best practices in biomedical programming, including sensible file names, functional organization, and commenting (as an unrelated example for good practice, compare here).

This is a general remark and we understand that this reviewer asks more specific questions within his/her comments 22-35. We agree that the level of detail should be sufficient for the reader to understand and reproduce data. Due to the interdisciplinarity of our study we might have missed some details relevant for this reviewer. This was unintentional and we apologize. We also appreciate data sharing in biomedical research. We have already shared the code and even the human data (to which some legal restrictions apply) with this reviewer at a "pre-revision-stage". Raw sequencing data will be available via GEO (non-human data). Human data will be available via the European Genome-Phenome archive (EGA, human data) upon publication. For now, human data is available via the editor upon signing a corresponding agreement. For now, all raw can be obtained from the editor. As for sharing the code please refer to our response to comment #22 by this reviewer.

#22

The currently presented analysis code is a collection of tutorial materials from the respective packages pasted together; however, one wonders why the structure- giving comments of the tutorial blueprints have been deleted by the authors.

For analysis we have employed published packages that are available via CRAN or Bioconductor and we provide the corresponding citations. Thus, the interested reader will be able to read the comments of tutorial blueprints. We describe how we performed the analysis and report the chosen parameters. We have now carefully revisited the analysis code and added more comments, when we felt it necessary. Custom source code along with processed data will be available via the following link upon publication (https://github.com/mdrezaulislam/paper_three_mir_signature). For now, source code along with raw data will be made available to this reviewer via the editor. Please note that, data access restriction apply to the human data due to legal obligations. This reviewer has already signed such an agreement.

The reviewer continues *"Similarly, some of the quality control steps, such as the visual controlling of WGCNA analyses, show problems with quality and were deleted from the script. Please revise and improve."*

This comment is related to previous issue raised in #14 by this reviewer. We did not delete any code in order to hide quality related information. We assume that the reviewer is referring to the whole network connectivity plot using plotDendroAndColors function that was missing for some analyses in previously provided code. This was not done intentionally. We have now carefully revisited and added this in the revised code.

#23

The authors use "auxiliary functions", which are not functions but rather simply lines of code in other, nondescript ("auxFuncxx.R") files, which further complicates retracing of the

analyses. It is recommended to source actual, parameterized R functions in these instances, which can be informatively read in the context of code, to avoid complicated code review.

This question was posed to us by the reviewer already during the “pre-revision” phase and we had addressed this issue. Thus, we had added the codes from previously defined auxFuncxx.R in the main code of the previously provided single PDF document (FischerRNAtherapeuticsHuman-main.pdf) that outlined each step along with the outputs. Please note that this document was provided in our pre-revision rebuttal.

We also addressed this issue in the revised provided code. Please see our response to comment 22 made by this reviewer.

#24

While the explanation of requested code in the form of a 87-page PDF file may have been well-intentioned, it requires manual copying of code into the R environment by the reviewer, and includes pages’ worth of unnecessary and uninformative R output.

We apologize for the inconvenience. We have now tried to minimize the R output with the aim to delete information that we consider uninformative and unnecessary.

This comment addresses, however, an important issue that might need some more general consideration.

To distinguish between information that is important or unimportant to understand and reproduce a bioinformatic analysis is to some extend subjective and also depends on the personal experience.

In fact, when we omitted comments for some steps (that we felt to be unimportant), this reviewer noted this as a potential problem (see his/her comment 22).

As discussed in our response to comment 22 we now provide the requested source code along with raw data to reviewers, so they can reproduce the findings that we present in our manuscript.

Code-based re-analysis

#25.

Figure 1: The total output of only four modules is very uncommon for a WGCNA dendrogram (below); particularly in miRNA expression, which is often complex.

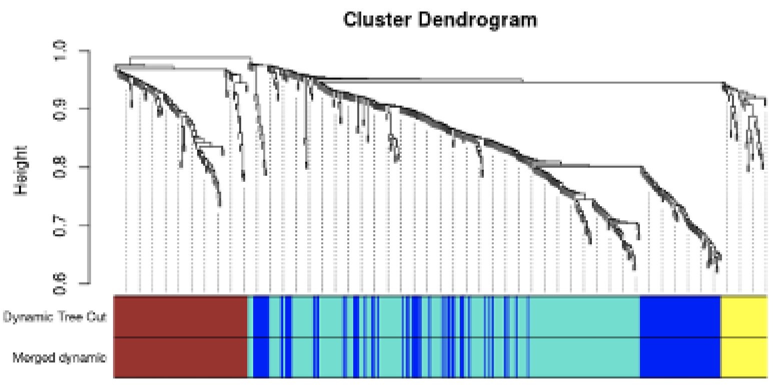

WGCNA parameters can be iterated for robustness in this case, to exclude chance results (as in this example*). The merge step performed in the code is not necessary.*

The detected number of modules may vary due to the choice of different parameters (e.g., module size, deep split size). However, we thank the reviewer for this remark to perform additional analyses. We have now applied similar additional steps using various WGCNA parameters and found that the detected modules particularly blue and brown modules are robust across different parameters. Of note, blue and brown modules contain the described three microRNA signatures presented in this study. Please see the corresponding code, revised Fig.1, novel Expanded view Fig. 3 and their corresponding legends. Please also refer to lines 162-163 and 973-974 of the revised manuscript.

#26.

Re-alignment of the supplied raw fastq files, variance stabilization, and WGCNA analysis found 11 co-expression modules; however, none of those correlated with cognitive status or sex. This discrepancy should be re-assessed. Further, the code used for alignment and preprocessing (which was not available for review) as well as this result should be reconsidered critically, with particular attention to quality control steps.

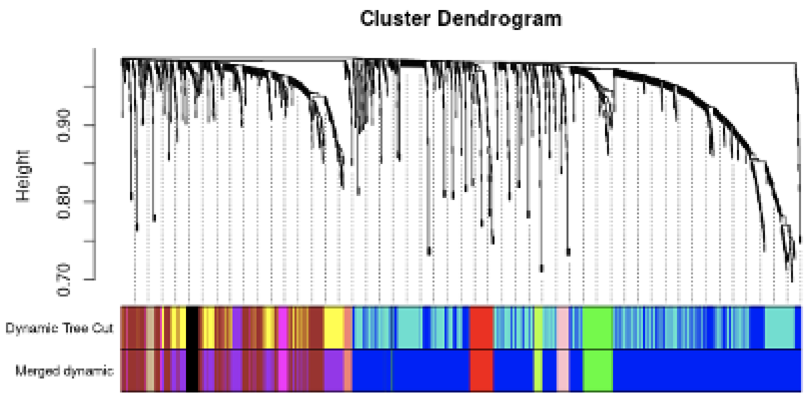

We understand that this reviewer used the data we presented withing Fig.1 to perform an independent analysis. At present we cannot judge this analysis and do not know to which co-expression modules he/she is referring. We have reported 4 modules. We extend the method part for mapping and alignment steps in the revised manuscript. We have also added the

filtering and quality control steps in the corresponding code (see also our response to comment 22) that will allow every reader to reproduce the results reported in the manuscript.

#27.
The (original) correlations of eigengenes to cognition are very shallow (Pearson correlations between 18 and 25%, see below).

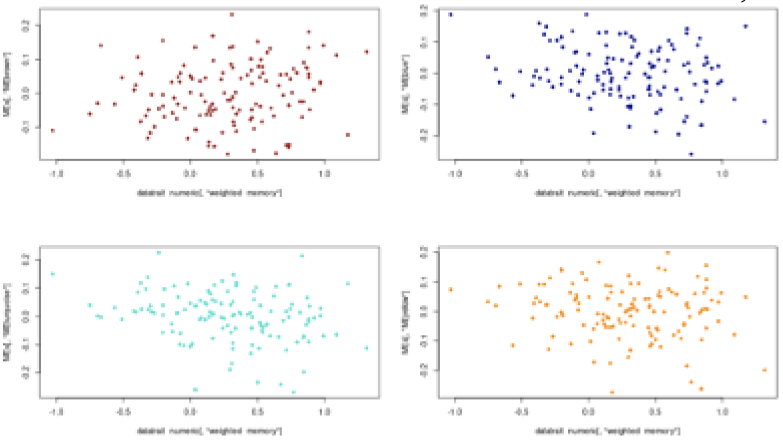

We would like to reiterate that we analyzed healthy and young individuals that do not suffer from any cognitive disease. Therefore, the moderate -yet significant - correlation of the microRNA modules with cognitive performance is not surprising. In fact, our findings are in agreement with a comparable study in which moderate genetic association between general cognitive function in healthy individuals and longevity was observed (e.g. PMID:29844566)

The reviewer continues *"Figure 1C shows only p-value asterisks, although there would be ample space for displaying numeric values. This mode of presentation limits one's confidence in the claimed large predictive value of circulating miRs on cognition in healthy, young individuals."*

We have improved the presentation and now indicate numbers in addition to the asterisks. Please see the revised version of Fig.1 We also like to specifically respond to the statement: "*limits one's confidence in the claimed large predictive value of circulating miRs on cognition in healthy, young individuals"*

There seems to be a fundamental misunderstanding regarding the way our study is viewed by this reviewer. Our aim was to detect microRNAs as candidate biomarkers for the early detection of individuals at risk to develop dementia. Therefore, we outlined a multi-step experimental approach and specifically mention that the experiments this reviewer is referring to here, are a "starting point" and that the corresponding results need to be followed by more specific experiments, which we then conducted. We described this before. Please see now lines 140-147 of the revised manuscript.

#28.
That all WGCNA modules identified by the authors are very similar in their function is very surprising. Module sizes are not given in the manuscript, but upon re-running the code, the following module sizes emerge: blue 115 miRs, brown 94, turquoise 217, yellow 32. According to this analysis, as much as 93% of blood cells miRNAs are all supposedly involved with cognitive processes and only about 7% of miRNAs do ***not*** *correlate with cognitive performance (which is rather unlikely).*

In the revised version of the manuscript we now provide within Fig. 1 C and the corresponding figure legend the module sizes.

For the suggested analysis of how many microRNAs are potentially linked to cognitive function, the analysis should rather start with the total number of microRNAs initially used for the WGCNA analysis. Here, we have employed 456 microRNAs (minimum expression of 5 reads in 50% of the samples) of which 212 are found the 3 clusters. Even this does not mean that all of these microRNAs are indeed linked to cognitive function and therefore we performed the subsequent experiments described within Figs 2-7.

#29.

The prominent identification of age-related processes in all modules is likely a result of confounding of the gene ontology analyses, possibly by filtering for only brain- expressed genes. "Confirmed mRNA targets" likely means that the authors used experimentally validated interactions from miRTarBase in these analyses. This could likewise lead to confounding, as validated miRNA interactions are extremely biased and reflect only a very small subset of all true interactions. On the other hand, predicted interactions are notoriously incorrect and yield a large number of false positives. A state-of-the-art publication on miRNA interactions should address these shortcomings in a quantitative manner, as has recently been demonstrated multiple times. The process of miRNA targeting analyses, gene filtering, and GO analysis was not reported in detail (e.g., what was the source of "brain-expressed" genes) and is not available in the supplied code. These analyses should be critically re-examined and described in sufficient detail for enabling in-depth review and replication.

The comment is similar to his/her comment #5. We like to reiterate that any analysis which is purely based on the mentioned bioinformatic predictions can only be a first step, with the intention to guide further experiments. We now state this even more specifically in the revised version of our manuscript. Please see lines 168-178.

To furthermore address this reviewers concern, we re-analyzed the data more „critically". Although we disagree with this reviewer's view, in the revised GO analysis we did not filter the data for microRNAs detected in the brain. We also describe the GO-analysis in greater detail. The message we communicate remains similar as reported before. Please see lines 168-178, 1006-1014 and appendix table 3

#30.

*Figure 2, differential expression: "Unwanted variation" is removed via RUVSeq; however, the negative control genes used for this algorithm are **all genes in the expression matrix**. The reason for this is unclear, no rationale is given.*

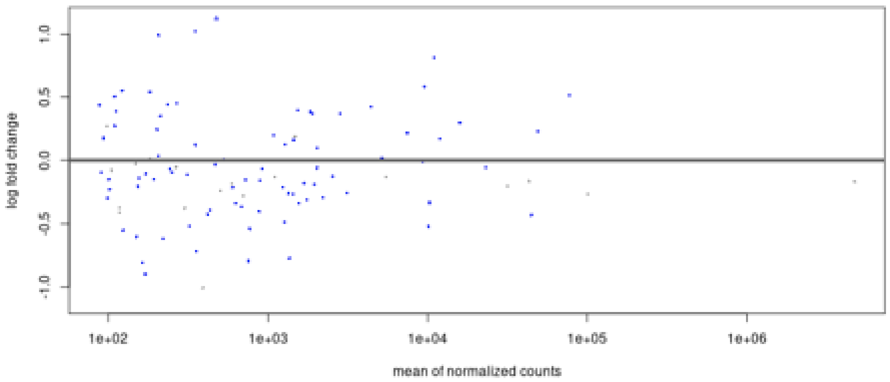

Unwanted variation is removed according to the documentation of the RUVSeq package. The authors discuss multiple possibilities to determine and remove unwanted variation from sequencing data. In this study, the replicate samples from each time point were used to determine the unwanted variation (for reference to the approach please see section 3 of https://bioconductor.org/packages/release/bioc/vignettes/RUVSeq/inst/doc/RUVSeq.pdf). We have extended the Methods section accordingly. See lines 894-896.

Reviewer 2 continues: *Additionally, the expression matrix is heavily filtered (more than 100 counts in at least half the samples), which is not recommended by the authors of DESeq2, and greatly decimates the already small pool of initial miRs. The MA plot of the differential expression is highly irregular, with almost every studied miR differentially expressed (below).*

We pre-filtered microRNAs prior to differential expression analysis as microRNAs having average reads of more than 100 are highly correlated in expression between NGS and qPCR platforms (please check PMID:28439824).

Filtration of lowly expressed microRNAs/genes prior to differential expression analyses is common practice to ensure the detection of robust changes (PMID: 25150836). Moreover, we did not find any specific statement in DESeq2 vignette to suggest that our approach would be fundamentally wrong.

Thus, there are different opinions on this issue that are in our view all valid but need to be considered in light of the specific scientific question that is addressed. Our aim was to detect microRNAs that can be assayed as biomarkers. Since, the ultimate aim is the development of a point-of-care assay that is independent of sequencing approaches (now lines 690-692), we reasoned that the detection of changes amongst lowly expressed microRNAs would be suboptimal which is in line with previous data (PMID:28439824).

To nevertheless address this reviewer's concern, we re-analyzed the data without 'heavy filtering'. This had no substantial effect on the message we communicate in our study. We summarize this result within novel Expanded View Fig 5D, its legend and refer to the issue in the revised manuscript. Please see lines 250-254.

#31.

Simple quality control such as mean-SD plots for the count data should be performed routinely in the course of analyses. The mean-SD plots for the filtered counts used in the authors' analysis (left) and the variance-stabilized, unfiltered count data (middle) differ significantly from regular examples of mean-SD plots from the DESeq2 vignette (right). In the case of the authors' original analysis, the prior assumptions of DESeq2 probably do not hold.

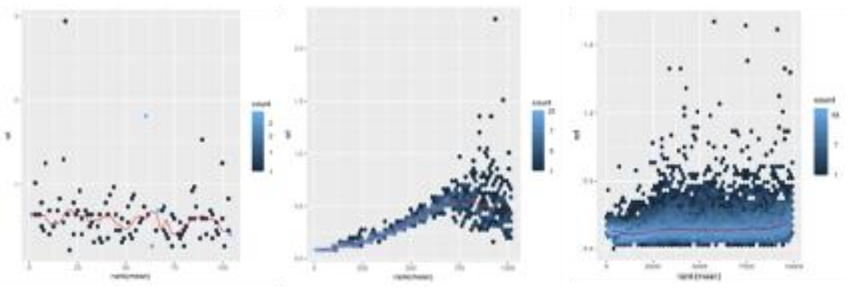

We are of course aware of these QC steps and routinely perform them.

With respect to the provided image above we like to refer to these data as left (LP), middle (MP) and right panel (RP) in the following.

Importantly, this reviewer compared our microRNA expression data (LP) with the datasets described in the DESeq2 vignette, which represent RNAseq data (MP and RP). Generating the mean-SD plot for the RNAseq data we describe in Figure 3 leads to a very similar pattern to that of the RP (See image below).

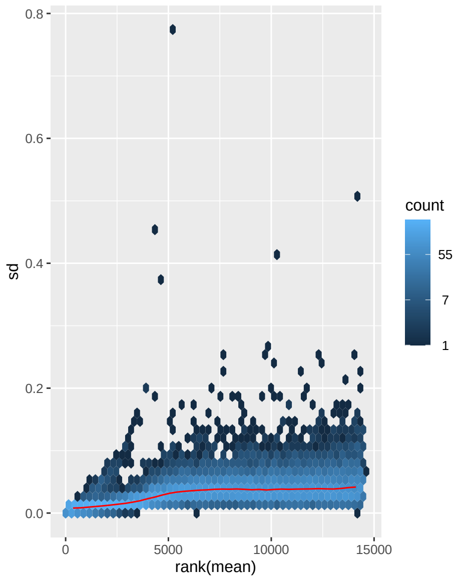

Figure legend.. Mean-SD plot for RNAseq data related to miR-146a-5p overexpression experiments from Figure 3E.

We like to point out that the LP-plot is not comparable to the other data (MP & RP), simply because they are showing the effects of the normalization step on two different species of RNA, microRNAs and mRNA's, respectively.

Aside from the broken red line in the LP-plot (which is an artifact due the function used to generate the mean-SD plot) one can see that the variance-stabilization (vst) step is making the microRNA data comparable across the whole range of microRNA's: highly expressed

microRNAs (high mean) have roughly the same SD as lowly expressed microRNAs (low mean), explaining why most of the 'dots' center around the vertical range of [0,1]. This is what the vst normalization aims for and it is doing it reasonably well.

In addition, we have also analyzed a published microRNAseq dataset (GEO id GSE46579) that is completely independent to ours. As shown below, this data also confirms that the pattern for microRNA is substantially different from that of RNAseq, simply due to the difference in the numbers of RNA features (for microRNA: 300-700; for mRNA: 16000-20000) being studied. Please note again, that the majority of the 'dots' are located within the range of [0,1] on the vertical axis in the figure below.

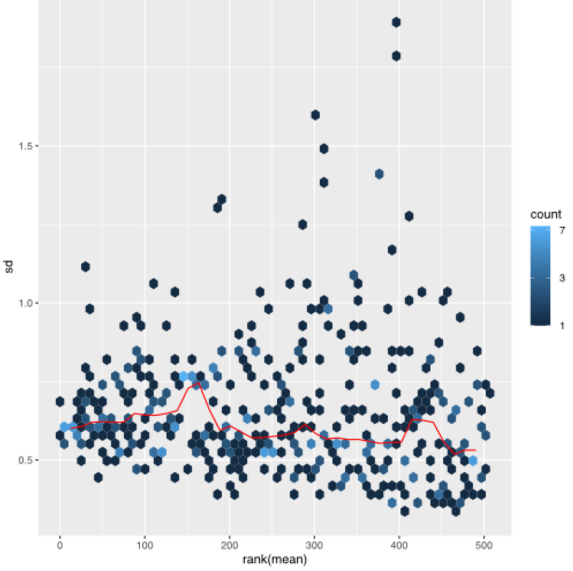

Figure legend: The above figure shows the mean-SD plot for microRNA expression retrieved from GSE46579.

#32

Downstream analyses based on the same data, namely the feature selection (2F) and eigenexpression in the time course (3A), may also be deeply affected by these irregularities. These analyses should be repeated adhering to the basic principles of biostatistics.

We understand that this concern is based on "*irregularities*" this reviewer has spotted and outlined within his/her comment 30 and 31. Since we are confident that we have now sufficiently clarified these issues, this comment is no longer relevant. Please refer to our answer to comment 31 and 32 by this reviewer.

#33.

The authors preselected three out of 145 miRNAs (the reason for choosing these 145 is not given) out of the human data, calculated eigenexpression values, performed statistical testing (Figure 3B) and compared the eigenexpression values using the standard Mann-Whitney test used in stat compare means(). However, this is a simple nonparametric test without multiple correction, although correction for multiple testing is an absolute must in this case. The potential number of three-miRNA combinations in only those 145 miRNAs used is nchoosek(n=145, k=3) = 497640.
Arguably, all miRNAs should be used, not only those 145, but that yields an almost sex

number of combinations. Randomly selecting 12 three-miR-combinations (by setting set.seed() to 1-12) and repeating the authors' analyses for each combination resulted in 7 combinations with significant predictive capacities, five of those with better separation power than the three-miR-signature reported by the authors (below). This is very obviously a statistical artifact caused by the lack of correction for multiple testing. All similar analyses should hence be repeated with adherence to basic statistical principles.

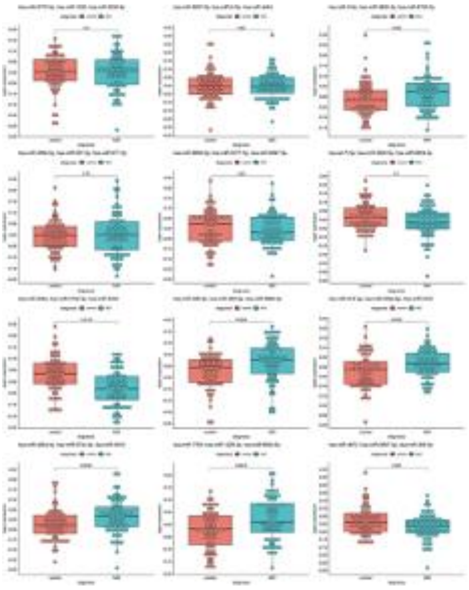

General response:

This is an interesting comment. However, as already addressed in our response to comment 27, we are really concerned that there is a fundamental misunderstanding of our manuscript. We outlined in great detail that in this study we wanted to test a novel approach to identify microRNAs as biomarkers for the early detection of people at risk for developing dementia. We specifically stated, that in our view it might be difficult to find such marker starting the analysis with already diagnosed patients. Thus, our aim was not to use a cohort of MCI and control patients with the aim to apply some bioinformatic approaches and then communicate to the field without further information another long list of candidates that differ significantly between patients and controls. Therefore, please note that the analysis of the DELCODE dataset is not a discovery cohort but used to test the expression of our curated 3-microRNA signature.

What this reviewer is suggesting to us, is essentially to write a completely different manuscript using the classical approach to employ a cohort of patients and controls with the aim to find differences amongst them. We will not do this! Rather, we encourage him/her to use our raw data upon publication and to perform his/her own analysis as desired and present the corresponding data in an independent manuscript.

Technical concerns.

Having this said, the technical concerns raised here become obsolete but we like to nevertheless briefly address them.

The reviewer is pointing out that some of our analysis lacked correction for multiple testing, particularly where the mean values of the eigen-expression of two groups is compared (Fig 5B). In cases like those of Fig 5B, where comparisons of two means were carried out, we ran

permutation tests (which are inherently non-parametric) with multiple adjustments to validate the p-values obtained from the Wilcoxon test and found similar statistical significance.

Regarding the feature selection step, we would like to make some clarifications. First, let us touch the point of the potential sizes of the different miRNA signature spaces. The number of ways one can select k=3 objects out of a pool of approximately n=2000 objects (the size of the annotated mouse miRNAome) is nchoosek$(n = 2000, k = 3) \approx 1 \times 10^9$, which is far less than ‘infinite’. Neither it is infinite the number of all possible signatures of size ‘k’ that can be searched across the whole miRNAome, that is $\sum \quad nchoosek(n, k) = 2^{2000} \approx 1 \times 10^{600}$; this is a number even bigger than the estimated number of atoms in the universe, but still well bounded and countable (in set theory sense). If we just took into account the miRNAs this reviewer is referring to, the total number of possible signatures of size ‘k’ would be $\sum \quad nchoosek(n, k) = 2^{145} \approx 4 \times 10^{43}$, because there is no prior to lead us think the signature has to be specifically of size k=3.

This last search space, with 4×10^{43} possible signatures, is the most realistic to consider, given that the filtering step is necessary to trim out miRNAs that have essentially no expression. Nevertheless, exploring this space is prohibitive even with today’s fastest computers. This is the reason for us to rely on strategies to optimize the search of such big a space, and one such strategy is recursive feature elimination (RFE), which is a greedy approach.

Now, is it necessary to do correction for multiple testing when it comes to the application of RFE and similar strategies? Are we at risk of finding a spurious signature because the search space is huge? In reality, there is no consensus in the literature. For example, Whittingham et al. (2006) and Smith (2018) argue against the application of these type of strategies because they may lead to overlooking variables (miRNAs) that are truly important and consequently, lead to inferring the ‘wrong’ model. On the contrary, Rothman (1990) and Perneger (1998) give an opposite point of view, among other things because p-value correction only controls for Type I error at the expense of Type II. We believe Greenland (2008) give a more sober and balanced view on this topic. The examples here provided do not constitute an extensive list.

- Smith, G. Step away from stepwise. *J Big Data* **5,** 32 (2018). https://doi.org/10.1186/s40537-018-0143-6
- Whittingham, M. et al. Why do we still use stepwise modelling in ecology and behaviour? J of Animal Ecology, 75, 5 (2006). https://doi.org/10.1111/j.1365-2656.2006.01141.x
- Rothman, K. J. (1990). *No Adjustments Are Needed for Multiple Comparisons. Epidemiology, 1(1), 43–46.* doi:10.1097/00001648-199001000-00010
- Perneger, T.V. What's wrong with Bonferroni adjustments. BMJ 1998; 316 : 1236
- Sander Greenland, Multiple comparisons and association selection in general epidemiology, International Journal of Epidemiology, Volume 37, Issue 3, June 2008, Pages 430–434, https://doi.org/10.1093/ije/dyn064

We are from the stance that no feature selection procedure is perfect. Yes, from an algorithmic point of view, we may be overlooking some potential combinations out there. However, in order to reduce the chance of making both type 1 and type II errors, it's recommended to evaluate all evidence supporting the inferred model, instead of focusing only

on statistical significance. Moreover, results from a single analysis (like RFE) should not be used to make treatment decisions; instead, one should look for scientific plausibility and supporting data from other studies which can validate the results of the original study. A couple of examples supporting this point of view are Buyse, Pradesh and Ranganathan (2006) and Feise (2002), which can be found respectively in here:

- Ranganathan P, Pramesh CS, Buyse M. Common pitfalls in statistical analysis: The perils of multiple testing. *Perspect Clin Res.*2016;7(2):106-107. doi:10.4103/2229-3485.179436
- Feise, R.J. Do multiple outcome measures require p-value adjustment?. *BMC Med Res Methodol* 2, 8 (2002). https://doi.org/10.1186/1471-2288-2-8

Given the above arguments, we reasoned that if the expression of the selected 3-microRNA combination in one dataset is observed only by chance or simply due to artifact, it's highly likely that the expression pattern of the given 3-microRNA combination would be highly inconsistent across datasets and the expression pattern would be irreproducible in independent experimental settings. To rule out the potential effect of artifacts and address the specificity and relevance of the selected 3-microRNA signature in cognition and to comply with recommended statistical guidelines, we tested the signature performance in two independent approaches

1. We had performed a meta-analysis reporting the effect size across multiple relevant datasets (at least 14 datasets) for the given 3-miRNA signature and now also compared its performance with 1000 random combinations of 3 microRNAs selected from the 55 microRNAs reported in Fig 2E or from the detected human microRNAome. The experimentally curated 3-microRNA signature reported in our manuscript outperforms all the various combinations tested including the 7 random ones from the reviewer pointed out (see below), highlighting the robustness of our 3-microRNA signature.
These results are summarized in novel Fig 5I, Expanded View Fig 12, 13, Appendix Table 13, 14. Please note that in the meta-analyses, the reported p values are adjusted with multiple corrections.

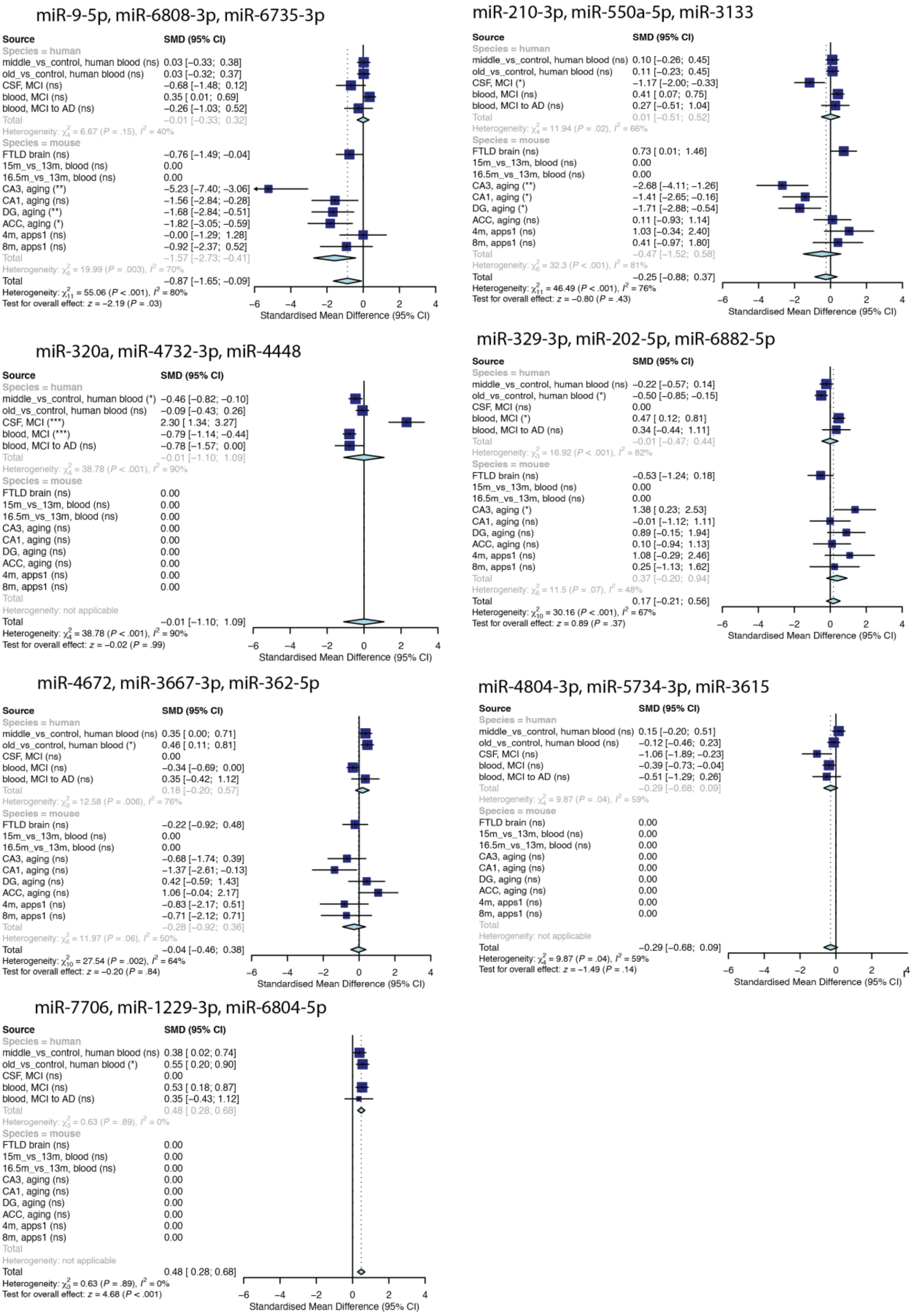

Figure legend. Meta-analyses performed using 7 random combinations of 3-miRs human microRNAs detected in DELCODE.

Meta-analyses were performed (see methods for details) for 7 random 3-miRs combinations of human microRNAs detected in the DELCODE cohort. The dataset related to qPCR array (plasma, MCI) was excluded from the analysis due to its technical limitation. Thus, the meta-analyses were performed on remaining 14 datasets. The systematically curated 3-miRNA signature described in this study (see also Fig 5I) outperforms all the seven random combinations tested in terms of significant overall effect (for 3-miRNA signature in 14 datasets, the overall effect: 5.25, P value: 1.55E-07). Adjusted p values across studies are summarized in the parentheses next to the study name.

2. For more conclusive scientific evidence on the relevance of the selected 3-microRNA signature, we have performed experiments in various models to test the link of the selected 3-micoRNA combination to cognition (see Fig 3, 4, 5G-H, 6, 7). It is beyond our scope to experimentally test the 497639 other different combinations of 3-microRNA signatures. Here we like to refer again to our "general response". <u>The DELCODE cohort is not used as a discovery cohort in our study.</u>

In summary, we demonstrate the reproducibility of our data in independent datasets and provide experimental validation to support the observation, which empirically demonstrates that our study is reasonably well-powered, rigorously planned and follows the recommended robust statistical guideline.

34. Not excluding the excluded samples (due to unique center or missing nationality, see also point 17) also leads to a non-significant difference (p = 0.05) in this comparison. The excluded samples are either MCI with very low values, or controls with high values (below). It is not possible to control exclusion criteria, because nationality and center are not given in the metadata.

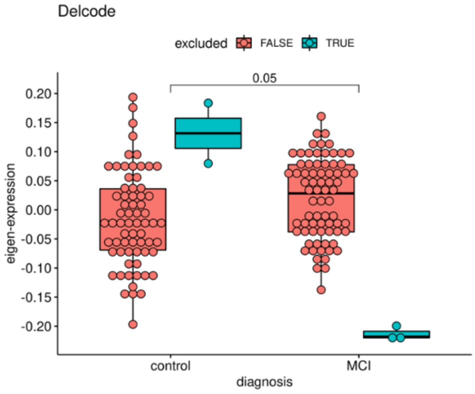

As described in the previous version of our manuscript, we excluded these individuals, since "unknown nationality" and "unknown center" is automatically removed from analysis of the study since it may indicate potential problems with the handling of the samples.

However, to address reviewer´s concern and to avoid any potential impression of ineptitude as he/she mentioned, we have now reanalyzed these data using fully automatic detection of outlying samples based on Z score. Samples with low quality Z score (Z > 2.5 or Z <-2.5) of eigenvalue were filtered out for downstream comparative analysis between conditions. Thus, we found three outliers in this particular dataset. Those were now removed from the revised analysis. Please note, that same outlier detection step has been now implemented for all the data analyzed. Importantly, the message communicated in our manuscript does not change and in fact the 3 outliers are MCI patients with particularly low expression of the 3

microRNA signature that did not convert to AD. We furthermore address this issue now in the revised manuscript. Please see lines 382-385 and 908-910 of the revised manuscript.

35. Note: Due to numerous problems in these first three figures, further examination of code was not performed.

We are confident that we sufficiently addressed all concerns raised by this reviewer and furthermore like to point out that several of these concerns were due to a misunderstanding of our approach (e.g. see our response to comment #27 and #33), which we have now addressed in detail Moreover, this reviewer did not stop examining the code after the first 3 figures but specifically re-analyzed data that involved RNA-sequencing approaches and formulated very detailed technical questions related to Figs.1, 2, 3, 5.

Referee 3

Major concerns:

Cohorts:
Subjects have a different cognitive abilities - authors should address the concern that variables such as IQ, education and demographics can act as a confounder to cognitive status at baseline.

We thank the reviewer for this remark. We now provide in revised Fig 1C data to show that years of education did not affect the results. Please also see the corresponding figure legends and lines 165-167 of the revised manuscript. We do not have data regarding the IQ but we like to stress the fact that even if difference in IQ would underlie -at least in part -the observed variability in the cognitive tests, this would not affect the interpretation of our data. The aim of this analysis was to identify microRNAs that correlate with cognitive abilities in otherwise young and healthy humans, which is of course only possible since there is variability amongst the individuals.

Are individuals from different groups (control, MCI, and MCI converted to AD) comparable in terms of IQ, demographics, education etc?

We thank reviewer for this remark. The microRNA expression for the corresponding data has been adjusted for latent variables. Please see lines 890-897 of the revised manuscript.

Computation and machine learning:
Authors machine-learning approaches suffer from limitations that should be corrected:
Performance matrices should be reported on each occasion machine learning is utilized, for expert reader in the field to be able to assess the method and to draw meaningful conclusions from the analysis.
Authors should report the accuracy of the ML regression algorithms
Authors should explain the validity of the selected features, which is currently limited.
The exact magnitude of the main correlations should be reported.
Authors should characterize the predictive power and quantitative parameters of miRNA signature including specificity and sensitivity, concentration cutoff.

We thank the reviewer for providing such helpful comments. It should be noted, however, that these analyses were based on doing regression and not classification, therefore performance figures like accuracy, specificity, sensitivity do not apply, neither confusion matrices nor cutoff levels. We have made this clear in the revised manuscript (line 989-990).

As a first mean of determining the validity of the selected features, we estimated the probability that 7 coinciding miRNAs had been selected by chance by three independent algorithms: Random forest trained with bootstrapping; Random forest trained with leave one out cross-validation and SVM trained with bootstrapping. Probabilities were estimated through permutation tests. In none of the simulations, the estimated frequencies exceeded the 0.05 value commonly used for thresholding p-values. The details are as follow:

Figure 2F shows that seven common miRNAs were selected as top features by three different algorithms: random forest trained with bootstrapping (rf.boot); random forest trained with leave-one-out crossvalidation (rf.loocv) and a support vector machine trained with bootstrapping (svm.boot). Is this event a product of chance?

This problem can be cast as that of having three different people drawing out balls from an urn containing "*N*" balls and checking which of those drawn balls were the same. The number of balls taken by each person could be fixed to a common number "*n*" or it could be different for each one of them, i.e. n_1, n_2 and n_3. Each person takes the balls independently from the others and with replacement. This experiment can be repeated a number of times.

The simulation was done using two different urns, one with N=2000 balls, which is the approximate size of the documented mice miRNAome and N=55, which are the number micro RNAs used for the feature selection analysis. The number of balls drawn by each person (algorithm) was set to n=55 (i.e. all people draw the same number of balls) or to $n_{rf.boot}$= 20, $n_{rf.loocv}$= 16 and $n_{svm.boot}$= 17; where the latter are the number of features one would select from the recursive feature elimination algorithm if the RMSE were to be minimised (see Fig. ES2). For each parameterisation, the simulation was carried out 100,000 times.

As shown in the Table below, the probability of drawing these seven miRNAs is extremely low. We acknowledge nevertheless, that in this exercise two of the people (or algorithms) are not completely independent from each other, the random forests trained with LOOCV and bootstrap respectively. Nevertheless, doing a similar simulation using only two people yields similar results.

**Parameters:**<br>**3 urns**	**p-value**
Urn size N=2000<br>Drawn balls: n=55<br>Overlap size = 7	0.00000
Urn size N=2000<br>Drawn balls: $n_{rf.boot}$= 22, $n_{rf.loocv}$= 16 and	0.00000

$n_{svm.boot}$= 17<br>Overlap size = 7	
Urn size N=55<br>Drawn balls: n=20<br>Overlap size = 7	0.00459
Urn size N=55<br>Drawn balls: $n_{rf.boot}$= 22, $n_{rf.loocv}$= 16 and $n_{svm.boot}$= 17<br>Overlap size = 7	0.00047
Urn size N=55<br>Drawn balls: $n_{rf.boot}$= 10, $n_{rf.loocv}$= 10 and $n_{svm.boot}$= 10<br>Overlap size = 7	0.00000
**Parameters: 2 urns**	**p-value (frequency)**
Urn size N=2000<br>Drawn balls: n=55<br>Overlap size = 7	0.00000
Urn size N=2000<br>Drawn balls: n=20<br>Overlap size = 7	0.0000

Regress miRNAs on the cognitive decline slope in healthy subjects, if you can gain such data.

No longitudinal data is available for the healthy subjects. However, we had access to cross-sectional data from healthy subjects at different age groups. We presented these data within expanded view Fig 10, showing that the 3-microRNA signature increased in expression as memory declined with advanced age.

Authors acknowledge in discussion that they should have better performed longitudinal study on humans and not only on mice, but they should hopefully find a way to approximate this gap.

We believe that our wording was misleading. Thus, as outlined for example in Expanded View Fig 1, our experimental approach differs from other biomarker studies in order to address this issue. Our specific aim was not to find marker that differ between control, MCI or AD patients but to identify candidates suitable for early detection, without the need to wait for suitable longitudinal data that may become available from epidemiological studies in the future. Combining the analysis of young and healthy humans with the longitudinal analysis in animal models and a functional analysis followed by the testing of candidates in cross-sectional human data is in our view a first step to approximate the „*gap*“ referred to here.

At the same time, we agree with this reviewer and like to mention that we are following the suggested approach. For example, we are performing smallRNA sequencing form individuals of the DZNE Rhineland study (https://www.rheinland-studie.de) that enrolls individuals at the age of 30-80 which then undergo longitudinally phenotyping with a focus on cognitive function. We have sequenced so far 3000 individuals at baseline and will be happy to report results once meaningful longitudinal data will become available. Of particular interest would be the analysis of middle-aged individuals that will eventually develop for example SCI, MCI or AD. Such data will however not become available in the near future. We address this issue now in greater detail within lines 578-581 of the revised manuscript.

Mouse models:
Harmonization required, to be gained by testing similar endpoints (memory performance, neuronal function) with miRNA mimics and miRNA inhibitors.

We assume that this reviewer refers to the fact that we administered microRNA mimics to recapitulate the disease situation in the cell culture experiments described in Fig 3 and 4, while we administered microRNA inhibitors to test therapeutic efficacy in models for age-associated memory decline and amyloid pathology as shown in Figs 6 and 7. The data shown in Figs. 3 and 4 served the purpose to further curate the candidate microRNAs before testing their performance for example in MCI patients as shown in Fig 5. In contrast, the data shown in Figs 6 and 7 aimed to test the therapeutic potential of the 3 microRNA signature in relevant model systems. To perform now a detailed analysis of the 3 microRNAs and their role in neuronal function using gain and loss of function systems *in vivo* and *in vitro* is an interesting questions, but is certainly beyond the scope of our manuscript.

However, we had already thought about the questions formulated by this reviewer and initiated a novel project. The data will be presented in an independent manuscript most likely in 2-3 years from now.
Nevertheless, we like to share a set of preliminary data with this reviewer to illustrate that we indeed plan to follow this line of research in the future. Thus, we have injected for example microRNA mimics for miR-181a-5p into the hippocampus of young wild type mice and compared their performance in the contextual fear conditioning paradigm, a simple one trail hippocampus-dependent learning paradigm we often use prior to a proper water maze experiment when the aim is to initially test a hypothesis. As can be seen form the image below, our data indicates that acute injection of miR-181a-5p leads to memory impairment in this test. We also did a first experiment employing the water maze test. These data are of course preliminary. Nevertheless, they are in agreement with our data shown in Fig. 3 and 4 suggesting that miR-181a-5p may mainly affect neuronal functions, which would explain the immediate effect on memory performance assayed 1 week after the injection of miR mimics. We will follow this line of research - also for the other 2 microRNAs reported in our study - in future research.

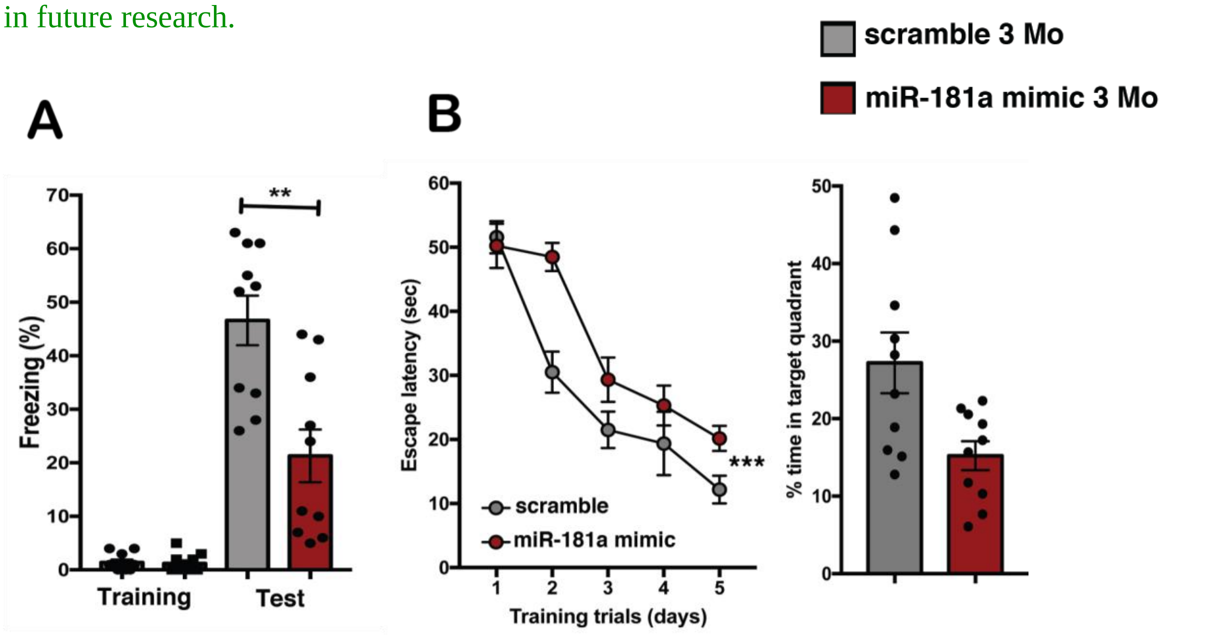

Figure legend: *3 months old mice (male) were injected with miR-181a mimic or scramble into CA of hippocampus and its effect on memory was evaluated through behavioral performances (e.g., fear conditioning*

*and water maze test). (A) Mice treated with miR-181a mimic displayed significantly fewer freezing episodes compared to control group, displaying poor memory recall (Two-way ANOVA, **P<0.01) (B) In Water maze test, (left panel) miR-181a mimic injected mice took more time to find the platform during training sessions (Two-way ANOVA, **P<0.01). During probe test, (right panel) mimic treated mice spent less time in target quadrant compared to control mice (unpaired t-test, two-tailed, P = 0.01). n = 10/group.*

Authors had tested the simultaneous introduction of miRNA mimics or miRNA inhibitors for all miRNAs. However, miR-146 and miR-181 negatively correlates with cognition (Figure 2G). Authors are asked to explain or correct the experimental design: should cognition impairing perturbation be to combine overexpression mimics of miR-146 and miR-181 with a miR-148 inhibitor? Should cognition enhancement be driven by miR-146/181 inhibitors together with miR-148 overexpression?

This reviewer refers to our data presented within Fig. 1. Please note that the data presented here should be viewed as first experimental approach to identify candidate microRNAs for further analysis. Thus, we cannot exclude that the presence of microRNA-148a-3p in the brown cluster may reflect some form of compensatory mechanisms. Our subsequent analysis shown for example in Fig 3 and 4 suggest a detrimental effect of elevated microRNA-148a-3p levels. To further address the issue raised by this reviewer, we have now repeated the experiments shown in Fig 4, and now target all three microRNAs individually. The corresponding data is presented within novel Expanded Fig. 9B-C and are mentioned within lines 351-352 of the revised manuscript. In summary we show that increasing the levels of all 3 microRNAs impairs synaptic plasticity, measured via spine density and MEA assays. We now also refer specifically to this issue raised here in the revised version of our manuscript within lines 513-519.

Brain regions studied:
The manuscript focuses on the hippocampus and reports only one cognitive test in mice, which reflects mostly hippocampal function. This should be justified and better if analysis would have been expanded. In this context Wolf et al. may be referenced (Hippocampal volume discriminates between normal cognition; questionable and mild dementia in the elderly. Neurobiol. Aging 22, 177-186. 10.1016/s0197-4580(00)00238-4) and also Dicks et al., (NeuroImage: Clinical 22, 2019, 101786 10.1016/j.nicl.2019.1017860) who found a widespread atrophy pattern with the strongest associations for decline over time for the bilateral hippocampi, insulae and Rolandic opercula (NeuroImage: Clinical 22, 2019, 101786 10.1016/j.nicl.2019.1017860). In contrast, a recent study suggests the precuneus and inferior temporal regions as key regions in physiological and pathological brain aging (Lee et al., Front. Aging Neurosci. 2019 10.3389/fnagi.2019.00147), to make a point that the regions studied in vivo and in vitro should be thoroughly justified. Are the findings relevant to hippocampus-dependent function and not to cognitive function in general?

This is a very insightful comment. As suggested by this reviewer we now provide a more detailed rational for analyzing the hippocampus and hippocampus-dependent memory function, referring also to the suggested literature. Please see lines 190-194, 458-463 492-493 and 670-673 of the revised manuscript. In addition, we like to communicate to this reviewer that we are already planned further experiments as implicated by the question of this reviewer. Namely, we aim to test in the future the hypothesis that changes in the circulating microRNA signature inform about structural and functional changes in the human brain. For example, we have successfully submitted an application to the ADNI consortium and are currently sequencing 847 blood samples from control, MCI and AD patients that were collected at baseline and for whom substantial amount of structural and functional imaging

data is available. This is an ongoing project that we aim to report within the next 2-3 years as an independent publication.

16th Aug 2021

Thank you for the submission of your revised manuscript to EMBO Molecular Medicine. We have now received the enclosed report from the two referees who agreed to re-assess it. As you will see, the referees provide enthusiastic support, and I am pleased to inform you that we will be able to accept your manuscript pending the following amendments:

I look forward to receiving your revised manuscript soon.

Best wishes,
Jingyi

Jingyi Hou
Editor
EMBO Molecular Medicine

***** Reviewer's comments *****

Referee #2 (Remarks for Author):

I find the extensively revised version of this manuscript to be far clearer and better focused compared to the previous version. I thank the authors for their patient and careful response to each and every one of the detailed comments of the three reviewers which obviously reflect deep interest in the challenging research question that this paper addresses, and wish them great luck in the extension project described in their response.

Referee #3 (Comments on Novelty/Model System for Author):

I think the manuscript is improved and is important the the Molecular Medicine community.

Referee #3 (Remarks for Author):

The manuscript of Md Rezaul Islam et al., has been improved since the original submission.
The data are sound, conclusions novel and I believe that the discoveries are important the the Molecular Medicine community.

The authors performed the requested editorial changes.

24th Aug 2021

We are pleased to inform you that your manuscript is accepted for publication and is now being sent to our publisher to be included in the next available issue of EMBO Molecular Medicine.

We would like to remind you that as part of the EMBO Publications transparent editorial process initiative, EMBO Molecular Medicine will publish a Review Process File online to accompany accepted manuscripts. If you do NOT want the file to be published or would like to exclude figures, please immediately inform the editorial office via e-mail.

Please read below for additional IMPORTANT information regarding your article, its publication and the production process.

Thank you for submitting this interesting work to EMBO Mol Med.

Best wishes,
Jingyi

Jingyi Hou
Editor
EMBO Molecular Medicine

Follow us on Twitter @EmboMolMed
Sign up for eTOCs at embopress.org/alertsfeeds

EMBO PRESS

YOU MUST COMPLETE ALL CELLS WITH A PINK BACKGROUND ↓

PLEASE NOTE THAT THIS CHECKLIST WILL BE PUBLISHED ALONGSIDE YOUR PAPER

Corresponding Author Name: Andre Fischer
Journal Submitted to: EMBO MOL MED
Manuscript Number: EMM-2020-13659

USEFUL LINKS FOR COMPLETING THIS FORM

http://www.antibodypedia.com
http://1degreebio.org
http://www.equator-network.org/reporting-guidelines/improving-bioscience-research-repor
http://grants.nih.gov/grants/olaw/olaw.htm
http://www.mrc.ac.uk/Ourresearch/Ethicsresearchguidance/Useofanimals/index.htm
http://ClinicalTrials.gov
http://www.consort-statement.org
http://www.consort-statement.org/checklists/view/32-consort/66-title
http://www.equator-network.org/reporting-guidelines/reporting-recommendations-for-tumo
http://datadryad.org
http://figshare.com
http://www.ncbi.nlm.nih.gov/gap
http://www.ebi.ac.uk/ega
http://biomodels.net/
http://biomodels.net/miriam/
http://jjj.biochem.sun.ac.za
https://osp.od.nih.gov/biosafety-biosecurity-and-emerging-biotechnology/
http://www.selectagents.gov/

Reporting Checklist For Life Sciences Articles (Rev. June 2017)

This checklist is used to ensure good reporting standards and to improve the reproducibility of published results. These guidelines are consistent with the Principles and Guidelines for Reporting Preclinical Research issued by the NIH in 2014. Please follow the journal's authorship guidelines in preparing your manuscript.

A- Figures
1. Data
The data shown in figures should satisfy the following conditions:

- ➔ the data were obtained and processed according to the field's best practice and are presented to reflect the results of the experiments in an accurate and unbiased manner.
- ➔ figure panels include only data points, measurements or observations that can be compared to each other in a scientifically meaningful way.
- ➔ graphs include clearly labeled error bars for independent experiments and sample sizes. Unless justified, error bars should not be shown for technical replicates.
- ➔ if n< 5, the individual data points from each experiment should be plotted and any statistical test employed should be justified
- ➔ Source Data should be included to report the data underlying graphs. Please follow the guidelines set out in the author ship guidelines on Data Presentation.

2. Captions

Each figure caption should contain the following information, for each panel where they are relevant:

- ➔ a specification of the experimental system investigated (eg cell line, species name).
- ➔ the assay(s) and method(s) used to carry out the reported observations and measurements
- ➔ an explicit mention of the biological and chemical entity(ies) that are being measured.
- ➔ an explicit mention of the biological and chemical entity(ies) that are altered/varied/perturbed in a controlled manner.

- ➔ the exact sample size (n) for each experimental group/condition, given as a number, not a range;
- ➔ a description of the sample collection allowing the reader to understand whether the samples represent technical or biological replicates (including how many animals, litters, cultures, etc.).
- ➔ a statement of how many times the experiment shown was independently replicated in the laboratory.
- ➔ definitions of statistical methods and measures:
  - common tests, such as t-test (please specify whether paired vs. unpaired), simple χ2 tests, Wilcoxon and Mann-Whitney tests, can be unambiguously identified by name only, but more complex techniques should be described in the methods section;
  - are tests one-sided or two-sided?
  - are there adjustments for multiple comparisons?
  - exact statistical test results, e.g., P values = x but not P values < x;
  - definition of 'center values' as median or average;
  - definition of error bars as s.d. or s.e.m.

Any descriptions too long for the figure legend should be included in the methods section and/or with the source data.

In the pink boxes below, please ensure that the answers to the following questions are reported in the manuscript itself. Every question should be answered. If the question is not relevant to your research, please write NA (non applicable). We encourage you to include a specific subsection in the methods section for statistics, reagents, animal models and human subjects.

B- Statistics and general methods

Please fill out these boxes ↓ (Do not worry if you cannot see all your text once you press return)

1.a. How was the sample size chosen to ensure adequate power to detect a pre-specified effect size?	No prior analysis was applied to compute the sample sizes and power, however, our previous studies indicated that the sample sizes used here in this study would be sufficient to detect changes with high reproducibility.
1.b. For animal studies, include a statement about sample size estimate even if no statistical methods were used.	our previous studies indicated that the sample sizes used here in this study would be sufficient to detect changes with high reproducibility. The behavioral results are highly reproducible in independent experiments with high number of mice, suggesting that our study is well-powered. For the large scale gene expression studies, power depends on the gene variability across samples, sample size, RNA sequencing methodologies, and the contribution of related biological and technical covariates. This would require extensive modeling and adjustments with many assumptions to determine sample size and power. Therefore, we chose to demonstrate our results are highly reproducible in independent experiments, that empirically indicates that our study is reasonably well-powered.
2. Describe inclusion/exclusion criteria if samples or animals were excluded from the analysis. Were the criteria pre-established?	For sequencing data analysis, outlier samples were excluded using a previously described approach. A quality z-score which was calculated for each sample, and samples with low quality (Z > 2.5 or Z <-2.5) were identified as outlier and removed from further analysis. Moreover, samples with low quality Z score (Z > 2.5 or Z <-2.5) of eigenvalue were filtered out for downstream comparative analysis between conditions.
3. Were any steps taken to minimize the effects of subjective bias when allocating animals/samples to treatment (e.g. randomization procedure)? If yes, please describe.	mice were randomly allocated into different treatment/experimental groups.
For animal studies, include a statement about randomization even if no randomization was used.	mice were randomly allocated into different treatment/experimental groups.
4.a. Were any steps taken to minimize the effects of subjective bias during group allocation or/and when assessing results (e.g. blinding of the investigator)? If yes please describe.	Experimental mice were randomly allocated into treatment groups. Experimenter was blinded during stereotaxic injections. Behaviourial Results are analyzed by independent experimenters. To reduce subjective bias for behaviourial results and to draw most information from the behaviour test, MUST-C alogorithm based advanced ananlytical approach (see Methods for details) has been implemented.
4.b. For animal studies, include a statement about blinding even if no blinding was done	Please find the details in 4a. Additionally, Experimenters were blinded during animal tissue dissection, sequencing library preparation and qPCR based experiments.
5. For every figure, are statistical tests justified as appropriate?	yes

Do the data meet the assumptions of the tests (e.g., normal distribution)? Describe any methods used to assess it.	Data analyzed in this study was evaluated with Sahpiro-Wilk test for normal distribution. For non-parametric distribution, appropriate tests (e.g. Wilcoxon-rank sum, Kruskal Wallis) have been employed. Figure legend clearly states the description of tests applied for a given analysis.
Is there an estimate of variation within each group of data?	yes
Is the variance similar between the groups that are being statistically compared?	Variance between groups have been tested with F-test. In case of unequal variences, non-parametric tests have been applied. Please find the details in corresponding figure legends.

C- Reagents

6. To show that antibodies were profiled for use in the system under study (assay and species), provide a citation, catalog number and/or clone number, supplementary information or reference to an antibody validation profile. e.g., Antibodypedia (see link list at top right), 1DegreeBio (see link list at top right).	Synaptophysin 1 (guinea pig, SySy, cat 101004), PSD-95 (rabbit, Cell Signaling, cat 2507S). antibodies Cy3 (donkey, anti- guinea pig, Jackson Imm., cat 706-165-148), Abberrior STAR 635p (goat, anti-rabbit, cat ST635P)
7. Identify the source of cell lines and report if they were recently authenticated (e.g., by STR profiling) and tested for mycoplasma contamination.	Immortalized microglia cells (IMG) were purchased from Merck (Cat. No. SCC134) and tested negative for mycoplasma contamination using PCR Mycoplasma Test Kits (PromoCell).

* for all hyperlinks, please see the table at the top right of the document

D- Animal Models

8. Report species, strain, gender, age of animals and genetic modification status where applicable. Please detail housing and husbandry conditions and the source of animals.	Wild type C57/B6J mice (male) and transgenic APP/PS1 (male and female) have been used in this study. Pregnant mouse was from CD-1 background. All animals were housed in standard cages on 12h/12h light/dark cycle with food and water ad libitum. In addition to newborn pups, mice from from different ages (3-16.5 months) were used for the experiment. Please find specific details in corresponding figure legend.
9. For experiments involving live vertebrates, include a statement of compliance with ethical regulations and identify the committee(s) approving the experiments.	All experimental protocols were approved by a local animal care protocol.
10. We recommend consulting the ARRIVE guidelines (see link list at top right) (PLoS Biol. 8(6), e1000412, 2010) to ensure that other relevant aspects of animal studies are adequately reported. See author guidelines, under 'Reporting Guidelines'. See also: NIH (see link list at top right) and MRC (see link list at top right) recommendations. Please confirm compliance.	The animal experiments have been performed following ethical guidelines set by local Animal Welfare Organization (LAVES).

E- Human Subjects

11. Identify the committee(s) approving the study protocol.	All experiments related to humans reported in this study were approved by the local ethics committee.
12. Include a statement confirming that informed consent was obtained from all subjects and that the experiments conformed to the principles set out in the WMA Declaration of Helsinki and the Department of Health and Human Services Belmont Report.	Informed consent was obtained from all subjects and the experiments confirmed to the priniciples set out in the WMA Declaration of Helsinki and the Department of Health and Human services Belmont Report.
13. For publication of patient photos, include a statement confirming that consent to publish was obtained.	No patient photo is invovled in this study.
14. Report any restrictions on the availability (and/or on the use) of human data or samples.	Human data will be available via the European Genome-Phenome archive. Use of the human data/samples involved in this study is restricted for any commercial use.
15. Report the clinical trial registration number (at ClinicalTrials.gov or equivalent), where applicable.	N/A
16. For phase II and III randomized controlled trials, please refer to the CONSORT flow diagram (see link list at top right) and submit the CONSORT checklist (see link list at top right) with your submission. See author guidelines, under 'Reporting Guidelines'. Please confirm you have submitted this list.	N/A
17. For tumor marker prognostic studies, we recommend that you follow the REMARK reporting guidelines (see link list at top right). See author guidelines, under 'Reporting Guidelines'. Please confirm you have followed these guidelines.	N/A

F- Data Accessibility

18: Provide a "Data Availability" section at the end of the Materials & Methods, listing the accession codes for data generated in this study and deposited in a public database (e.g. RNA-Seq data: Gene Expression Omnibus GSE39462, Proteomics data: PRIDE PXD000208 etc.) Please refer to our author guidelines for 'Data Deposition'.<br><br>Data deposition in a public repository is mandatory for:<br>a. Protein, DNA and RNA sequences<br>b. Macromolecular structures<br>c. Crystallographic data for small molecules<br>d. Functional genomics data<br>e. Proteomics and molecular interactions	Data availability section is provided with the original manuscript.
19. Deposition is strongly recommended for any datasets that are central and integral to the study; please consider the journal's data policy. If no structured public repository exists for a given data type, we encourage the provision of datasets in the manuscript as a Supplementary Document (see author guidelines under 'Expanded View' or in unstructured repositories such as Dryad (see link list at top right) or Figshare (see link list at top right).	All Sequencing data is availave via GEO and EGA databases. Details are given in the "Data and code availability" section of the mansucript.
20. Access to human clinical and genomic datasets should be provided with as few restrictions as possible while respecting ethical obligations to the patients and relevant medical and legal issues. If practically possible and compatible with the individual consent agreement used in the study, such data should be deposited in one of the major public access-controlled repositories such as dbGAP (see link list at top right) or EGA (see link list at top right).	Human data will be availbe via EGA. Details are given in the "Data and code availability" section of the mansucript.
21. Computational models that are central and integral to a study should be shared without restrictions and provided in a machine-readable form. The relevant accession numbers or links should be provided. When possible, standardized format (SBML, CellML) should be used instead of scripts (e.g. MATLAB). Authors are strongly encouraged to follow the MIRIAM guidelines (see link list at top right) and deposit their model in a public database such as Biomodels (see link list at top right) or JWS Online (see link list at top right). If computer source code is provided with the paper, it should be deposited in a public repository or included in supplementary information.	Relevant information is desribed in the matrial and methods section. Code is avialbe via Github. Details are given in the "Data and code availability" section of the mansucript.

G- Dual use research of concern

22. Could your study fall under dual use research restrictions? Please check biosecurity documents (see link list at top right) and list of select agents and toxins (APHIS/CDC) (see link list at top right). According to our biosecurity guidelines, provide a statement only if it could.	No
